# Online Eye Tracking for Aphasia: A Feasibility Study Comparing Web and Lab Tracking and Implications for Clinical Use

**DOI:** 10.1002/brb3.70112

**Published:** 2024-10-29

**Authors:** Willem S. van Boxtel, Michael Linge, Rylee Manning, Lily N. Haven, Jiyeon Lee

**Affiliations:** ^1^ Department of Speech, Language, and Hearing Sciences Purdue University West Lafayette Indiana USA; ^2^ Department of Communication Sciences and Disorders Louisiana State University Baton Rouge Louisiana USA

**Keywords:** aphasia, clinical research, language comprehension, online eye tracking, webcam eye tracking, WebGazer

## Abstract

**Background & Aims:**

Studies using eye‐tracking methodology have made important contributions to the study of language disorders such as aphasia. Nevertheless, in clinical groups especially, eye‐tracking studies often include small sample sizes, limiting the generalizability of reported findings. Online, webcam‐based tracking offers a potential solution to this issue, but web‐based tracking has not been compared with in‐lab tracking in past studies and has never been attempted in groups with language impairments.

**Materials & Methods:**

Patients with post‐stroke aphasia (*n *= 16) and age‐matched controls (*n *= 16) completed identical sentence‐picture matching tasks in the lab (using an EyeLink system) and on the web (using WebGazer.js), with the order of sessions counterbalanced. We examined whether web‐based eye tracking is as sensitive as in‐lab eye tracking in detecting group differences in sentence processing.

**Results:**

Patients were less accurate and slower to respond to all sentence types than controls. Proportions of gazes to the target and foil picture were computed in 100 ms increments, which showed that the two modes of tracking were comparably sensitive to overall group differences across different sentence types. Web tracking showed comparable fluctuations in gaze proportions to target pictures to lab tracking in most analyses, whereas a delay of approximately 500–800 ms appeared in web compared to lab data.

**Discussion & Conclusions:**

Web‐based eye tracking is feasible to study impaired language processing in aphasia and is sensitive enough to detect most group differences between controls and patients. Given that validations of webcam‐based tracking are in their infancy and how transformative this method could be to several disciplines, much more testing is warranted.

## Graphical abstract

This study sought to test the feasibility of webcam‐based, at‐home eye tracking for use with aphasia patients. Results show that webcam‐based eye tracking is feasible and desirable for use in clinical research.

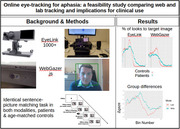

## 1 Introduction

Visual attention is strongly intertwined with language comprehension and production, making eye tracking a critical tool for examining cognitive processes underpinning various linguistic tasks. In particular, eye tracking has proven essential to the study of acquired language disorders such as aphasia, as it allows a fine‐grained examination of real‐time processing without relying on their impaired verbal responses. A wealth of research in eye‐tracking studies of language processing and disorders has established a tight interplay between vision and language, and rapid developments in eye‐tracking technology have accompanied theoretical interest. Until recently, however, eye tracking always necessitated participants physically being present in a laboratory setting or being sent specialized equipment for tracking their eyes using individual laptops or PCs (Holmqvist et al. 2011; Carter and Luke 2020). Over the last few years, web‐based algorithms for eye tracking through webcams, without the need for special cameras, equipment, or extensive logistical considerations, have been developed (RealEye sp. z o.o. 2023; Papoutsaki et al. 2017). Although web‐based tracking offers the possibility of low‐cost, accessible eye tracking with far more diverse and wide‐ranging participant samples, concerns remain about its accuracy and temporal precision, especially in relation to language (Slim and Hartsuiker 2023). Linguistic processing occurs at the millisecond level (Nobre and McCarthy 1994), and any latency jitter or temporal delays present serious problems for the validity of experimental findings using web‐based eye tracking. Additionally, web‐based tracking is naturally limited in the sampling rates it achieves due to webcam frame rates, system variability, and internet connectivity issues, whereas in‐lab trackers can achieve sampling rates of up to 2000 Hz.

These methodological hurdles become even more pertinent when studying patients with aphasia (PWA). Aphasia is an acquired language disorder following brain injuries, most commonly stroke, affecting both language production and comprehension (see Clark and Cummings 2003; Coppens 2016). PWA generally exhibit larger within‐sample variability than healthy groups (Lazar and Antoniello 2008; Duncan, Schmah, and Small 2016), and experimental samples are generally small (e.g., Robey 1998). Obtaining accurate measurements from larger samples is therefore of critical importance. The application of web‐based eye tracking to the study of aphasia could potentially bring several benefits, including increases in sample sizes, wider geographic spreads in samples, and inclusion of participants from underrepresented backgrounds. Web‐based tracking has not been studied in aphasia before, and no comparisons between in‐lab and web‐based tracking in clinical samples exist (for the only clinical study of web‐based tracking to date, see Greenaway et al. 2024 in dementia patients). In this study, we present the first direct comparison of the quality, timing, and accuracy of in‐lab and web‐based eye tracking in aphasia and address the suitability of web‐based eye tracking to clinical studies in general.

### 1.1 Eye Tracking and Aphasia

Generally, listeners tend to look at what is being spoken about. Cooper (1974) was among the first authors to report on the connection between vision and language. After presenting participants with spoken narratives and pictures of items related to those stories, Cooper observed that people tend to direct their gaze toward items in the visual field that are semantically related to elements being discussed in spoken language. This experiment was subsequently developed into the visual world paradigm, a highly common task in eye‐tracking studies of language comprehension (see Tanenhaus and Trueswell 2006; Huettig et al. 2011, for a review). Eye movements have also been recorded during sentence‐picture matching tasks, where a spoken sentence has to be matched to one of several pictures depicting different events (e.g., Meyer, Mack, and Thompson 2012). Patterns in which listeners fixate different images can indicate the time course of how various linguistic information is decoded as their comprehension unfolds and where processing difficulty occurs. Eye movements can thus be used as reliable, sub‐behavioral indices of sentence processing.

Eye movements are generally recorded with a professional‐grade camera, with resolutions anywhere between 50 and 2000 Hz. For higher end tracking, an infrared illuminator is also required, which shines infrared light onto participants’ eyes and deducts gaze positions by the reflection of this light on the cornea (Holmqvist et al. 2011). These systems are difficult to transport and expensive to purchase, and participants must be present in person to take part, but they do yield highly informative data. Eye‐trackers capture when and where participants’ gazes land on spatial locations on screen (fixations), and when and where gazes switch between fixation points (saccades). In psycholinguistics, fixations are the dominant measurement used as indices of processing. Fixation analyses have elucidated the rapid time course of linguistic processing (Saslow 1967; Richardson and Spivey 2004; Van Boxtel et al. 2023) and have generated important findings regarding the nature of processes underlying both language production and comprehension. Eye movements measured before the onset of speech or before sentence elements that disambiguate between two competing interpretations may also reflect anticipatory processing (Vos et al. 2022; Griffin and Bock 2000). In this way, eye tracking provides valuable means to examine the time course of human language processing. This becomes even more significant in contexts of aphasia, an acquired language deficit resulting from damage to the brain.

Eye tracking in patients with aphasia (PWA) has been used to assess sentence comprehension (e.g., Thompson and Choy 2009; Schumacher et al. 2015; Meyer, Mack, and Thompson 2012), comprehension of multimodal information such as gesture interpretation (Eggenberger et al. 2016; Preisig et al. 2015, 2018), and sentence production (Cho and Thompson 2010; Lee and Thompson 2011; Lee, Yoshida, and Thompson 2015, 2020; Van Boxtel et al. 2023). Collectively, eye‐tracking studies elucidated novel findings on PWAs’ language processing that were difficult to study using off‐line accuracy measures. For example, some comprehension eye‐tracking studies have revealed that while automatic activation of syntactic reference is preserved in PWA, they struggle to integrate this activated information to determine final interpretations of complex sentences (Choy and Thompson 2010; Dickey et al. 2007). Eye‐tracking‐while‐speaking studies revealed that advanced planning of sentences in larger chucks is a crucial mechanism for successful sentence production in agrammatic aphasia (Lee and Thompson 2011; Lee, Yoshida, and Thompson 2015; Lee 2020). Eye‐tracking data can also serve as fine‐grained treatment outcome measures in PWA, revealing more normal‐like eye gaze patterns during sentence planning and comprehension after a language intervention (Mack, Nerantzini, and Thompson 2017; Mack and Thompson 2017). Thus, eye‐tracking methodology has strong potential to reveal underlying patterns of language processing in PWA, and continued use of eye tracking in aphasia and other related language disorders will remain fruitful.

### 1.2 Comparing Lab and Web Eye Tracking

Lab‐based eye‐tracking techniques offer precise, high‐resolution gaze estimations of participants’ eye movements by projecting infrared light onto the pupil or by building 3D eye models while tracking. Illumination of the pupil with infrared light creates a corneal reflection that can be used to calculate the angle of eye fixation (Vos et al. 2022). However, despite that laboratory eye trackers offer high resolution, lab‐based approaches involving clinical populations remain limited by geographical constraints hindering how many clinical participants may take part in lab‐based eye‐tracking studies. Indeed, lab‐based studies may require over double as many participants as is currently standard to obtain adequate statistical power (Slim and Hartsuiker 2023). This limitation bears important implications when considering the generalizability of lab‐based eye‐tracking studies. In the wake of the COVID‐19 pandemic, shifts toward internet‐based empirical measures have increased exponentially, including the implementation of virtual eye‐tracking paradigms (Slim and Hartsuiker 2023; Vos et al. 2022; Greenaway et al. 2024; Yang and Krajbich 2023). Virtual techniques offer great potential given that web‐based methods allow researchers to collect data from a broader, more diverse sample size with more efficiency and offer a less expensive alternative to lab tracking. However, remote eye‐tracking techniques remain in the early stages of development and it remains unclear to what extent web‐based results compare to those gathered in the lab, especially in clinical groups, which show higher intra‐sample variability.

In this study, we focus on one such technique. WebGazer, a JavaScript algorithm developed by Papoutsaki et al. (2017), uses real‐time face meshing through participants’ individual webcams to obtain gaze estimations on webpages. WebGazer detects pixels corresponding to participants’ eyes in every video frame and continuously builds regression models using these detected pixels, estimating the location of participants’ gazes on the screen. The library uses participant interactions (such as mouse movements or clicks) to more accurately estimate gaze locations, but in the absence of such interactions, the regression model uses only eye pixel data to infer gaze locations in real time (see https://webgazer.cs.brown.edu/ for more details). Importantly, WebGazer was recently integrated into Gorilla.sc (Anwyl‐Irvine et al. 2020), a commonly used online experiment platform. This removes the need for experimenters to manually code WebGazer into individual webpages, and opens up vast possibilities for recording eye movements during different experimental tasks. Whether WebGazer is suitable for linguistic experimental work has, however, not been comprehensively assessed, and especially its applicability to aphasia is unexplored.

The viability of WebGazer for psychological and psycholinguistic research has recently been explored in a number of studies. Generally, webcam‐based tracking has been found to be accurate and at least somewhat comparable to in‐lab tracking, but with reduced spatial and, especially, temporal accuracy (a reduction that varies considerably across studies). A comparison between in‐lab and web‐based tracking by Bogdan et al. (2024) showed that results obtained through participants’ own webcams were highly comparable to those obtained in the lab, with seven out of their eight emotion–attention interaction lab tasks being replicated on the web. Turning to language‐based tasks, WebGazer was found accurate in visual world paradigms (Slim and Hartsuiker 2023, Prystauka, Altmann, and Rothman 2024), story comprehension tasks (Hutt et al. 2024), and a morphological anticipation design (Özsoy et al. 2023). Overall, these studies endorse the quality of web‐based eye data across paradigms, producing results comparable to those of several in‐lab trackers (particularly those with comparatively low sampling rates, around 60 Hz). Common findings in psycholinguistics, such as gazes being attuned to sentential context in the visual world paradigm (Slim and Hartsuiker 2023) or preferential gazes to predicted objects (Özsoy et al. 2023), were fully replicated in healthy adults.

Web‐based eye tracking therefore seems to have great potential to supplement eye‐tracking research with a low‐cost, universally deployable method. Nevertheless, several drawbacks to web‐based tracking were also uncovered by the above studies. Despite generally high comparability between the lab and web, effect sizes on the web suffer considerably compared to the lab (with some studies finding effect sizes only about half the size of those from lab‐based results, e.g., Bogdan et al. 2024). Studies run on the web may therefore require more trials per experiment or larger participant samples. Further, Bogdan et al. (2024) discovered that WebGazer is biased toward reporting gaze estimations in the center of participants’ screens, recommending that trial screens should be symmetrical and areas of interest (AOIs) should not disproportionately fall into one half of the screen. WebGazer may further struggle to detect patterns in groups with higher internal sample variability (such as the heritage speakers of Turkish in Özsoy et al. 2023). This is especially important when studying clinical groups, who similarly show high levels of variability, mainly due to each patient's individual pathology.

One of the most commonly reported issues with web‐based tracking is its poor temporal performance compared to in‐lab methods. For instance, Slim and Hartsuiker (2023) examined healthy adults in a visual world paradigm, where participants predicted the final words of auditory sentences by looking at one of four objects presented on screen before the final word was reached (e.g., for “Mary reads a letter,” three semantic foil objects and a target “letter” drawing were presented). The authors reported a recording delay of approximately 300 ms compared to past in‐lab tracking results, which, although relatively consistent, nevertheless causes concern for research into language processing, which unfolds at the millisecond level. Even when participants do not perform an actual task but simply go through calibration procedures, delays on the web are apparent: Semmelmann and Weigelt (2018) observed a rough duration of 600 ms to execute one saccade during calibration, nearly triple the known average of 200 ms (Matin, Shao, and Boff 1993). Temporal lag may be caused by slower browser speeds or delayed internal processing of WebGazer. Additionally, variation in participants’ machines, monitor size, webcam resolution, internet connectivity, and physical environment may affect the quality of web‐based tracking (Semmelmann and Weigelt 2018; Slim and Hartsuiker 2023). Although these elements may be controlled in a laboratory setting, webcam tracking offers less control (e.g., Papoutsaki et al. 2018; Semmelmann and Weigelt 2018; Wisiecka et al. 2022). Crucially, consistent reports of timing delays in web‐based eye‐tracking algorithms may limit their capacity to investigate fine‐grained temporal patterns of language processing. However, this has not been demonstrated conclusively.

Beyond these temporal aspects, it remains in question whether webcam eye tracking is suitable for research in communication disorders, as clinical populations may demonstrate more inherent variability and pathologically delayed processing of linguistic information. To our knowledge, only Greenaway et al. (2024) have so far used webcam‐based eye tracking with patients with dementia, and none have directly compared in‐lab and web‐based tracking. Greenaway et al. (2024) examined the internal consistency of attentional biases in a group of 12 patients with Alzheimer's disease. Patients spent time looking at happy, sad, angry, and neutral face pictures, and time spent looking at emotional faces was subtracted from time spent looking at neutral faces. Despite their relatively small sample size, reliable attentional bias patterns were discovered using web‐based eye tracking, similar to the previous findings from in‐lab studies, suggesting that web‐based tracking is indeed possible with clinical groups at least in non‐linguistic domains. However, it remains completely unexplored whether web‐based tracking is sensitive enough to feasibly gage conditional differences in the language domain (e.g., differences in processing various structures such as actives or passives) in clinical populations. As discussed above, patients with aphasia show impairments in comprehending complex sentences such as passives and those with padded locatives (e.g., “The bird was next to the fence beside the rock.”). These impairments can manifest on sentence‐picture matching tasks as reduced accuracy, longer response times, and chance‐level looks to target pictures (e.g., Meyer, Mack, and Thompson 2012; Mack, Meltzer‐Asscher, et al. 2013; Mack et al. 2016). To be sufficiently sensitive for use in clinical populations, web‐based eye tracking should be capable of recording processing differences between these structures. If WebGazer is indeed as accurate as some past studies suggest, even in patients with aphasia, web‐based eye tracking has the potential to revolutionize the fields of speech‐language pathology and related clinical linguistics.

Thus, as virtual research continues to expand, this study aims to determine the feasibility of web‐based eye tracking for research in aphasia. Specifically, we compared web‐based and in‐lab eye‐tracking patterns from a sentence‐picture matching task with the same patients with aphasia (PWA) and healthy controls in both modalities (1) to assess whether both tracking modes are sensitive to uncovering group differences between PWA and controls, and (2) to determine whether web‐based eye tracking is sensitive to differences between PWA and controls by sentence structure type. To our present knowledge, this is the first study to compare the sensitivity of lab‐ and web‐based eye tracking to language comprehension effects within the same groups of participants, as well as the first to conduct remote eye tracking in PWA. We hypothesized that (1) PWA would be less accurate and take longer to respond to all sentence types than controls, with greater group differences in passive and locative sentences (see, e.g., Mack, Nerantzini, and Thompson 2017); (2) PWA should show lower levels of gazes to the target picture compared to controls in both the lab and the web (Van Boxtel et al. 2023; Slim and Hartsuiker 2023); and (3) these group differences should be stronger for passives and locatives compared to actives.

## 2 Methods

### 2.1 Participants

Seventeen persons with post‐stroke aphasia (PWA) and 16 age‐matched controls took part in one in‐lab and one web‐based testing session with at least 2 weeks apart. One PWA was excluded due to repeated calibration issues both on the web and in the lab; thus, the data reported henceforth include 16 participants per group. Six PWA and one control completed the web‐based eye‐tracking task in the lab due to lack of access to adequate equipment or the absence of a caretaker to assist with technical setup at home. The study was approved by the local Institutional Review Board. All participants provided informed consent prior to study participation and received monetary compensation after completion of the study.

All participants passed hearing and vision screenings and were monolingual native speakers of North American English with little to no knowledge of other languages. All control participants scored within normal limits on the all tests of Cognitive‐Linguistic Quick‐Test + (CLQT+; Helm‐Estabrooks 2017), indicating no atypical changes in cognitive‐linguistic skills. All participants reported no history of neurological (other than stroke) conditions, psychiatric disorders, or history of speech‐language or learning disabilities besides aphasia. Control and PWA groups did not differ in terms of age (*t *= −0.988, *p* > 0.05), and both groups spent roughly equal numbers of years in education, as shown in Table 1 (*t *= −0.817, *p* > 0.05).

**TABLE 1:** Overview of participant demographics.

				WAB		
Group	Age	Years in education	Gender	AQ	SS	AC
Control	58.2 (12.02)	16.9 (1.34)	11 Female 5 Male	N/A	N/A	N/A
PWA	62.9 (11.99)	15.3 (2.44)	7 Female 9 Male	70.4 (21.8)	12.8 (1.73)	8.47 (1.73)

*Note*: Figures represent means, with standard deviations in brackets.

Abbreviations: AC, auditory comprehension (max = 10); AQ, aphasia quotient (range 0–100); SS, spontaneous speech (max = 20); WAB, Western Aphasia Battery (Kertesz 2007).

Patients with aphasia (PWA) required a diagnosis of aphasia resulting from a left‐hemisphere stroke, with no less than 6 months intervening between stroke onset and study participation. On average, PWA was 68.2 months post‐stroke onset (ranged from 7 to 195 months). All PWA were tested on the Western Aphasia Battery—Revised (WAB‐R; Kertesz 2007) to assess their aphasia severity. Table 1 reports the scores of PWA on the spontaneous speech (SS) and auditory comprehension (AC) sections of the WAB. Scores on the WAB can also be concatenated to an aphasia quotient (AQ): AQs below 25 signify the presence of very severe aphasia, AQs of 26–50 are classified as severe, AQs of 51–75 are moderate, and AQs above 76 are considered mild. PWA in this study presented with severe or mild aphasia with AQs ranging from 38.2 to 94. PWAs’ speech production and comprehension varied, but all PWA demonstrated ability to produce and comprehend some single words and simple sentences. PWA participants further completed selected subtests of the CLQT (Helm‐Estabrooks 2017): The Symbol Cancellation subtest was used to screen for hemi‐spatial neglect and visual attention impairments, and the Design Memory subtest was used to screen for general non‐verbal cognitive deficits. The PWA who scored at least 8/12 and 4/6 on these subtests, respectively, were included in the study.

### 2.2 Materials

**FIGURE 1:**
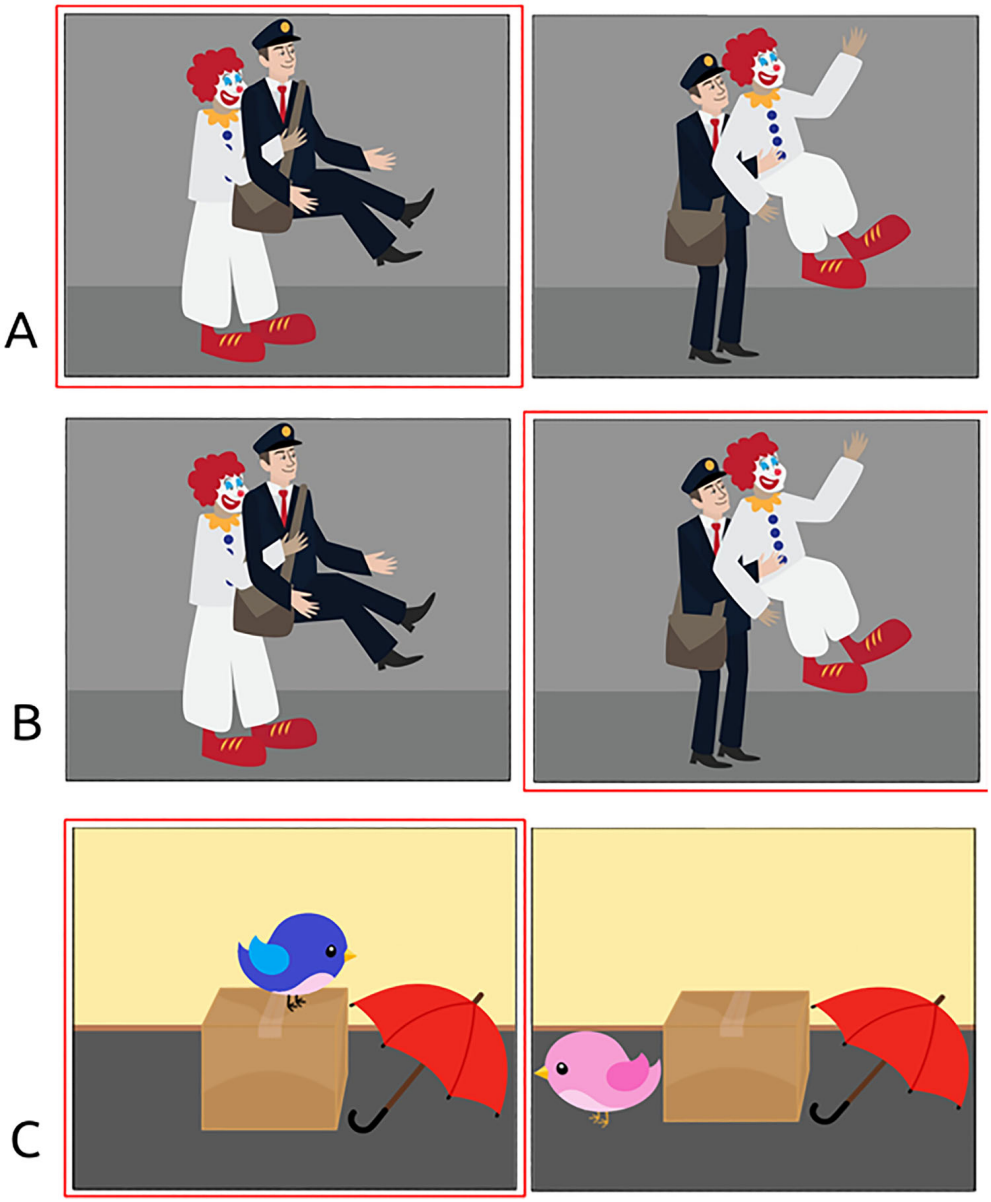
Example target and foil pictures for each sentence type (active, A; passive, B; locative, C) matching the examples given in Table 2.

Two identical auditory sentence‐picture matching tasks were designed that paired two images with passive, active, locative, or intransitive (filler) questions. The web version was designed in Gorilla.sc (Anwyl‐Irvine et al. 2020), whereas the lab version was built in SR Research Experiment Builder (SR Research, Missisauga, Ontario). A total of 65 trials were designed, including 15 active (“Who is lifting the mailman here?”) and 15 passive trials (“Who is lifted by the mailman here?”) to test whether different processing strategies for canonical (active) and non‐canonical (passive) sentence structures are shown on web‐ and lab‐based eye tracking. The 15 trials used as actives in the lab version were used as passives in the web version, and vice versa. Thus, although the lab version included the example in Figure 1 as “Who is lifting the mailman here,” the web version presented the same trial as “Who is lifted by the clown here?.” Additionally, 15 sentences with 2 locative prepositional phrases (e.g., “Which bird is on the box next to the umbrella?”) were included to evaluate processing of locative relations. Finally, 20 filler items were included, consisting of 10 declaratives (“The man is cleaning. Which one?”) and 10 wh‐questions (“Which dog is digging?”). All fillers were intransitive, relatively simple structures, and the same fillers and locatives were used across both task versions. Examples of experimental sentence types and critical disambiguation regions are shown in Table 2 and Figure 1.

**TABLE 2:** Example sentence items and disambiguation points.

Sentence type	Example	Disambiguation point	Figure 1 example
Active	Who is lifting the mailman here?	Mailman	A
Passive	Who is lifted by the mailman here?	Mailman	B
Locative	Which bird is on the box next to the umbrella?	On the box	C

Audio recordings were made by a female native speaker of North American English and normalized for audio volume at 70 dB. Onsets of words in each sentence type were marked using PRAAT software (Boersma and Van Heuven 2001), and sentence disambiguation points were logged using these onset times. For actives and passives, disambiguation was defined as the onset of the noun (“Who is lifting the *mailman* here?”), whereas for locatives, the disambiguation point was the onset of the first preposition (“Which bird is *on* the chair by the window?”). This allowed for the analysis of the timing of looks to the correct picture relative to disambiguation time.

**FIGURE 2:**
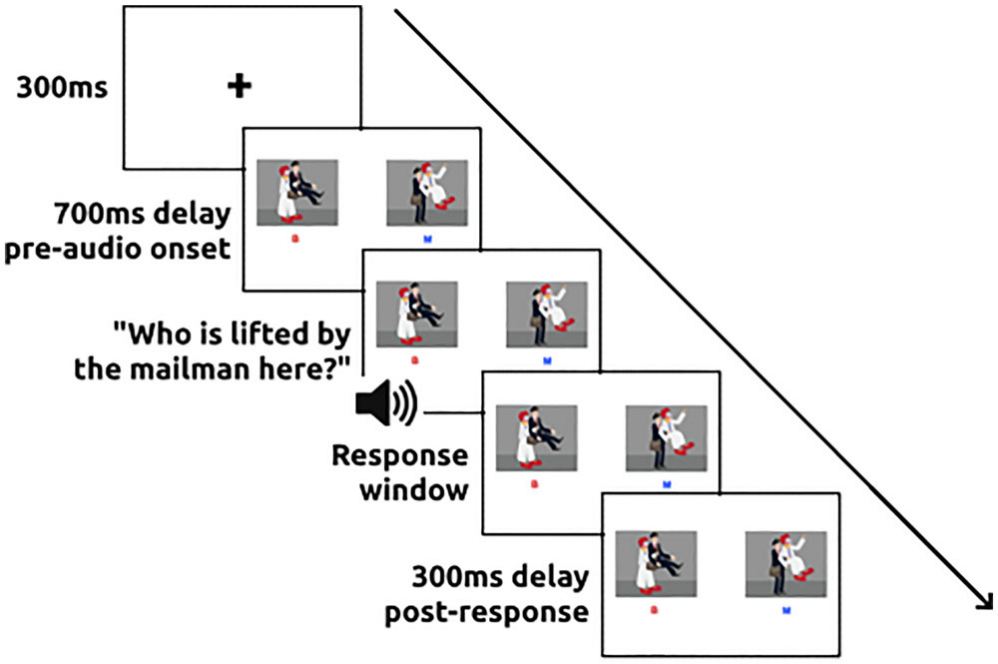
Schematic overview of task sequence. Trials began with a 300 ms fixation cross followed by a 700 ms apprehension phase before audio recordings played. The response window opened upon conclusion of the audio recording and closed upon a button press, and a 300‐ms delay to account for potential timing inaccuracies in the web experiment followed.

As shown in Figures 1 and 2, in each trial, one of two presented images was the target (depicting the correct action), whereas the other was a foil picture depicting the opposite action. For example, for the question “Who is lifted by the mailman here?,” the target showed a mailman lifting a clown, and the foil showed the clown lifting the mailman. The target picture was shown on the right and left sides of the screen in 50% of trials each, and colored letters reminding participants that buttons to press (“B” for the left and “M” for the right picture) were shown on screen for each trial. See our Supporting Information at https://osf.io/n6gu7/ for a full stimuli list.

### 2.3 Procedure

Task procedures were kept the same in the web and lab versions wherever possible and generally adhered to Holmqvist et al.'s (2023) best practice guidelines for conducting and reporting eye‐tracking research.

#### 2.3.1 Eye‐Tracking Task

Participants completed an auditory sentence‐to‐picture matching task. Three practice trials were presented before the start of the experimental trials. Participants were given the opportunity for a break between each block (after 13 trials), which were also used for recalibration and validation (see Sections 2.3.2 and 2.3.3). Participants were only recalibrated and revalidated when required during the in‐lab task (as deemed by the experimenter), whereas the web version recalibrated between each block. The main task took most participants between 20 and 30 min to complete.

As demonstrated in Figure 2, trials began with a 300 ms fixation cross that focused participants’ attention to the center of the screen. The two trial pictures (target, the correct item, and foil, the incorrect item) were then presented for 700 ms before the audio recording started playing. This apprehension phase was designed to allow participants to appraise the scene before auditory processing began (see Griffin and Bock 2000; Mack, Nerantzini, and Thompson 2017). Then the participant heard the target sentence played through the speakers. Upon hearing the sentence, the participant pressed a correct keyboard button to identify the target picture. The response window did not open until the audio recording had finished. Additional 300 ms delay followed each button response. This post‐response delay was included given Slim and Hartsuiker's (2023) finding of consistent delays in WebGazer's timing of around 300 ms and aimed to prevent such delays from inhibiting the recording of eye gazes before the trial ended.

#### 2.3.2 In‐Lab Setup

For the lab task, participants’ eye movements were recorded at 500 Hz using an EyeLink 1000+ system (SR Research, Missisauga, Ontario) running in monocular remote (unstabilized) mode, in a room with constant lighting conditions and no background movement. SR Research claims typical recording accuracy of 0.25–0.5° in remote‐mode recording. Participants were seated approximately 65 cm from the presentation screen, and those who required glasses wore these during the experiment. The standard EyeLink calibration and validation procedure was followed using a nine‐point grid. EyeLink validation standards suggest validation to match calibration within 1.0°, which was followed wherever possible. Four PWA were tested using an arm‐mounted tracker (where a PC screen and camera are attached to a free‐moving hydraulic arm that can be moved around in front of the participant's face), whereas all other participants were recorded with a desktop‐mounted camera due to limitations on participants’ ability to be tested at different laboratory sites. Participants viewed the experiment on a 24 in., 1920 × 1080 pixels monitor with a refresh rate of 60 Hz, running Windows 10 Pro. The in‐lab experiment was presented using Experiment Builder (SR Research, Missisauga, Ontario). Responses were given on a wired Microsoft keyboard, and where necessary for PWA, colored stickers were overlaid on the B and M keys to indicate the response keys.

#### 2.3.3 Web‐Based Setup

**FIGURE 3:**
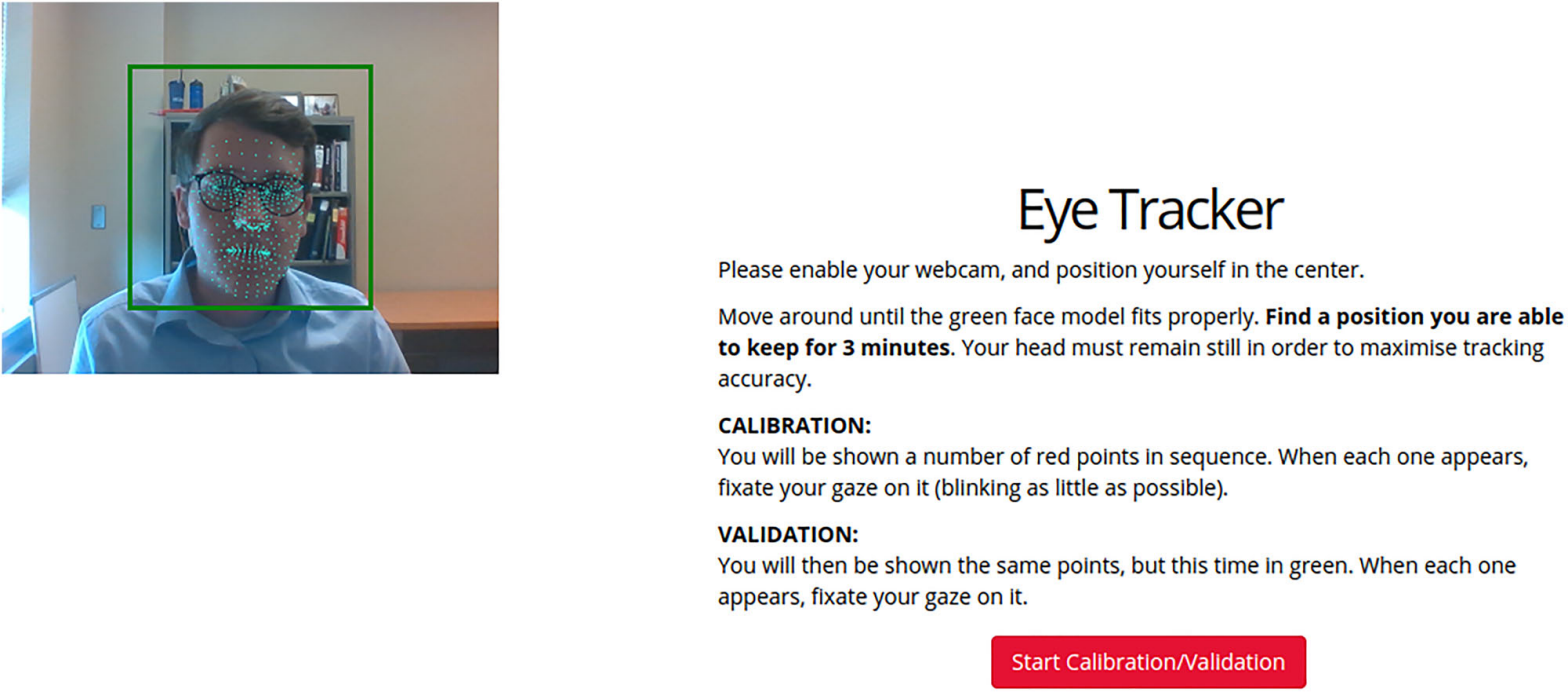
Gorilla.sc and WebGazer camera set‐up screen. Participants were prompted to position their heads in the center of the green box, for their rooms to be well‐lit and their poses to be comfortable to maintain for several minutes.

**FIGURE 4:**
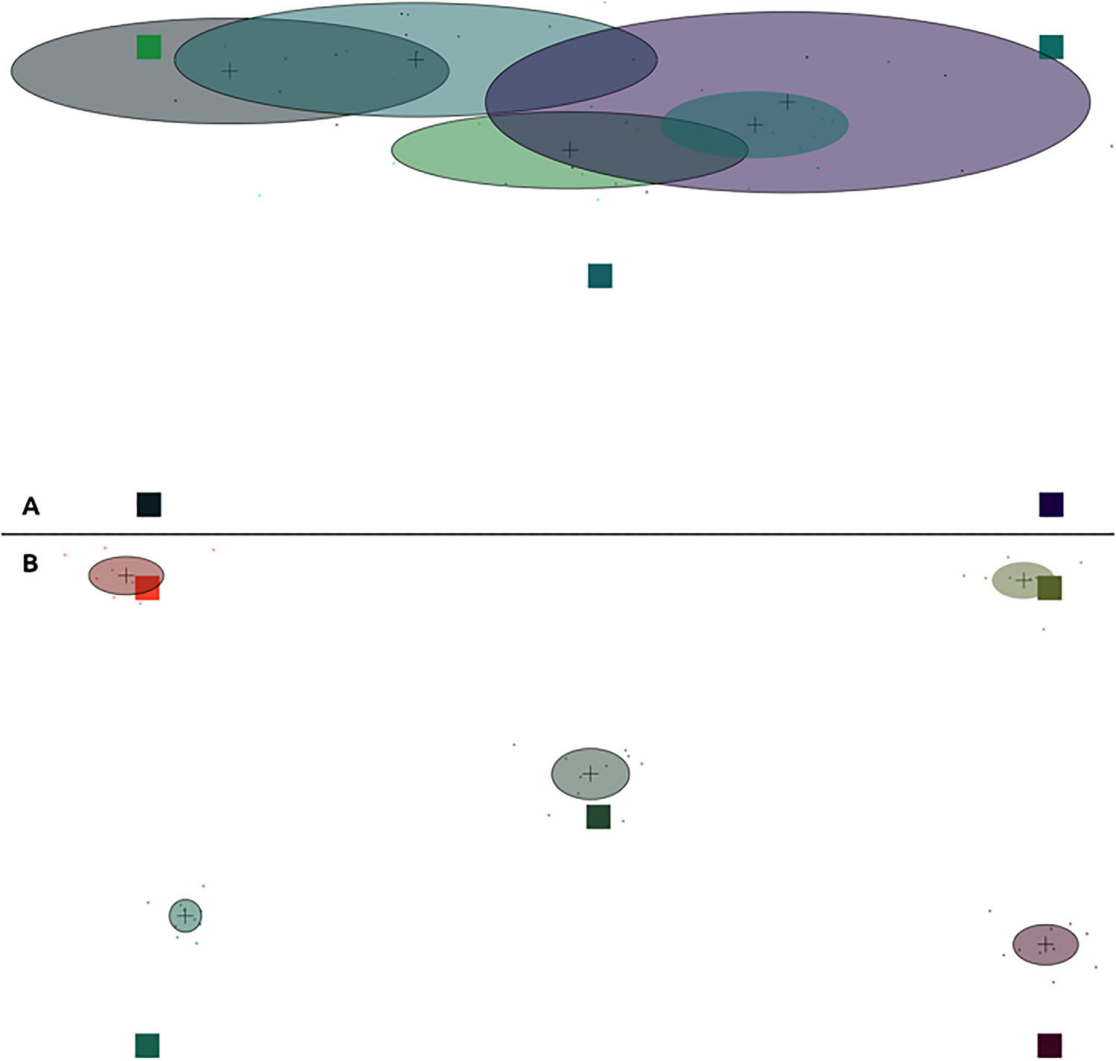
Example of a failed (A) and successful (B) calibration using WebGazer. Individual dots represent gaze estimations, and colors indicate which calibration and validation matches each point.

On the web, participants’ webcams were used to obtain eye movement recordings. A video set‐up screen (see Figure 3) preceded the calibration screen. However, on this set‐up screen, participants were expressly instructed to keep their face in a centered position and to try not to move their heads or bodies during the study. Once centered in the video recording box, WebGazer applied a green mesh onto the participant's face (see Figure 3). Face meshing was generally successful without camera or lighting adjustments, but in cases where participants’ faces were not meshed correctly, adjustments of lighting conditions made meshing successful in all cases. Reducing backlight and increasing the amount of light on the front of participants’ faces generally aided successful face meshing. Following this setup, WebGazer automatically calibrated in Gorilla without direct experimenter control. WebGazer considers a calibration inaccurate when any one calibration and validation point is located closer to a different calibration point than the intended point. When calibration was deemed inaccurate, participants were prompted to try again until properly calibrated. See Figure 4 for examples of failed (Figure 4A) and successful (Figure 4B) calibration with colors indicating different calibration points. Participants conducted the experiment using their own laptops or desktop computers and provided responses with their own hardware. Experimenters accessed remote control of the participants’ screens on a videocall to guide them through the experiment. Where necessary to improve bandwidth or prevent interference between requested webcam access from Gorilla and the videocall, participants’ video feeds were turned off during the experiment.

#### 2.3.4 Areas of Interest

**FIGURE 5:**
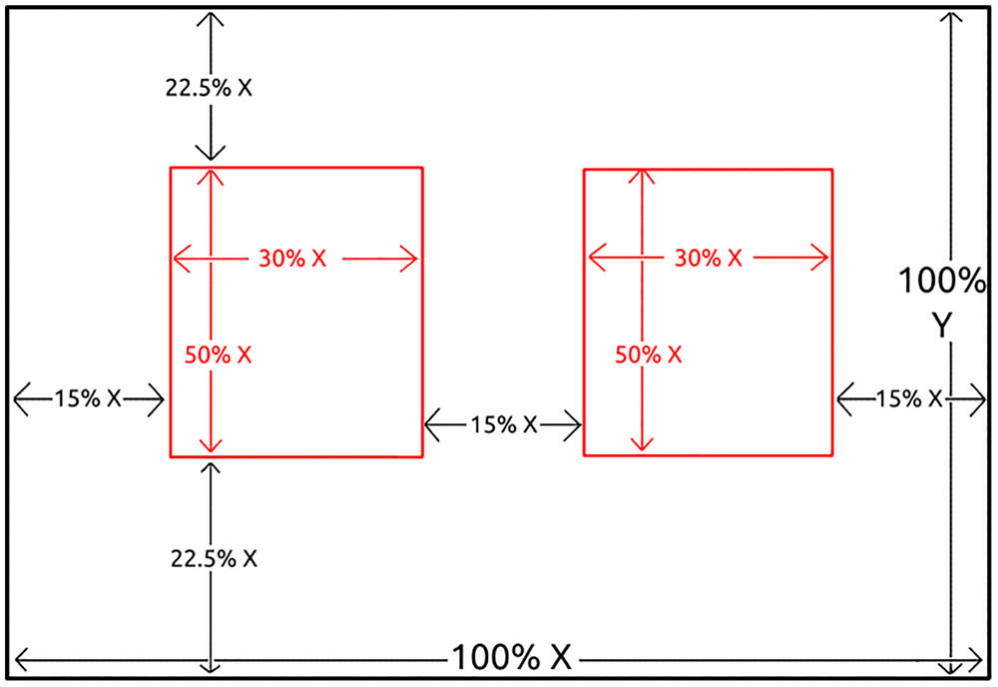
Diagram of viewport dimensions used to calculate areas of interest (AOIs) for the web‐based task. Proportions indicate viewport size in pixels. For instance, on a 1920 × 1080 pixels screen the left AOI began at 0.15 × 1920 = 288 pixels and ended at (0.15 + 0.30) × 1920 = 864 pixels along the *X*‐axis, and along the *Y*‐axis began at 0.225 × 1080 = 243 pixels and ended at (0.225 + 0.5) × 1080 = 783 pixels. A sample with coordinates 480*x* (25%), 540*y* (50%), would therefore be assigned to the left AOI.

In the lab, AOIs were defined in Experiment Builder, surrounding target and foil pictures and including the “B” and “M” letters used to fully capture participants’ eye fixations through the end of the post‐response delay (see Figure 2). Data Viewer automatically generates reports based on whether gaze positions overlap either AOI, allowing for relatively easy region‐based analysis. However, it is thus far impossible to pre‐specify AOIs in WebGazer. Therefore, for the web‐based experiment, we calculated AOIs post hoc, using proportions of participants’ viewport size in pixels to define AOIs. Gorilla scales and resizes picture objects depending on the monitor and viewport size it detects, and as such, we calculated AOIs for each participant individually. Because WebGazer outputs x and y coordinates for each eye gaze sample, samples could then be overlaid with the calculated AOIs to determine into which AOI they fell, if any. The AOI for the left picture started along the *X*‐axis at 15% of the participant's screen width and ended at 45%, whereas the AOI for the right picture began at 55% and ended at 85% of the screen. On the *Y*‐axis, AOIs ranged from 22.5% through 77.5% of the screen height. See Figure 5 for a proportional diagram of ROI calculation in the web‐based task. We allowed for larger AOIs in the web‐based compared to the in‐lab experiment as we hypothesized WebGazer to show much poorer spatial precision than EyeLink.

### 2.4 Analysis

#### 2.4.1 Variables

Behavioral (button press) responses were logged either as correct (when the target picture was selected) or incorrect (selection of the foil picture), constituting our *accuracy* dependent variable. The time it took participants to respond to the trial from the onset of the trial was defined as our *reaction time* (RT) variable, which was further square‐root‐transformed for additional normality. Accuracy and RT were the first of three variables we analyzed, the final one being eye gaze data. In‐lab eye data were extracted using SR Research Data Viewer (SR Research, Missisauga, Ontario), whereas web‐based WebGazer data were extracted and preprocessed manually. Although linguistic and cognitive science paradigms have commonly used *fixations* to assess processing (collected gaze estimations to a single point on screen, computed in a number of different ways depending on the event detection algorithm used; see Holmqvist et al. 2011, ch. 5, for an overview), WebGazer does not output fixations and does not include an algorithm for computing fixations manually. To keep variables between the lab and web modalities as equal as possible, we therefore decided to use individual raw gaze location estimates as main dependent variable for *both* eye‐tracking modalities (gaze estimations are generally used by preprocessing algorithms to compute fixations or saccades). While increasing computational cost significantly, this still allowed for the accurate measuring of time spent looking at either picture because estimations take place a set interval of time after the previous estimation. Averaged across all in‐lab data, one gaze estimation occurred every 2.3 ms (resulting in an effective resolution of 433.1 Hz; SD = 13.91 ms), whereas WebGazer recorded one estimation every 49 ms (and thus, at 13.5 Hz; SD = 162.2 ms). Each EyeLink gaze estimation was automatically labeled as occurring in either the Target or the Foil AOI (or neither, in which case the estimation was discarded), whereas WebGazer estimations were assigned to either the Target or Foil depending on the AOI computation shown in Figure 5 and whether the Target was presented on the left or right side of the screen in that particular trial.

Before analysis, we trimmed all estimations with a timestamp more than two standard deviations removed from the mean of timestamps in each trial to improve the distribution of the data (trial duration varied significantly across participants and trials, such that adequate trimming was necessary to make informed comparisons in later stages of the response window). Accuracy data were not trimmed prior to analysis, although RT data were trimmed such that all inaccurate trials and all remaining trials with an RT more than two standard deviations removed from each participant and each session's mean were removed. In total, 17.8% of the RT data were trimmed prior to analysis (including outliers and incorrect trials), and 5.3% of the eye data. Applying mixed‐effects regression to individual gaze estimations nevertheless turned out to be impractical due to the very high number of observations recorded (over 4.5 million for a 32‐participant dataset) and may have led to confounds with statistical power given that the lab modality contained many more samples than data from the web. We therefore subdivided the data into discrete 100 ms bins to reduce the computational cost of the analysis process while maintaining temporal accuracy. In each trial and each bin (ranging from 0–100 to 11,900–12,000 ms), we computed the proportion of gaze estimations to the target picture out of all gaze estimations in that bin, and this proportion was used as our dependent variable. Time bins where no samples were recorded, either due to missing recording time or following trimming, were removed before analysis. For the full code used for this preprocessing pipeline, refer to our scripts in the Supporting Information (https://osf.io/n6gu7/).

#### 2.4.2 Modeling and Statistics

All data were processed and analyzed in R 4.3.0 (R Core Team 2023) and RStudio version 2023.6.0.421 (Posit Team 2023) running on a 64‐bit Ubuntu system. We used the *lme4* (Bates et al. 2015), *lmerTest* (Kuznetsova, Brockhoff, and Christensen 2017), *GLMMadaptive* (Rizopoulos 2023), *DHARMa* (Hartig 2022), *betareg* (Cribari‐Neto and Zeileis 2010), and *pscl* (Jackman 2020; Zeileis, Kleiber, and Jackman 2008) libraries to fit regression models. Additionally, we evaluated marginal means using *emmeans* (Lenth 2023) and generated effect sizes using *MuMIn* (Barton 2023). General data preparation and visualization were conducted in the *tidyverse* (Wickham et al. 2019), using *ggplot2* (Wickham 2016), *data.table* (Dowle and Srinivasan 2023), and *stringr* (Wickham 2022). Confirmatory Bayesian models, where appropriate, were fitted in *brms* (Bürkner 2017) and compared using *bridgesampling* (Gronau, Singmann, and Wagenmakers 2017). First, we aimed to evaluate processing differences between PWA and controls, and as such added fixed effects of group to our models. Second, to assess differences among active, passive, and locative sentences, we further added effects of sentence type, hypothesizing that passives would elicit the most difficulty, followed by locatives, followed by actives. Third, we also added fixed effects of mode (lab vs. web) to assess whether different outcomes emerged from our data online compared to in the lab. Rather than modeling two separate regressions (one for in‐lab data and one for web data), this approach was preferred due to our theoretical interest in interactions: we included interactions between fixed effects as a way of evaluating whether specific groups or sentence types were more or less affected by web versus lab differences than others. Models were initially fitted with by‐item and by‐participant random slopes, but no models converged with these terms included; as such, separate by‐item and by‐participant random intercepts were used wherever models converged. Our analyses were therefore threefold:

1. *Accuracy*: PWA data only due to controls showing ceiling performance. Generalized binomial mixed‐effects regression with fixed effects of structure type (active, passive, locative) and mode (web, lab), and random effects of participant and trial.
2. *RTs* (square root‐transformed): linear mixed‐effects regression with fixed effects of structure type (active, passive, locative), mode (web, lab), and group (control, PWA), and random effects of participant and trial.
3. *Target eye gaze proportions*: generalized quasibinomial models, including fixed effects of structure type (active, passive, locative), mode (web, lab), and group (control, PWA). Random effects structures led to model convergence and singular fit issues, and so no random effects were included.

Additionally, where possible, we computed Bayesian mixed models and corresponding null models for each variable and each interaction of interest to confirm the presence of null effects. These full and null models were then compared statistically, yielding Bayes’ factors (BFs). A BF of > 3 favors the full model (suggesting the interaction of interest is a significant predictor of the observed variance), while BFs < 1/3 support the null model (with smaller values showing greater degrees of support; Van Doorn et al. 2021; Lee and Wagenmakers 2014). Our Supporting Information at https://osf.io/n6gu7/ contain the full code used for the analysis.

## 3 Results

### 3.1 Accuracy

**FIGURE 6:**
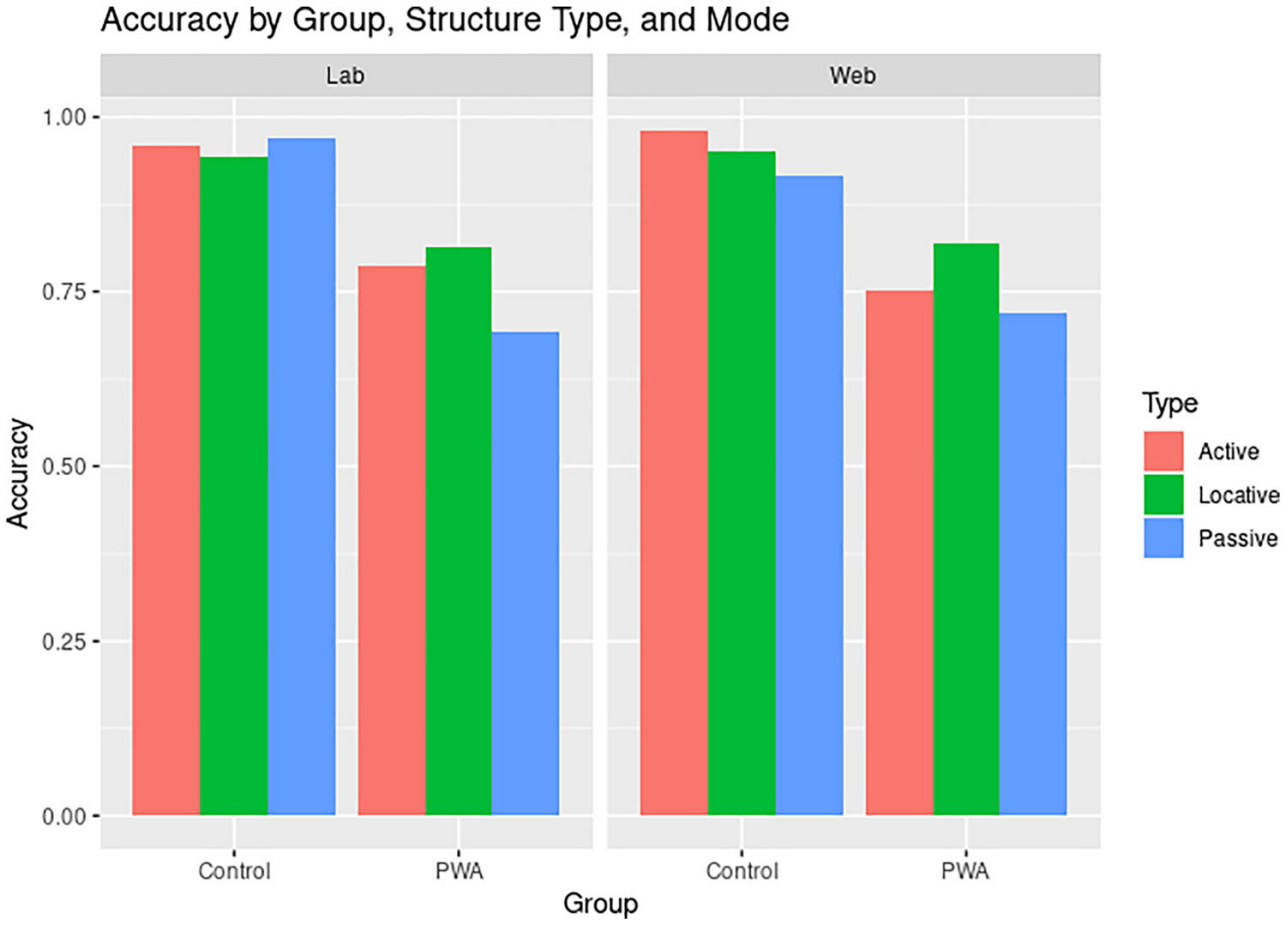
Plot showing average accuracy by group (control vs. PWA), structure type (active vs. passive vs. locative), and mode (lab vs. web). PWA showed lower accuracy than controls, especially on passives.

Accuracy models were run with PWA data only due to controls performing at ceiling on the task in both modalities (see Figure 6). Supporting Information Table A (https://osf.io/n6gu7/) gives a full summary of the model used to evaluate accuracy. This full model was no more accurate in explaining the data than a null model not including the interaction of structure type by mode, suggesting accuracy differences by structure type were similar in both the lab and web (*χ* ^2^ = 1.64, *p* > 0.05; BF_type × mode_ = 0.035). PWA were less accurate on passives compared to actives (*z *= −2.611 *p *< 0.01, *d *= −0.144; 97.5% CI [−1, −0.15]), but no other differences were observed (all *p*s > 0.05).

### 3.2 Reaction Times

**FIGURE 7:**
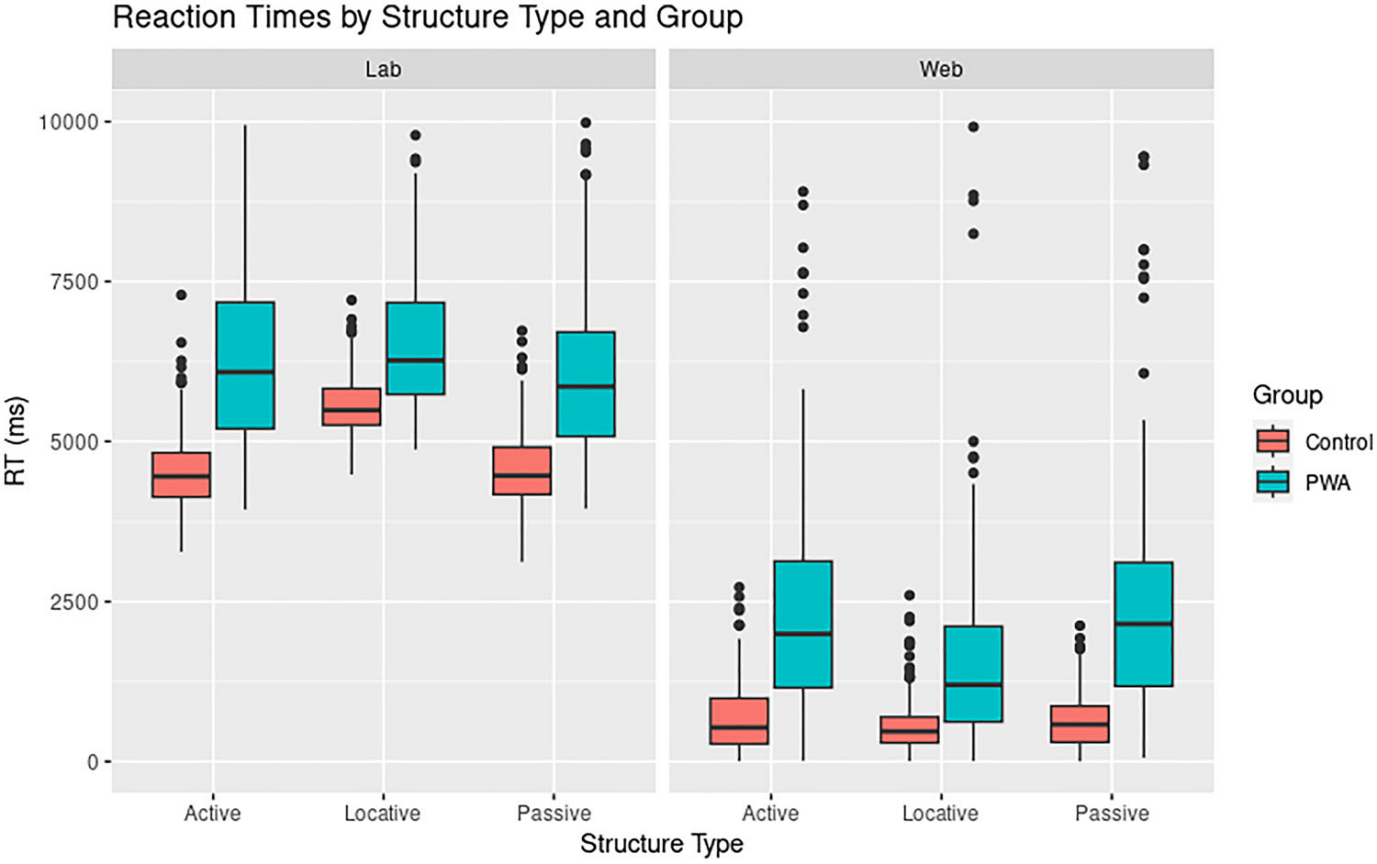
Boxplot showing reaction times (RTs) by structure type, mode of administration, and group. RTs were generally faster on the web compared to the lab, and PWA responded more slowly than controls.

RTs were square‐root transformed before being added to any model. RT models were run with random effects of trial and participant, and included fixed terms of group × structure type × mode. Figure 7 shows RTs by these parameters, and Table 3 summarizes full model output. PWA responded slower than controls overall (*t*(65) = 4.269, *p *< 0.001, *d *= 1.058; 97.5% CI [62, 72]) and were comparatively faster on locative compared to passive and dative trials (*t*(2275) = −4.913, *p *< 0.001, *d *= −0.206; 97.5% CI [−10, −4]). Importantly, RTs on the web were generally shorter than those in the lab (*t*(64) = −11.556, *p *< 0.001, *d *= −2.889; 97.5% CI [−50, −36]), and this difference was greatest for locatives (*t*(2264) = −6.190, *p *< 0.001, *d *= −0.260; 97.5% CI [−12, −6]; BF_group × type × mode_ > 1000). Both groups, in fact, responded slower to locative sentences in the lab compared to on the web, especially the control group (see Figure 7). However, the overall difference between modalities was larger in PWA than it was in controls (*t*(65) = 2.1, *p *< 0.05, *d *= 0.519; 97.55, 97.5% CI [−5,1]).

**TABLE 3:** Linear mixed‐effects model summary for square‐root transformed reaction time data.

Parameter	Est.	SE	*DF*	*t*	*p*	*d*	Lower CI	Upper CI
Intercept	67.288	2.649					62.186	72.389
Group: PWA	16.042	3.758	65	4.269	**< 0.001**	1.058	8.803	23.280
Structure type: locative	7.166	1.010	2272	7.098	**< 0.001**	0.298	5.190	9.142
Structure type: passive	0.265	0.988	2268	0.268	0.789	0.011	−1.167	2.200
Mode: web	−43.240	3.742	64	−11.556	**< 0.001**	−2.889	−50.447	−36.032
Group: PWA × structure type: locative	−7.380	1.502	2275	−4.913	**< 0.001**	−0.206	−10.321	−4.440
Group: PWA × structure type: passive	−1.614	1.511	2265	−1.068	0.286	−0.045	−4.579	1.346
Group: PWA × mode: web	11.172	5.321	65	2.100	**0.040**	0.519	0.922	21.422
Structure type: locative × mode: web	−8.822	1.425	2264	−6.190	**< 0.001**	−0.260	−11.612	−6.032
Structure type: passive × modality: web	−0.723	1.423	2261	−0.508	0.612	−0.021	−3.513	2.268
Group: PWA × structure type: locative × mode: web	−0.843	2.134	2271	−0.395	0.693	−0.017	−5.020	3.337
Group: PWA × structure type: passive × mode: web	4.109	2.180	2264	1.885	0.060	0.079	−0.170	8.393

*Note*: Model included random effects of participant (*σ *= 104.1, SD = 10.2) and trial (*σ *= 1.3, SD = 1.1). *R* ^2^_Marginal_ = 0.710; *R* ^2^_Conditional_ = 0.851. Bold values represent those meeting the significance threshold of p < .05.

Abbreviations: CI, confidence interval; DF, degrees of freedom; Est., estimate; SE, standard error (2.5% and 97.5%).

### 3.3 Eye Gaze Target Proportions

We aimed to investigate the quality of web versus lab‐based eye tracking in two dimensions: (1) sensitivity to detecting group differences (essential for clinical research) and (2) timing accuracy (given how critical the time course of sentence processing is to psycholinguistic research). Different analysis steps were taken for each dimension, which are detailed below.

#### 3.3.1 Group Differences

We hypothesized that PWA should show lower levels of target gaze proportions than controls overall, and especially on passives (and potentially locatives) than actives, given the processing difficulties PWA often exhibit with complex syntax. If webcam‐based eye tracking is as sensitive as lab‐based tracking, not only should overall group differences show in both modes, but these active/passive/locative differences should also be apparent. Due to the large number of model parameters and variations of model formulae (120 bins and interactions with group, type, and mode), full model summaries appear in our Supporting Information (https://osf.io/n6gu7/), and we will limit model descriptions here to critical terms. Figure 7 shows mean target gaze proportions per bin (relative to the bin in which disambiguation occurred) by group and mode.

**FIGURE 8:**
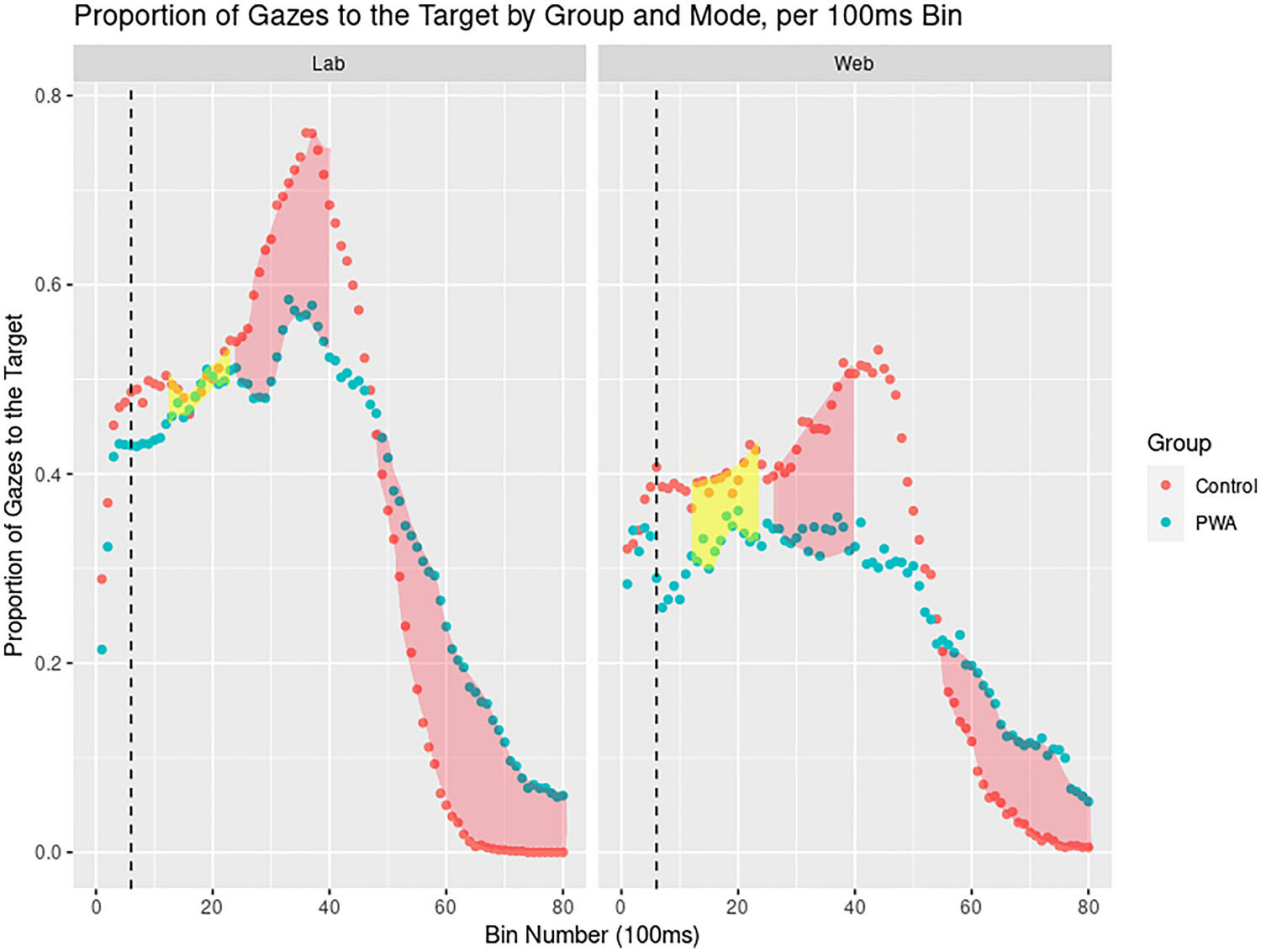
Plots of target gaze proportions by bin number (relative to the bin in which disambiguation occurred, as indicated by dotted lines), group, and mode, showing generally lower proportions of target gazes on the web compared to the lab, but similar overall differences between groups. Shaded areas in RED indicate where models showed differences between Controls and PWA; shaded areas in YELLOW indicate where this group difference was *larger* in the web compared to the lab.

A model predicting target gaze proportions by bin and mode (Supporting Information Model A; https://osf.io/n6gu7/) showed that proportions were lower than in the lab across the board, up until bin 6300–6400 (all *t*s < −1, *p*s < 0.05), when proportions became similar in both lab and web (*t*s [–1.977; 0.673], *p*s > 0.05). Bayesian Models confirmed these differences (BF_bin × mode_ > 1000). Including group as an additional interaction term (Supporting Information Model B; https://osf.io/n6gu7/) resulted in the finding that overall target gaze proportions were lower in PWA than in controls (*t* (215,501) = −4.043, *p *< 0.001, *d *= −0.024, 97.5% CI [−0.033; −0.016]). Overall, PWA diverged from controls from 1300–1400 ms onward *(t *= 2.139, *p *< 0.05) to 2600–2700 ms (*t *= 1.733, *p *= 0.08), and again from 3500–3600 ms (*t *= −2.847, *p *< 0.01) to 3800–3900 ms (*t *= −2.121, *p *< 0.05), and from 4500 ms (*t *= 3.391, *p *< 0.001) to trial end. As also seen in Figure 8, PWA looked less to the target in this ∼1300–3900 ms disambiguation window, but more than controls after ∼4500 ms. This group difference occurred in both lab and web tracking—indeed, group differences were *larger* on the web than the lab from 1200–1300 ms (*t *= −2.209, *p *< 0.05) to 2300–2400 ms (*t* = −2.695, *p* < 0.01), and from 4100–4200 ms (*t *= −3.213, *p *< 0.01) to 6900–7000 ms (*t *= −3.076, *p *< 0.05). Both tracking modes were therefore sensitive to detecting differences between groups overall.

**FIGURE 9:**
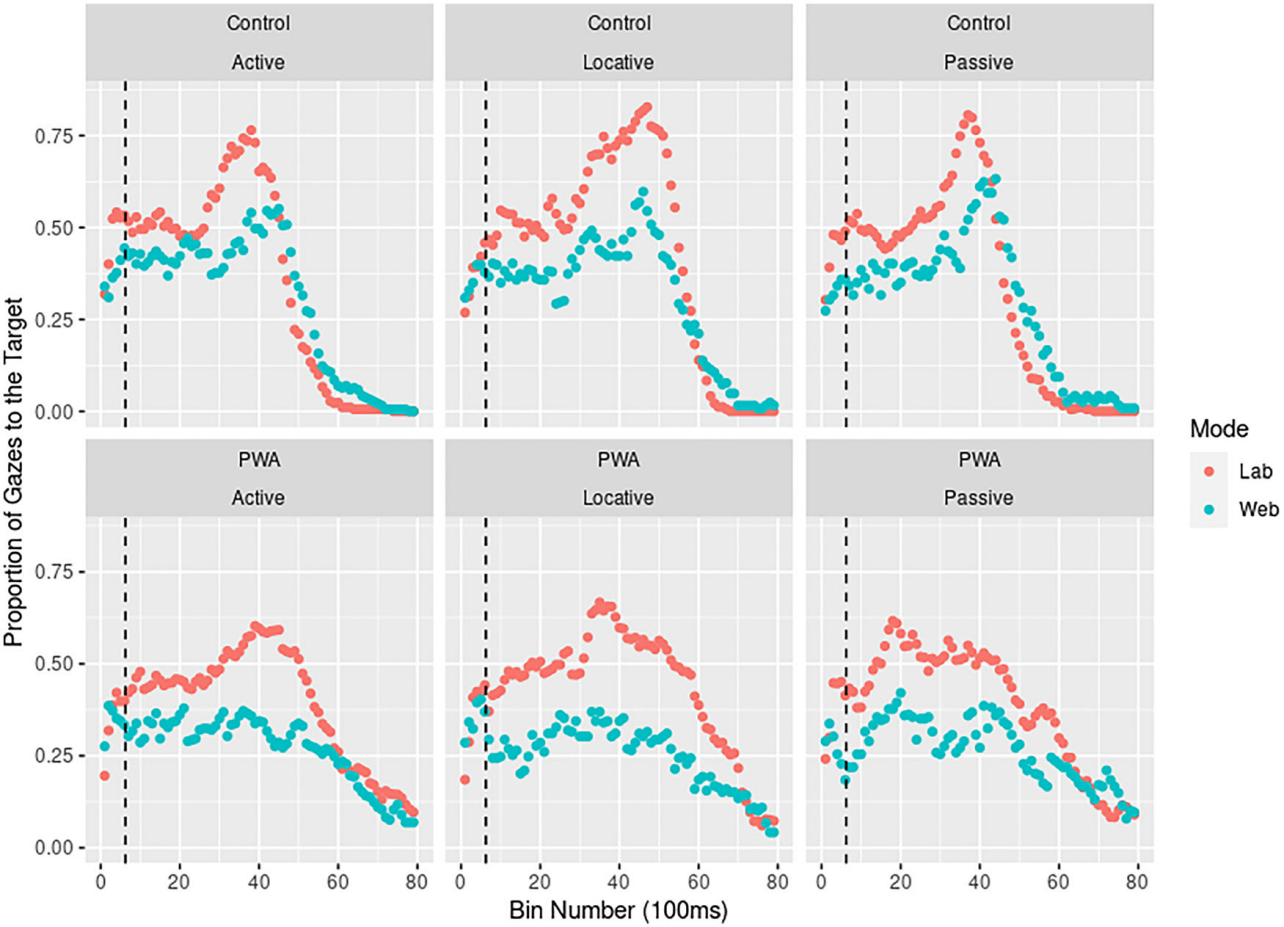
Plots of target gaze proportions by bin number (relative to the bin in which disambiguation occurred), structure type, mode, and group, with disambiguation points shown as dotted lines. This figure shows clear disambiguation effects in controls (as increases in target gaze proportions following disambiguation) but less clear effects in PWA, especially on the web.

However, we were also interested in detecting differences by structure type (see Figure 9). We therefore built a model, including bin × structure type × mode (Supporting Information Model C; https://osf.io/n6gu7/) to examine differences by structure type. Across groups, locatives elicited higher proportions of target gazes from 4300–4400 ms (*t *= 2.456, *p *< 0.05) to 6800–6900 ms post‐onset (*t *= 2.085, *p *< 0.05), indicating a longer, persisting disambiguation effect for locatives compared to actives. In similar time bins, locatives appeared to elicit *lower* proportions of target gazes on the web compared to the lab. This was the case from 4500–4600 ms (*t *= −2.470, *p *= 0.01) to 6100–6200 ms (*t *= −2.024, *p *< 0.05). Passives did not elicit lower proportions of target gazes than actives in this model, which included both participant groups, except from 5000–5100 ms (*t *= −2.002, *p *< 0.05) to 5200–5300 ms (*t *= −1.996, *p *< 0.05) and from 7200 to 7300 ms (*t *= −2.066, *p *< 0.05). Around these later bins, target gaze proportions were also higher in the web compared to the lab (6900–7000 to 7400–7500: *t*s [2.203; 3.848], *p*s < 0.05). In short, both modes appeared sensitive to locative differences, although this effect was smaller on the web compared to the lab, but no effects of passive syntax were evident.

Given the frequency and strength of past studies’ findings of passive processing in PWA (e.g., Meyer, Mack, and Thompson 2012; Mack, Nerantzini, and Thompson 2017; Van Boxtel et al. 2023), however, this lack of active/passive difference was surprising, and we therefore split off PWA data and modeled this separately. It could be the case that controls, for whom passives should not have presented a problem, therefore also showed no eye gaze differences between actives and passives. This PWA‐only model included a bin × structure type × mode interaction and is referred to as Supporting Information Model D (https://osf.io/n6gu7/). As in previous models, PWAs’ gaze proportions to the target rose from 100–200 ms (*t *= 2.813, *p *< 0.01) to 4900–5000 ms (*t *= 5.592, *p *< 0.001) and then decreased from 5500–5600 ms (*t *= −2.224, *p *< 0.05) to trial end (*t *= −6.988, *p *< 0.001). PWA consistently spent less time looking at the target on the web compared to the lab, from 300–400 ms (*t *= −2.045, *p *= < 0.05) to 5500–5600 ms (*t *= −2.487, *p *= 0.01). Results from locatives were less defined than in the all‐group model, with PWA showing only somewhat greater target gazes to locatives from 5700 to 5800 (*t *= 1.963, *p *< 0.05) and from 6000 to 6100 ms (*t *= 2.013, *p *< 0.05). This suggests the large effect of locatives causing more sustained target gaze proportions in the all‐group model was mainly driven by the control group. In PWA, passives led to lower target gaze proportions only in a narrow time window, from 4800–4900 ms (*t *= −2.165, *p *< 0.05) to 5100–5200 ms (*t *= −2.044, *p *< 0.05). There were some indications that locatives resulted in lower target gaze proportions on the web specifically (5800–5900 ms: *t *= −2.331, *p *< 0.05), and passives showed a similarly weak interaction with mode, such that gaze proportions to passives on the web were higher from 7100–7200 ms (*t *= 2.732, *p *< 0.01) to 7300–7400 ms (*t *= 2.078, *p *< 0.05).

#### 3.3.2 Temporal Accuracy

To assess temporal accuracy, we computed an average difference measure between PWA and controls in each time bin, for each type, in both lab and web. For instance, PWAs’ mean target gaze proportion for active trials in the 300–400 ms bin was subtracted from controls’ mean, and this difference (hereafter referred to as Δ*gaze*) was used as dependent variable to assess timing accuracy by using bin as a predictor. This allowed for the isolation of temporal differences by group, type, and mode. The use of difference measures is a common tactic in some sub‐types of quantitative research, including event‐related potential studies (e.g., Gaillard 1988; Kappenman et al. 2021), and although these measures have their limitations, especially when related to individual difference scores (see Meyer et al. 2017), they are nevertheless a useful tool to isolate parameters of interest when various parameters interact. Following a univariate fit analysis using the *fitdistrplus* library (Delignette‐Muller and Dutang 2015; see the Supporting Information at https://osf.io/n6gu7/), Δ*gaze* was modeled using linear regression and (given that Δ*gaze* constitutes an average) without random effects, across structure types. This should be kept in mind when interpreting effect sizes and significance.

**FIGURE 10:**
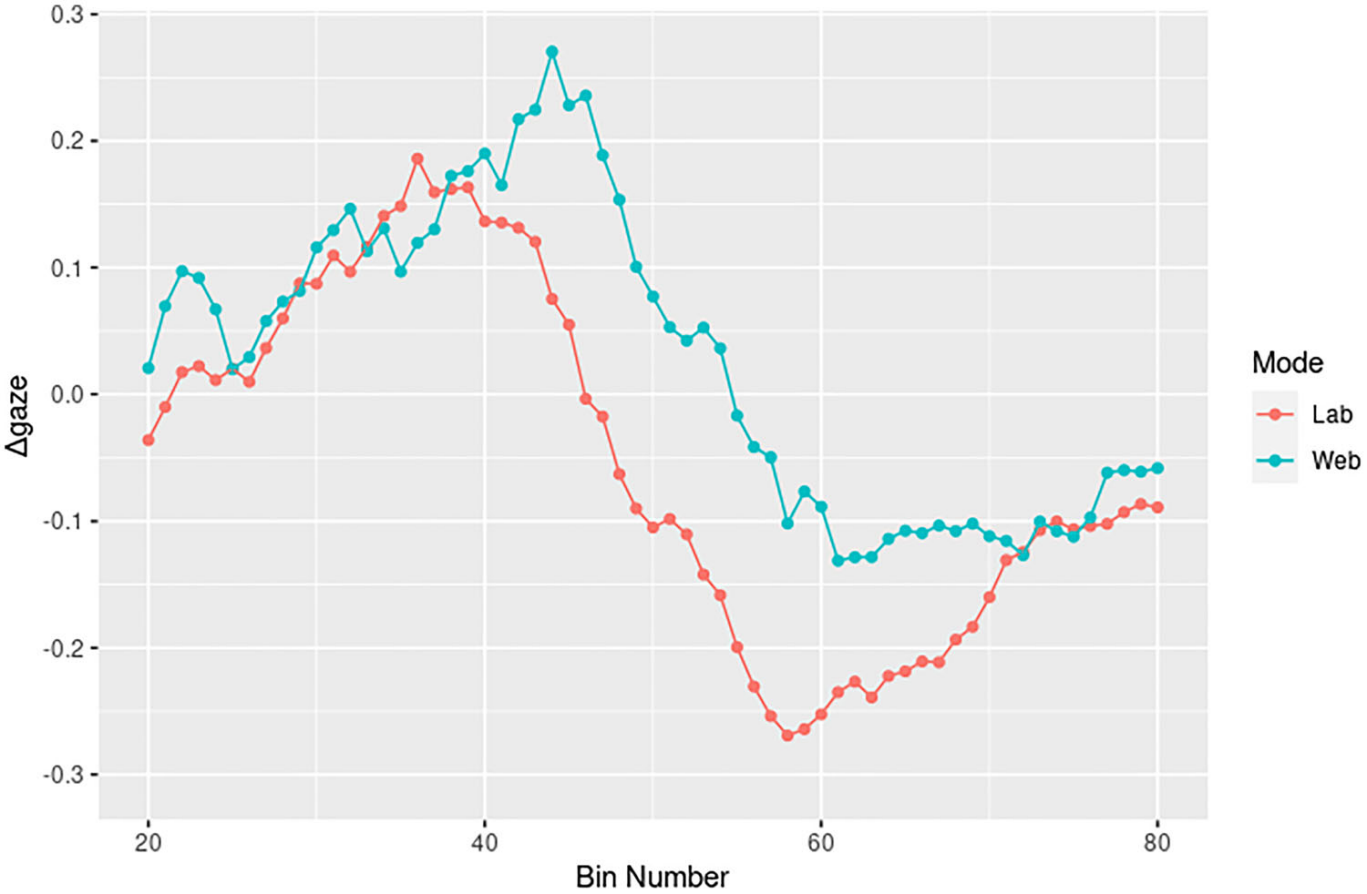
Plot of mean group differences (*M*_control_–*M*_PWA_) by bin number and mode, showing delayed recording in the web compared to the lab of approximately 400–800 ms (4–8 bins).

Differences between PWA and controls became larger as trials progressed (*t*(4) = −6.065, *p *< 0.001, *d *= −4.012, 97.5% CI [−0.009; −0.005]). Crucially, Δ*gaze* remained higher later on in trials on the web, evidenced by a bin number × web interaction (*t*(4) = 2.793, *p *< 0.01, *d *= 0.626, 97.5% CI [0.001, 0.008]). This is suggestive of temporal delays in the web compared to the lab. Indeed, Figure 10 shows the persistence of this delay. Δ*gaze* on the web peaked around bin 45 (4500–4600 ms post‐onset), whereas lab differences peaked around 3800 ms post‐onset. Similarly, Δ*gaze* (web) reached its lowest value at 6100–6200 ms, but Δ*gaze* (lab) dipped approximately 400 ms earlier, in the 5700–5800 ms bin. For full model output, refer to Supporting Information Table B (https://osf.io/n6gu7/).

## 4 Discussion

This study reports a comparison of in‐lab and web‐based eye tracking in a group of patients with aphasia and healthy controls. Both groups participated in the same web‐based and in‐lab sentence‐picture matching task, with task order alternating. Eye movements were recorded using WebGazer.js and Gorilla.sc on the web, and using an EyeLink remote infrared tracker in the lab. We asked whether both modalities are sensitive to differences between groups and sensitive to differences between processing of actives, passives, and locatives. This study is the first to compare web‐ and lab‐based eye tracking in a language‐impaired group and, to our knowledge, only the second to conduct web‐based eye tracking with clinical populations in general (following Greenaway et al. 2024, with dementia patients).

This study found that both in‐lab and web‐based eye tracking are sensitive to differences between patients with aphasia (PWA) and healthy controls, though possibly not to the same extent and with important caveats in mind. Both modalities accurately reflected group differences on raw data and when difference scores were computed (see Figure 10). Accuracy scores were unaffected by modality, and although PWA generally took longer to respond on the web compared to the lab, response times were still comparable across modalities. Finally, and most crucially, web‐based tracking recorded group differences between PWA and controls, despite the much lower sampling rate of webcam‐based tracking (∼14 Hz compared to ∼430 Hz in the lab). Our results add to the rapidly growing body of literature reporting successful application of common eye‐tracking experiments on the web: Slim and Hartsuiker (2023) and Greenaway et al. (2024) found similar results on a web‐based visual world task and an emotional judgment task, respectively, compared to previous in‐lab results; Hutt et al. (2024) validated the accuracy of web‐based eye tracking for narrative prediction tasks, and Özsoy et al. (2023) successfully used a morphological anticipation design. This study adds two‐choice sentence‐picture matching tasks to possible paradigms to use with web‐based eye tracking. Perhaps more crucially, however, this study also demonstrates that web‐based recording of eye movements in a *clinical sample* is possible in the first place. Greenaway et al. (2024) made the first important step in applying web‐based eye tracking to clinical groups, showing successful WebGazer performance in patients with dementia by measuring emotional biases to attention to static visual stimuli. The current study expands these findings significantly: Greenaway et al. included only a clinical group (and no control group) and studied only static images, without assessing gaze time to different pictures presented on screen at the same time. The present study was the first to evidence complex linguistic processing in a clinical population, namely, aphasia, and an age‐matched control group, on screens populated with large amounts of information.

We demonstrate that web‐based eye data recording of patient data is not only possible, but also necessary: Some of the most frequently cited studies of sentence processing in aphasia included problematically low participant numbers. For instance, Swinney, Zurif, and Nicol (1989) worked with eight patients, whereas Berndt et al. (1997) included 10, and Peelle et al. (2007) studied 13 patients with progressive aphasia. More recent studies similarly use small group samples to generalize to wider populations (e.g., *n* ∼ 20 in Barbieri et al. 2021; *n *= 19 in Chapman and Hallowell 2021, *n *= 16 in Van Boxtel et al. 2023; and *n *= 16 in the present study). This is not entirely surprising, and we certainly do not intend to criticize any of the authors we cite. Clinical populations are often difficult to recruit due to relatively low incidence in the community, and traveling to a laboratory for research testing can be difficult for patients due to their limited physical mobility (e.g., hemiparesis following stroke) or access to other means of transportation. These constraints contribute to difficulty recruiting a large and diverse participant sample, limiting generalizability and statistical power. The lack of sample diversity and low generalizability to populations in clinical research can be, in part, ameliorated by conducting experiments on the internet. Participants are not required to be physically present in a laboratory and can be drawn from diverse backgrounds, whether socio‐economic, cultural, ethnic, or racial. In this study, we have shown how a reliable, sub‐behavioral method can be adapted for widespread web‐based use with clinical populations.

In studies of aphasia specifically, eye tracking is a critical tool to uncover not only the cognitive‐linguistic foundations of aphasia symptoms, but also to gage treatment efficacy. Eye‐tracking studies in PWA have yielded crucial findings on the nature of language impairments in aphasia, including sentence comprehension (Sharma et al. 2021; Mack, Ji, and Thompson 2013), word recognition (Mirman et al. 2011), sentence planning (Cho and Thompson 2010; Lee and Thompson 2011), but eye movements have also been studied as an index of successful implicit learning (Van Boxtel et al. 2023) and treatment outcomes (Mack, Nerantzini, and Thompson 2017). The paradigms often used in these studies include sentence‐picture matching, visual world, and picture description tasks, all of which could be easily adapted to an online format and could therefore be administered to a much wider experimental audience, not only raising the generalizability and validity of aphasia research (and, hopefully, uncover more effective treatments), but also involving a wider part of the aphasia community worldwide.

There are nevertheless a number of significant caveats to consider when recording patients’ eye data with WebGazer, some of which are implied by our present findings. Despite the overall success of web‐based tracking with aphasia patients in this study, differences by structure type (active, passive, locative) were more variable. Specifically, we hypothesized that PWA should show lower proportions of target gazes on passive compared to active (and potentially locative) trials. However, these effects were not captured well by web‐based tracking in PWA (see Figure 8), who showed lower target gaze proportions on passives compared to actives only in a narrow window approximately 5000 ms post‐onset. Additionally, gazes to the target on passive trials were higher on the web than in the lab, suggesting web‐based tracking was less sensitive to PWAs’ processing difficulties on passives (as indicated by their lower accuracy scores on passives compared to actives). This less uniform, more variable pattern is characteristic of clinical groups, who generally show larger intra‐sample variability, especially with low sample sizes (e.g., Well, Pollatsek, and Boyce 1990). Further, a potential limitation of this study (and one that should be carefully thought out in future validation efforts) is that our control and PWA samples were not balanced for gender. This may have affected the strength of differences between groups (see, e.g., Glenberg et al. 2009; Logan and Johnston 2010).

One possibility for examining whether group differences in future studies are comparable between lab and web tracking is to apply specialized equivalence testing protocols to eye gaze data. We specifically regard equivalence testing practices in (clinical) psychology as potentially fruitful in this regard. Such testing would not only shed light on whether timing is equivalent between the web and lab modalities (which we assessed using a difference measure; see below) but could further evaluate whether the strength of eye gaze modulations was equal in either PWA and controls between the web and the lab (see Lakens, Scheel, and Isager 2018, for a tutorial). Although we did not include such an analysis in this manuscript (as the present manuscript was a highly explorative study), these sorts of approaches are highly promising for future research comparing web and lab tracking.

Our computation of a difference measure between groups (Δ*gaze*) showed that timing is consistently delayed on the web compared to the lab. Specifically, group differences peaked around 3500 ms post‐onset in the lab, but not until around 4300 ms on the web. Δ*gaze* reached its lowest point around 5700 ms (lab) and 6200 ms (web), respectively (see Figure 10), resulting in a delay of between 500 and 800 ms on the web compared to the lab. With this finding, we mirror previous studies’ reports of temporal delays in web‐based eye tracking (Slim and Hartsuiker 2023; Semmelmann and Weigelt 2018). However, although Slim and Hartsuiker (2023) found a consistent, across‐the‐board delay of 300 ms in their college‐aged participants, the temporal lag between modalities in our participants who are older and have aphasia appears somewhat more variable and greater than 300 ms. Thus, this difference could be due to a combination of different stimuli and study participant groups examined between the studies. The types of grammatical structures the current experiment used were more complex than those in Slim and Hartsuiker (2023). This could have resulted in additional timing delays and variability in the data. Second, the lower sample size of our study compared to Slim and Hartsuiker's sample (*n *= 32 vs. *n *= 57, respectively) and the inclusion of older adults and patients with aphasia in our study could have contributed to greater timing variability. Alternatively, Slim and Hartsuiker conducted their study using PC Ibex (Zehr and Schwarz 2018), although we used Gorilla.sc, and such the front‐end software used to run both studies was entirely different. This will be an important topic for future studies: Given the different front‐ends used in web‐based eye‐tracking studies to date, the reliability and accuracy of each should be carefully examined. Additionally, we compared lab and web tracking in the same sample of participants in the same task, whereas Slim and Hartsuiker compared their results to patterns obtained in earlier studies. This could have obscured or changed the magnitude of timing differences. The magnitude of temporal lag may also have been greater in our study due to our participants staying on a videoconferencing call with the experimenter during participation, which could have taxed internet speeds more than in Slim and Hartsuiker (2023). Whatever the causes, however, it is clear web‐based eye tracking does not offer the same temporal precision as in‐lab eye tracking currently does.

Indeed, even in the behavioral patterns we observed, there were some temporal differences between the lab and web modalities. RTs were generally faster online than in the lab, but this difference was smaller for PWA. This could be due to different RT computations between the two software systems we used, but, as one of the reviewers of this manuscript pointed out, there may also be variations in “commitment” to the task between modalities. Some findings have indeed found small behavioral differences between in‐person and online behaviors (e.g., Bridges et al. 2020). However, most evidence from behavioral tasks supports confidence in online methods, claiming these differences are small and almost entirely non‐significant (Buso et al. 2021; Anwyl‐Irvine et al. 2021). We further attempted to minimize issues of commitment to the task, attentional or otherwise, by guiding the participant through the study on a videocall. It may be of interest for future research to compare the efficacy of online research without such videocall guidance (for instance, by recruiting participants through an online platform such as Mturk or Prolific.co) to our method, and to in‐person performance, to evaluate these differences further.

Generally, proportions of gazes to the target picture were lower in the web compared to the lab, and this was especially true for PWA. Just as PWA shows greater inter‐participant variability than controls in most experimental paradigms (see, e.g., Van Boxtel et al. 2023; Keen and Lee 2024), it is likely the case that online eye tracking shows greater variability across participants and sessions as well. This variability may have been exacerbated by the lower sampling rate of WebGazer compared to in‐lab tracking and may have stacked with participant variability in PWA to produce the lower overall target gaze proportions found in our data. In future studies, problems arising from such variability could be ameliorated through direct equivalence‐based comparisons for each group between lab and web, as discussed above.

Temporal delays combined with potential spatial precision reductions and greater sampling variability could cause issues when attempting to conduct more fine‐grained research with web‐based eye tracking, such as reading studies or picture description paradigms. Careful examinations of what circumstances ameliorate temporal and spatial inaccuracies could result in specific recommendations for (clinical) researchers using WebGazer in their studies. For instance, lighting conditions play an important role in successful calibration and validation using WebGazer: If the algorithm cannot accurately detect pixels corresponding to a participant's eyes due to glare or darkness, accuracy could be lowered. We generally found that all participants could wear glasses during the experiment, but we cannot say with certainty how this affected our findings. Background lighting, internet bandwidth, and webcam frames per second would all have affected how well WebGazer estimated participants’ gaze positions, and it will be crucial for future studies to validate what the optimal conditions for recording are. Further, however, we did not specifically investigate what impact participants’ ethnicity, race, and associated skin color had on accuracy of recording. Previous eye‐tracking studies have suggested that in‐lab trackers are less accurate for some ethnic groups than for others (Blignaut and Wium 2014), which presents a crucial methodological defect. However, all evidence from webcam‐based tracking suggests the accuracy of online eye tracking is unaffected by participants’ ethnicity (see, e.g., Elahi et al. 2013; Werchan, Thomason, and Brito 2023). We therefore view online tracking as a remedy for, rather than an exacerbation of, this crucial issue. A systematic investigation of ethnic and racial differences in accuracy and precision of webcam‐based recording would nevertheless alleviate these concerns further.

In general, however, we have a number of recommendations for future webcam‐based studies using clinical populations that could improve the reliability of future findings if taken into account: First, we recommend studies calibrate and recalibrate frequently. Second, stimuli should be spaced far enough apart to account for potential timing and precision inaccuracies (see Figures 1 and 5 for examples of our trial display), but relying entirely on stimuli presented in the far corners of the screen may not be recommended (Bogdan et al. 2024). Third, efforts should be made to increase participants’ internet speeds and bandwidth. This could include closing other applications, not being on videoconferencing calls with participants (as we did in this study), or requiring a wired connection. The exact influence of internet speed on WebGazer's tracking should be specifically examined. Fourth, the analysis process for webcam‐based data is cumbersome compared to in‐lab EyeLink data, and standardized preprocessing pipelines should be developed that could help set a benchmark for future studies (for the pipeline used in this study, consult the Supporting Information at https://osf.io/n6gu7/).

To conclude, web‐based eye tracking through participants’ own webcams has tangible potential for real transformative impact on fields that include patient samples, by increasing sample sizes and including participants with more diverse backgrounds. We successfully discovered group differences between patients with aphasia and healthy controls on webcam‐based eye tracking, and although some conditional differences and timing accuracy were not as refined as in‐lab‐based tracking, we suggest webcam eye tracking has great potential for several fields. We generally replicated previous studies that used WebGazer and other web‐based eye trackers but provided novel evidence of the algorithm's reliability by directly comparing in‐lab and web‐based performance in the same participants, including a clinical group. We recommend future studies investigate the temporal and spatial precision of webcam‐based eye tracking more carefully and specifically and consider participants’ internet speeds and the data preprocessing stage for web‐based eye data. In general, we predict webcam eye tracking will become a widely used method with clinical and non‐clinical populations and will make significant contributions to our understanding of language processing and more.

## Author Contributions

**Willem S. van Boxtel**: Conceptualization, investigation, writing—original draft, methodology, validation, visualization, software, formal analysis, project administration, data curation. **Michael Linge**: Investigation, writing—original draft, data curation. **Rylee Manning**: Investigation, data curation, project administration. **Lily N. Haven**: Investigation, project administration, data curation. **Jiyeon Lee**: Funding acquisition, writing—review and editing, resources, supervision.

## Ethics Statement

Research reported in this manuscript received ethical approval from the local Institutional Review Board.

## Consent

All participants provided informed consent to participate and were aware their data would be used to prepare publications.

## Conflicts of Interest

The authors declare no conflicts of interest.

### Peer Review

The peer review history for this article is available at https://publons.com/publon/10.1002/brb3.70112.

## Supporting information



Supporting Information

Supporting Information

Supporting Information

Supporting Information

## Data Availability

Supporting Information for this research are available from https://osf.io/n6gu7/, and this study was not preregistered.
