# Supplementary material for: Online Eye Tracking for Aphasia: A Feasibility Study Comparing Web and Lab Tracking and Implications for Clinical Use
Source: Brain Behav. 2024 Oct 29;14(11):e70112. doi: 10.1002/brb3.70112 (PMC11519703; doi:10.1002/brb3.70112)
Supplement: Supplementary file 1 — Supporting Information [file BRB3-14-e70112-s001.pdf]

Supplementary Model Output

Online eye-tracking for communication disorders:
a feasibility study comparing web and lab tracking

Model A

Generalized binomial mixed-effects model summary for accuracy data (PWA only):
*Accuracy ∼ Structure Type * Mode.*
Model included random effects of Participant (σ = 1.034, SD = 1.02) and Trial (σ = .085, SD = .291).
$R^2_{Marginal}$ = .021; $R^2_{Conditional}$ = .270. Est. = estimate; SE = standard error; DF = degrees of freedom.

Parameter	Est.	SE	DF	*z*	*p*	*d*
Intercept	1.622	.314				
Structure Type: Locative	.177	.253	1380	.728	.467	.036
Structure Type: Passive	-.594	.228	1378	-2.611	.009	-.144
Mode: Web	-.286	.435	49	-.657	.511	-.177
Structure Type: Locative * Mode: Web	.315	.345	1381	.914	.361	.049
Structure Type: Passive * Mode: Web	.400	.312	1377	1.244	.214	.067

Model B

Linear model summary for Δgaze (difference of mean PWA and control target gaze proportion in each bin):
*Δgaze ∼ Bin * Mode.*
R^2 = .364. Est. = estimate; SE = standard error; DF = degrees of freedom.

Parameter	Est.	SE	DF	*t*	*p*	*d*
Intercept	.313	.048				
Bin Number	-.007	.001	4	-6.065	< .001	-4.012
Web	-.126	.067	78	-1.874	.065	-.293
Bin Number * Web	.005	.002	4	2.793	.007	.626

Model C

*Proportion of Target Gazes ∼ Bin * Mode.* Est = Estimate; SE = Standard Error; OR = odds ratio;

Parameter	Est.	SE	*z*	*p*	*OR*
Intercept	-1.045	.057			
(100,200]	.438	.078	5.620	< .001	1.549
(1000,1100]	.912	.076	11.938	< .001	2.489
(1100,1200]	.960	.076	12.572	< .001	2.611
(1200,1300]	.952	.076	12.471	< .001	2.592
(1300,1400]	.964	.076	12.629	< .001	2.623
(1400,1500]	.918	.076	12.020	< .001	2.505
(1500,1600]	.903	.076	11.813	< .001	2.466
(1600,1700]	.966	.076	12.656	< .001	2.628
(1700,1800]	1.008	.0763	13.206	< .001	2.740
(1800,1900]	1.069	.076	14.009	< .001	2.913
(1900,2000]	1.049	.076	13.747	< .001	2.855
(200,300]	.805	.077	10.508	< .001	2.236
(2000,2100]	1.066	.076	13.972	< .001	2.905
(2100,2200]	1.110	.076	14.533	< .001	3.033
(2200,2300]	1.158	.076	15.156	< .001	3.182
(2300,2400]	1.157	.076	15.145	< .001	3.179
(2400,2500]	1.137	.076	14.896	< .001	3.119
(2500,2600]	1.158	.076	15.164	< .001	3.184
(2600,2700]	1.205	.076	15.763	< .001	3.336
(2700,2800]	1.258	.077	16.440	< .001	3.518
(2800,2900]	1.320	.077	17.097	< .001	3.706
(2900,3000]	1.365	.077	17.781	< .001	3.915
(300,400]	.871	.076	11.399	< .001	2.390

(3000,3100]	1.498	.077	19.402	< .001	4.471
(3100,3200]	1.574	.078	20.306	< .001	4.825
(3200,3300]	1.664	.078	21.345	< .001	5.279
(3300,3400]	1.678	.078	21.510	< .001	5.356
(3400,3500]	1.690	.078	21.638	< .001	5.417
(3500,3600]	1.744	.078	22.245	< .001	5.721
(3600,3700]	1.750	.078	22.307	< .001	5.754
(3700,3800]	1.666	.078	21.372	< .001	5.292
(3800,3900]	1.580	.078	20.379	< .001	4.854
(3900,4000]	1.478	.078	19.169	< .001	4.385
(400,500]	0.880	.076	11.510	< .001	2.410
(4000,4100]	1.437	.077	18.668	< .001	4.207
(4100,4200]	1.355	.077	17.657	< .001	3.876
(4200,4300]	1.334	.077	17.404	< .001	3.798
(4300,4400]	1.256	.077	16.420	< .001	3.512
(4400,4500]	1.280	.076	15.804	< .001	3.347
(4500,4600]	1.074	.076	14.076	< .001	2.928
(4600,4700]	.978	.076	12.805	< .001	2.658
(4700,4800]	.857	.076	11.213	< .001	2.357
(4800,4900]	.730	.077	9.515	< .001	2.074
(4900,5000]	.615	.077	7.970	< .001	1.849
(500,600]	.903	.076	11.815	< .001	2.466
(5000,5100]	.481	.078	6.197	< .001	1.618
(5100,5200]	.373	.078	4.773	< .001	1.452
(5200,5300]	.195	.079	2.454	.014	1.215
(5300,5400]	.117	.080	1.456	.145	1.124
(5400,5500]	-.004	.081	-0.052	.959	.996
(5500,5600]	-.134	.083	-1.621	.105	.875
(5600,5700]	-.247	.084	-2.946	.003	.781
(5700,5800]	-.322	.085	-3.798	< .001	.724
(5800,5900]	-.498	.088	-5.688	< .001	.608
(5900,6000]	-.637	.090	-7.081	< .001	.529
(600,700]	.923	.076	12.084	< .001	2.517
(6000,6100]	-.776	.093	-8.382	< .001	.460
(6100,6200]	-.888	.095	-9.348	< .001	.411
(6200,6300]	-1.018	.098	-10.374	< .001	.364
(6300,6400]	-1.154	.102	-11.349	< .001	.314
(6400,6500]	-1.222	.104	-11.795	< .001	.295
(6500,6600]	-1.272	.1056	-12.100	< .001	.280
(6600,6700]	-1.297	.106	-12.252	< .001	.274
(6700,6800]	-1.404	.109	-12.843	< .001	.246
(6800,6900]	-1.485	.112	-13.249	< .001	.226
(6900,7000]	-1.593	.116	-13.728	< .001	.203
(700,800]	.892	.076	11.668	< .001	2.439
(7000,7100]	-1.785	.124	-14.416	< .001	.168
(7100,7200]	-1.858	.127	-14.625	< .001	.156
(7200,7300]	-1.965	.132	-14.880	< .001	.140
(7300,7400]	-2.123	.140	-15.152	< .001	.120
(7400,7500]	-2.041	.136	-15.025	< .001	.130
(7500,7600]	-2.091	.138	-15.106	< .001	.121
(7600,7700]	-2.114	.140	-15.139	< .001	.117
(7700,7800]	-2.148	.141	-15.185	< .001	.112
(7800,7900]	-2.188	.144	-15.231	< .001	2.525
(800,900]	.926	.076	12.127	< .001	2.504
(900,1000]	.918	.076	12.015	< .001	2.503
Web	.203	.085653	2.372	.018 1.225	
(100,200] * Web	-.296	.118117	-2.508	.012	.744
(1000,1100] * Web	-.754	.117	-6.438	< .001	.470
(1100,1200] * Web	-.801	.117	-6.848	< .001	.449
(1200,1300] * Web	-.726	.117	-6.224	< .001	.484
(1300,1400] * Web	-.683	.116	-5.867	< .001	.505
(1400,1500] * Web	-.719	.117	-6.154	< .001	.487
(1500,1600] * Web	-.659	.117	-5.653	< .001	.517

(1600,1700] * Web	-.685	.116	-5.885	< .001	.504
(1700,1800] * Web	-.665	.116	-5.728	< .001	.514
(1800,1900] * Web	-.805	.116	-6.912	< .001	.447
(1900,2000] * Web	-.720	.116	-6.192	< .001	.487
(200,300] * Web	-.681	.117	-5.801	< .001	.506
(2000,2100] * Web	-.737	.116	-6.340	< .001	.479
(2100,2200] * Web	-.764	.116	-6.575	< .001	.466
(2200,2300] * Web	-.819	.116	-7.044	< .001	.441
(2300,2400] * Web	-.879	.116	-7.551	< .001	.415
(2400,2500] * Web	-.858	.116	-7.371	< .001	.424
(2500,2600] * Web	-.875	.116	-7.517	< .001	.417
(2600,2700] * Web	-.896	.116	-7.696	< .001	.408
(2700,2800] * Web	-.984	.117	-8.444	< .001	.343
(2800,2900] * Web	-1.024	.117	-8.787	< .001	.314
(2900,3000] * Web	-1.013	.116	-8.701	< .001	.290
(300,400] * Web	-.621	.117	-5.321	< .001	.272
(3000,3100] * Web	-1.071	.116	-9.194	< .001	.282
(3100,3200] * Web	-1.160	.117	-9.937	< .001	.313
(3200,3300] * Web	-1.239	.117	-10.591	< .001	.296
(3300,3400] * Web	-1.301	.117	-11.107	< .001	.330
(3400,3500] * Web	-1.266	.117	-10.819	< .001	.341
(3500,3600] * Web	-1.274	.117	-10.876	< .001	.280
(3600,3700] * Web	-1.217	.117	-10.401	< .001	.296
(3700,3800] * Web	-1.108	.117	-9.503	< .001	.296
(3800,3900] * Web	-1.077	.116	-9.251	< .001	.330
(3900,4000] * Web	-.968	.116	-8.331	< .001	..380
(400,500] * Web	-.625	.117	-5.361	< .001	.535
(4000,4100] * Web	-.881	.116	-7.594	< .001	.415
(4100,4200] * Web	-.886	.116	-7.639	< .001	.412
(4200,4300] * Web	-.866	.116	-7.471	< .001	.420
(4300,4400] * Web	-.743	.116	-6.423	< .001	.475
(4400,4500] * Web	-.702	.116	-6.064	< .001	.496
Bin(4500,4600] * Web	-.608	.116	-5.254	< .001	.544
Bin(4600,4700] * Web	-.554	.116	-4.782	< .001	.574
Bin(4700,4800] * Web	-.520	.116	-4.475	< .001	.594
Bin(4800,4900] * Web	-.530	.117	-4.525	< .001	.589
Bin(4900,5000] * Web	-.476	.118	-4.043	< .001	.621
Bin(500,600] * Web	-.691	.117	-5.914	< .001	.501
Bin(5000,5100] * Web	-.469	.119	-3.949	< .001	.626
Bin(5100,5200] * Web	-.518	.120	-4.309	< .001	.596
Bin(5200,5300] * Web	-.360	.121	-2.967	.003	.698
Bin(5300,5400] * Web	-.479	.124	-3.881	< .001	.612
Bin(5400,5500] * Web	-.452	.125	-3.606	< .001	.735
Bin(5500,5600] * Web	-.473	.128	-3.697	< .001	.799
Bin(5600,5700] * Web	-.416	.130	-3.211	.001	.659
Bin(5700,5800] * Web	-.369	.131	-2.821	.005	.692
Bin(5800,5900] * Web	-.308	.134	-2.295	.022	.735
Bin(5900,6000] * Web	-.225	.137	-1.645	.010	.799
Bin(600,700] * Web	-.830	.117	-7.066	< .001	.436
Bin(6000,6100] * Web	-.248	.141	-1.753	.080	.781
Bin(6100,6200] * Web	-.270	.146	-1.852	.064	.781
Bin(6200,6300] * Web	-.253	.150	-1.684	.092	.764
Bin(6300,6400] * Web	-.162	.153	-1.056	.291	.777
Bin(6400,6500] * Web	-.259	.159	-1.629	.103	.850
Bin(6500,6600] * Web	-.352	.164	-2.144	.032	.772
Bin(6600,6700] * Web	-.314	.164	-1.914	.056	.704
Bin(6700,6800] * Web	-.347	.171	-2.032	.042	.730
Bin(6800,6900] * Web	-.307	.174	-1.763	.078	.707
Bin(6900,7000] * Web	-.234	.178	-1.318	.188	.791
Bin(700,800] * Web	-.792	.117	-6.749	< .001	.453
Bin(7000,7100] * Web	-.108	.185	-.585	.558	.897
Bin(7100,7200] * Web	-.020	.187	-.106	.915	.980
Bin(7200,7300] * Web	-.031	.194	-.161	.872	.969

Bin(7300,7400] * Web	.134	.200	.673	.501	1.144
Bin(7400,7500] * Web	.010	.198	.052	.958	1.010
Bin(7500,7600] * Web	-.020	.203	-.099	.921	.980
Bin(7600,7700] * Web	-.367	.221	-1.660	.097	.693
Bin(7700,7800] * Web	-.399	.226	-1.769	.077	.671
Bin(7800,7900] * Web	-.460	.233	-1.977	.048	.631
Bin(800,900] * Web	-.774	.117	-6.608	$< .001$	.461
Bin(900,1000] * Web	-.810	.117	-6.908	$< .001$	.445
