# Supplementary material for: Online Eye Tracking for Aphasia: A Feasibility Study Comparing Web and Lab Tracking and Implications for Clinical Use
Source: Brain Behav. 2024 Oct 29;14(11):e70112. doi: 10.1002/brb3.70112 (PMC11519703; doi:10.1002/brb3.70112)
Supplement: Supplementary file 2 — Supporting Information [file BRB3-14-e70112-s002.pdf]

Full model summaries

Online eye-tracking for the clinical behavioral sciences: a feasibility study comparing web and lab tracking

Model A

*Target Proportions ~ Bin * Mode* (quasiomial family). Model R^2 = .218. Reference level for Bin was (0,100], reference level for Mode was Lab.

	Est	SE	*t*	*p*	Lower CI	Upper CI
Intercept	-1.045	.057			-1.158	-.933
(100,200]	.438	.078	5.620	< .001	.285	.590
(200,300]	.805	.077	10.508	< .001	.654	.955
(300,400]	.871	.076	11.399	< .001	.722	1.021
(400,500]	.880	.076	11.510	< .001	.730	1.030
(500,600]	.903	.076	11.815	< .001	.753	1.052
(600,700]	.923	.076	12.084	< .001	.773	1.073
(700,800]	.892	.076	11.668	< .001	.742	1.041
(800,900]	.926	.076	12.127	< .001	.777	1.076
(900,1000]	.918	.076	12.015	< .001	.768	1.068
(1000,1100]	.912	.076	11.938	< .001	.762	1.062
(1100,1200]	.960	.076	12.572	< .001	.810	1.110
(1200,1300]	.952	.076	12.471	< .001	.803	1.102
(1300,1400]	.964	.076	12.629	< .001	.815	1.114
(1400,1500]	.918	.076	12.020	< .001	.769	1.068
(1500,1600]	.903	.076	11.813	< .001	.753	1.052
(1600,1700]	.966	.076	12.656	< .001	.817	1.116
(1700,1800]	1.008	.076	13.206	< .001	.858	1.158
(1800,1900]	1.069	.076	14.009	< .001	.920	1.219
(1900,2000]	1.049	.076	13.747	< .001	.900	1.199
(2000,2100]	1.066	.076	13.972	< .001	.917	1.216
(2100,2200]	1.110	.076	14.533	< .001	.960	1.259
(2200,2300]	1.158	.076	15.156	< .001	1.008	1.307
(2300,2400]	1.157	.076	15.145	< .001	1.007	1.306
(2400,2500]	1.137	.076	14.896	< .001	.988	1.287
(2500,2600]	1.158	.076	15.164	< .001	1.008	1.308
(2600,2700]	1.205	.076	15.763	< .001	1.055	1.355
(2700,2800]	1.258	.077	16.440	< .001	1.108	1.408
(2800,2900]	1.310	.077	17.097	< .001	1.160	1.460
(2900,3000]	1.365	.077	17.781	< .001	1.214	1.515
(3000,3100]	1.498	.077	19.402	< .001	1.346	1.649
(3100,3200]	1.574	.078	20.306	< .001	1.422	1.726
(3200,3300]	1.664	.078	21.345	< .001	1.511	1.817
(3300,3400]	1.678	.078	21.510	< .001	1.525	1.831
(3400,3500]	1.690	.078	21.638	< .001	1.537	1.843
(3500,3600]	1.744	.078	22.245	< .001	1.590	1.898
(3600,3700]	1.750	.078	22.307	< .001	1.596	1.904
(3700,3800]	1.666	.078	21.372	< .001	1.513	1.819
(3800,3900]	1.580	.078	20.379	< .001	1.428	1.732
(3900,4000]	1.478	.077	19.169	< .001	1.327	1.629
(4000,4100]	1.437	.077	18.668	< .001	1.286	1.588
(4100,4200]	1.355	.077	17.657	< .001	1.204	1.505
(4200,4300]	1.334	.077	17.404	< .001	1.184	1.485

(4300,4400]	1.256	.077	16.420	< .001	1.106	1.406
(4400,4500]	1.208	.076	15.804	< .001	1.058	1.358
(4500,4600]	1.074	.076	14.076	< .001	.925	1.224
(4600,4700]	.978	.076	12.805	< .001	.828	1.127
(4700,4800]	.857	.076	11.213	< .001	.708	1.007
(4800,4900]	.730	.077	9.515	< .001	.580	.881
(4900,5000]	.615	.077	7.970	< .001	.463	.766
(5000,5100]	.481	.078	6.197	< .001	.329	.634
(5100,5200]	.373	.078	4.773	< .001	.220	.527
(5200,5300]	.195	.079	2.454	.014	.039	.351
(5300,5400]	.117	.080	1.456	.145	-.040	.274
(5400,5500]	-.004	.081	-.052	.959	-.163	.155
(5500,5600]	-.134	.083	-1.621	.105	-.296	.028
(5600,5700]	-.247	.084	-2.946	.003	-.412	-.083
(5700,5800]	-.322	.085	-3.798	< .001	-.489	-.156
(5800,5900]	-.498	.088	-5.688	< .001	-.669	-.326
(5900,6000]	-.637	.090	-7.081	< .001	-.813	-.460
(6000,6100]	-.776	.093	-8.382	< .001	-.958	-.595
(6100,6200]	-.888	.095	-9.348	< .001	-1.075	-.702
(6200,6300]	-1.018	.098	-10.374	< .001	-1.210	-.825
(6300,6400]	-1.154	.102	-11.349	< .001	-1.354	-.955
(6400,6500]	-1.222	.104	-11.795	< .001	-1.426	-1.019
(6500,6600]	-1.272	.105	-12.100	< .001	-1.478	-1.066
(6600,6700]	-1.297	.106	-12.252	< .001	-1.505	-1.090
(6700,6800]	-1.404	.109	-12.843	< .001	-1.618	-1.190
(6800,6900]	-1.485	.112	-13.249	< .001	-1.705	-1.265
(6900,7000]	-1.593	.116	-13.728	< .001	-1.820	-1.365
(7000,7100]	-1.785	.124	-14.416	< .001	-2.028	-1.542
(7100,7200]	-1.858	.127	-14.625	< .001	-2.107	-1.609
(7200,7300]	-1.965	.132	-14.880	< .001	-2.224	-1.707
(7300,7400]	-2.123	.140	-15.152	< .001	-2.397	-1.848
(7400,7500]	-2.041	.136	-15.025	< .001	-2.307	-1.775
(7500,7600]	-2.091	.138	-15.106	< .001	-2.362	-1.819
(7600,7700]	-2.114	.140	-15.139	< .001	-2.387	-1.840
(7700,7800]	-2.148	.141	-15.185	< .001	-2.425	-1.871
(7800,7900]	-2.188	.144	-15.231	< .001	-2.469	-1.906
Web	.203	.086	2.372	.018	.035	.371
(100,200] * Web	-.296	.118	-2.508	.012	-.528	-.065
(200,300] * Web	-.681	.117	-5.801	< .001	-.911	-.451
(300,400] * Web	-.621	.117	-5.321	< .001	-.849	-.392
(400,500] * Web	-.625	.117	-5.361	< .001	-.854	-.397
(500,600] * Web	-.691	.117	-5.914	< .001	-.920	-.462
(600,700] * Web	-.830	.117	-7.066	.000	-1.060	-.600
(700,800] * Web	-.792	.117	-6.749	.000	-1.022	-.562
(800,900] * Web	-.774	.117	-6.608	< .001	-1.003	-.544
(900,1000] * Web	-.810	.117	-6.908	< .001	-1.040	-.581
(1000,1100]* Web	-.754	.117	-6.438	< .001	-.983	-.524
(1100,1200] * Web	-.801	.117	-6.848	< .001	-1.031	-.572
(1200,1300] * Web	-.726	.117	-6.224	< .001	-.955	-.497
(1300,1400] * Web	-.683	.116	-5.867	< .001	-.911	-.455
(1400,1500] * Web	-.719	.117	-6.154	< .001	-.948	-.490
(1500,1600] * Web	-.659	.117	-5.653	< .001	-.888	-.431
(1600,1700] * Web	-.685	.116	-5.885	< .001	-.913	-.457

(1700,1800] * Web	-.665	.116	-5.728	< .001	-.893	-.438
(1800,1900] * Web	-.805	.116	-6.912	< .001	-1.033	-.577
(1900,2000] * Web	-.720	.116	-6.192	< .001	-.947	-.492
(2000,2100] * Web	-.737	.116	-6.340	< .001	-.964	-.509
(2100,2200] * Web	-.764	.116	-6.575	< .001	-.991	-.536
(2200,2300] * Web	-.819	.116	-7.044	< .001	-1.046	-.591
(2300,2400] * Web	-.879	.116	-7.551	< .001	-1.108	-.651
(2400,2500] * Web	-.858	.116	-7.371	< .001	-1.086	-.630
(2500,2600] * Web	-.875	.116	-7.517	< .001	-1.103	-.647
(2600,2700] * Web	-.896	.116	-7.696	< .001	-1.124	-.667
(2700,2800] * Web	-.984	.117	-8.444	< .001	-1.213	-.756
(2800,2900] * Web	-1.024	.117	-8.787	< .001	-1.253	-.796
(2900,3000] * Web	-1.013	.116	-8.701	< .001	-1.241	-.785
(3000,3100] * Web	-1.071	.116	-9.194	< .001	-1.299	-.842
(3100,3200] * Web	-1.160	.117	-9.937	< .001	-1.388	-.931
(3200,3300] * Web	-1.239	.117	-10.591	< .001	-1.468	-1.009
(3300,3400] * Web	-1.301	.117	-11.107	< .001	-1.531	-1.072
(3400,3500] * Web	-1.266	.117	-10.819	< .001	-1.496	-1.037
(3500,3600] * Web	-1.274	.117	-10.876	< .001	-1.503	-1.044
(3600,3700] * Web	-1.217	.117	-10.401	< .001	-1.446	-.987
(3700,3800] * Web	-1.108	.117	-9.503	< .001	-1.337	-.880
(3800,3900] * Web	-1.077	.116	-9.251	< .001	-1.305	-.849
(3900,4000] * Web	-.968	.116	-8.331	< .001	-1.195	-.740
(4000,4100] * Web	-.881	.116	-7.594	< .001	-1.108	-.653
(4100,4200] * Web	-.886	.116	-7.639	< .001	-1.114	-.659
(4200,4300] * Web	-.866	.116	-7.471	< .001	-1.094	-.639
(4300,4400] * Web	-.743	.116	-6.423	< .001	-.970	-.517
(4400,4500] * Web	-.702	.116	-6.064	< .001	-.928	-.475
(4500,4600] * Web	-.608	.116	-5.254	< .001	-.835	-.381
(4600,4700] * Web	-.554	.116	-4.782	< .001	-.781	-.327
(4700,4800] * Web	-.520	.116	-4.475	< .001	-.748	-.292
(4800,4900] * Web	-.530	.117	-4.525	< .001	-.759	-.300
(4900,5000] * Web	-.476	.118	-4.043	< .001	-.706	-.245
(5000,5100] * Web	-.469	.119	-3.949	< .001	-.702	-.236
(5100,5200] * Web	-.518	.120	-4.309	< .001	-.754	-.283
(5200,5300] * Web	-.360	.121	-2.967	.003	-.597	-.122
(5300,5400] * Web	-.479	.124	-3.881	< .001	-.721	-.237
(5400,5500] * Web	-.452	.125	-3.606	< .001	-.697	-.206
(5500,5600] * Web	-.473	.128	-3.697	< .001	-.724	-.222
(5600,5700] * Web	-.416	.130	-3.211	.001	-.671	-.162
(5700,5800] * Web	-.369	.131	-2.821	.005	-.625	-.113
(5800,5900] * Web	-.308	.134	-2.295	.022	-.571	-.045
(5900,6000] * Web	-.225	.137	-1.645	.100	-.492	.043
(6000,6100] * Web	-.248	.141	-1.753	.080	-.525	.029
(6100,6200] * Web	-.269	.146	-1.852	.064	-.555	.016
(6200,6300] * Web	-.253	.150	-1.684	.092	-.547	.041
(6300,6400] * Web	-.162	.153	-1.056	.291	-.463	.139
(6400,6500] * Web	-.259	.159	-1.629	.103	-.571	.053
(6500,6600] * Web	-.352	.164	-2.144	.032	-.673	-.030
(6600,6700] * Web	-.314	.164	-1.914	.056	-.636	.008
(6700,6800] * Web	-.347	.171	-2.032	.042	-.681	-.012
(6800,6900] * Web	-.307	.174	-1.763	.078	-.647	.034
(6900,7000] * Web	-.234	.178	-1.318	.188	-.582	.114

(7000,7100] * Web	-.108	.185	-.585	.558	-.471	.254
(7100,7200] * Web	-.020	.187	-.106	.915	-.386	.346
(7200,7300] * Web	-.031	.194	-.161	.872	-.412	.350
(7300,7400] * Web	.134	.200	.673	.501	-.257	.526
(7400,7500] * Web	.010	.198	.052	.958	-.378	.399
(7500,7600] * Web	-.020	.203	-.099	.921	-.418	.378
(7600,7700] * Web	-.367	.221	-1.660	.097	-.800	.066
(7700,7800] * Web	-.399	.226	-1.769	.077	-.842	.043
(7800,7900] * Web	-.460	.233	-1.977	.048	-.917	-.004

Model B

Target Proportions ~ Bin Mode * Group* (quasiomial family). Model R^2 = .277. Reference level for Bin was (0,100], reference level for Mode was Lab.

	Est.	SE	*t*	*p*	Lower CI	Upper CI
Intercept	.298	.015			.268	.328
(100,200]	.073	.022	3.366	< .001	.030	.115
(200,300]	.170	.022	7.872	< .001	.127	.212
(300,400]	.175	.022	8.115	< .001	.133	.217
(400,500]	.175	.022	8.095	< .001	.132	.217
(500,600]	.197	.022	9.141	< .001	.155	.239
(600,700]	.205	.022	9.492	< .001	.162	.247
(700,800]	.188	.022	8.700	< .001	.145	.230
(800,900]	.218	.022	10.127	< .001	.176	.261
(900,1000]	.213	.022	9.888	< .001	.171	.255
(1000,1100]	.209	.022	9.711	< .001	.167	.252
(1100,1200]	.217	.022	10.075	< .001	.175	.260
(1200,1300]	.211	.022	9.798	< .001	.169	.254
(1300,1400]	.208	.022	9.663	< .001	.166	.251
(1400,1500]	.204	.022	9.446	< .001	.161	.246
(1500,1600]	.175	.022	8.130	< .001	.133	.218
(1600,1700]	.192	.022	8.911	< .001	.150	.234
(1700,1800]	.184	.022	8.541	< .001	.142	.226
(1800,1900]	.196	.022	9.085	< .001	.154	.238
(1900,2000]	.181	.022	8.391	< .001	.139	.223
(2000,2100]	.184	.022	8.514	< .001	.141	.226
(2100,2200]	.207	.022	9.577	< .001	.164	.249
(2200,2300]	.222	.022	10.283	< .001	.179	.264
(2300,2400]	.214	.022	9.929	< .001	.172	.256
(2400,2500]	.216	.022	10.023	< .001	.174	.258
(2500,2600]	.208	.022	9.667	< .001	.166	.251
(2600,2700]	.229	.022	10.636	< .001	.187	.272
(2700,2800]	.253	.022	11.713	< .001	.210	.295
(2800,2900]	.273	.022	12.643	< .001	.230	.315
(2900,3000]	.279	.022	12.959	< .001	.237	.322
(3000,3100]	.329	.022	15.243	< .001	.286	.371
(3100,3200]	.355	.022	16.480	< .001	.313	.398
(3200,3300]	.386	.022	17.922	< .001	.344	.429
(3300,3400]	.402	.022	18.647	< .001	.360	.444
(3400,3500]	.422	.022	19.588	< .001	.380	.465
(3500,3600]	.460	.022	21.341	< .001	.418	.502
(3600,3700]	.457	.022	21.190	< .001	.415	.499

(3700,3800]	.455	.022	21.080	< .001	.412	.497
(3800,3900]	.443	.022	20.520	< .001	.400	.485
(3900,4000]	.409	.022	18.965	< .001	.367	.451
(4000,4100]	.407	.022	18.883	< .001	.365	.449
(4100,4200]	.389	.022	18.040	< .001	.347	.431
(4200,4300]	.375	.022	17.370	< .001	.332	.417
(4300,4400]	.329	.022	15.248	< .001	.287	.371
(4400,4500]	.290	.022	13.432	< .001	.247	.332
(4500,4600]	.218	.022	10.125	< .001	.176	.261
(4600,4700]	.187	.022	8.662	< .001	.145	.229
(4700,4800]	.133	.022	6.146	< .001	.090	.175
(4800,4900]	.091	.022	4.228	< .001	.049	.133
(4900,5000]	.073	.022	3.380	.001	.031	.115
(5000,5100]	.047	.022	2.183	.029	.005	.089
(5100,5200]	.019	.022	.874	.382	-.023	.061
(5200,5300]	-.030	.022	-1.408	.159	-.073	.012
(5300,5400]	-.055	.022	-2.566	.010	-.098	-.013
(5400,5500]	-.096	.022	-4.443	< .001	-.138	-.054
(5500,5600]	-.137	.022	-6.346	< .001	-.179	-.095
(5600,5700]	-.170	.022	-7.884	< .001	-.212	-.128
(5700,5800]	-.189	.022	-8.757	< .001	-.231	-.147
(5800,5900]	-.224	.022	-10.404	< .001	-.267	-.182
(5900,6000]	-.237	.022	-11.002	< .001	-.280	-.195
(6000,6100]	-.250	.022	-11.610	< .001	-.293	-.208
(6100,6200]	-.261	.022	-12.081	< .001	-.303	-.218
(6200,6300]	-.279	.022	-12.941	< .001	-.321	-.237
(6300,6400]	-.286	.022	-13.283	< .001	-.329	-.244
(6400,6500]	-.288	.022	-13.371	< .001	-.331	-.246
(6500,6600]	-.288	.022	-13.371	< .001	-.331	-.246
(6600,6700]	-.292	.022	-13.543	< .001	-.334	-.250
(6700,6800]	-.294	.022	-13.629	< .001	-.336	-.252
(6800,6900]	-.296	.022	-13.715	< .001	-.338	-.253
(6900,7000]	-.296	.022	-13.715	< .001	-.338	-.253
(7000,7100]	-.296	.022	-13.715	< .001	-.338	-.253
(7100,7200]	-.296	.022	-13.715	< .001	-.338	-.253
(7200,7300]	-.296	.022	-13.715	< .001	-.338	-.253
(7300,7400]	-.298	.022	-13.801	< .001	-.340	-.255
(7400,7500]	-.298	.022	-13.801	< .001	-.340	-.255
(7500,7600]	-.298	.022	-13.801	< .001	-.340	-.255
(7600,7700]	-.298	.022	-13.801	< .001	-.340	-.255
(7700,7800]	-.298	.022	-13.801	< .001	-.340	-.255
(7800,7900]	-.298	.022	-13.801	< .001	-.340	-.255
(7900,8000]	-.298	.022	-13.801	< .001	-.340	-.255
(8000,8100]	-.298	.022	-13.801	< .001	-.340	-.255
(8100,8200]	-.298	.022	-13.801	< .001	-.340	-.255
(8200,8300]	-.298	.022	-13.801	< .001	-.340	-.255
(8300,8400]	-.298	.022	-13.801	< .001	-.340	-.255
(8400,8500]	-.298	.022	-13.801	< .001	-.340	-.255
(8500,8600]	-.298	.022	-13.801	< .001	-.340	-.255
(8600,8700]	-.298	.022	-13.801	< .001	-.340	-.255
(8700,8800]	-.298	.022	-13.801	< .001	-.340	-.255
(8800,8900]	-.298	.022	-13.801	< .001	-.340	-.255
(8900,9000]	-.298	.022	-13.801	< .001	-.340	-.255

(9000,9100]	-.298	.022	-13.801	< .001	-.340	-.255
(9100,9200]	-.298	.022	-13.801	< .001	-.340	-.255
(9200,9300]	-.298	.022	-13.801	< .001	-.340	-.255
(9300,9400]	-.298	.022	-13.801	< .001	-.340	-.255
(9400,9500]	-.298	.022	-13.801	< .001	-.340	-.255
(9500,9600]	-.298	.022	-13.801	< .001	-.340	-.255
(9600,9700]	-.298	.022	-13.801	< .001	-.340	-.255
(9700,9800]	-.298	.022	-13.801	< .001	-.340	-.255
(9800,9900]	-.298	.022	-13.801	< .001	-.340	-.255
(9900,10000]	-.298	.022	-13.801	< .001	-.340	-.255
(10000,10100]	-.298	.022	-13.801	< .001	-.340	-.255
(10100,10200]	-.298	.022	-13.801	< .001	-.340	-.255
(10200,10300]	-.298	.022	-13.801	< .001	-.340	-.255
(10300,10400]	-.298	.022	-13.801	< .001	-.340	-.255
(10400,10500]	-.298	.022	-13.801	< .001	-.340	-.255
(10500,10600]	-.298	.022	-13.801	< .001	-.340	-.255
(10600,10700]	-.298	.022	-13.801	< .001	-.340	-.255
(10700,10800]	-.298	.022	-13.801	< .001	-.340	-.255
(10800,10900]	-.298	.022	-13.801	< .001	-.340	-.255
(10900,11000]	-.298	.022	-13.801	< .001	-.340	-.255
(11100,11200]	-.298	.022	-13.801	< .001	-.340	-.255
(11200,11300]	-.298	.022	-13.801	< .001	-.340	-.255
(11300,11400]	-.298	.022	-13.801	< .001	-.340	-.255
(11400,11500]	-.298	.022	-13.801	< .001	-.340	-.255
(11500,11600]	-.298	.022	-13.801	< .001	-.340	-.255
(11600,11700]	-.298	.022	-13.801	< .001	-.340	-.255
(11700,11800]	-.298	.022	-13.801	< .001	-.340	-.255
(11800,11900]	-.298	.022	-13.801	< .001	-.340	-.255
(11900,12000]	-.298	.022	-13.801	< .001	-.340	-.255
(12000,30000]	-.298	.022	-13.801	< .001	-.340	-.255
Web	.015	.023	.655	.512	-.030	.060
PWA	-.092	.023	-4.043	< .001	-.136	-.047
(100,200] * Web	-.071	.032	-2.202	.028	-.135	-.008
(200,300] * Web	-.136	.032	-4.187	< .001	-.199	-.072
(300,400] * Web	-.114	.032	-3.518	< .001	-.178	-.051
(400,500] * Web	-.094	.032	-2.891	.004	-.157	-.030
(500,600] * Web	-.108	.032	-3.334	.001	-.172	-.045
(600,700] * Web	-.133	.032	-4.109	< .001	-.197	-.070
(700,800] * Web	-.109	.032	-3.365	.001	-.173	-.046
(800,900] * Web	-.145	.032	-4.459	< .001	-.208	-.081
(900,1000] * Web	-.132	.032	-4.083	.000	-.196	-.069
(1000,1100] * Web	-.139	.032	-4.290	< .001	-.203	-.076
(1100,1200] * Web	-.155	.032	-4.785	.000	-.219	-.092
(1200,1300] * Web	-.113	.032	-3.481	< .001	-.176	-.049
(1300,1400] * Web	-.122	.032	-3.753	< .001	-.185	-.058
(1400,1500] * Web	-.137	.032	-4.222	< .001	-.201	-.073
(1500,1600] * Web	-.099	.032	-3.058	.002	-.163	-.036
(1600,1700] * Web	-.122	.032	-3.758	< .001	-.185	-.058
(1700,1800] * Web	-.099	.032	-3.043	.002	-.162	-.035
(1800,1900] * Web	-.135	.032	-4.163	< .001	-.199	-.071
(1900,2000] * Web	-.110	.032	-3.377	.001	-.173	-.046
(2000,2100] * Web	-.085	.032	-2.628	.009	-.149	-.022
(2100,2200] * Web	-.093	.032	-2.865	.004	-.157	-.029

(2200,2300] * Web	-.117	.032	-3.612	< .001	-.181	-.054
(2300,2400] * Web	-.142	.032	-4.363	< .001	-.205	-.078
(2400,2500] * Web	-.155	.032	-4.787	< .001	-.219	-.092
(2500,2600] * Web	-.141	.032	-4.333	< .001	-.204	-.077
(2600,2700] * Web	-.145	.032	-4.472	< .001	-.209	-.081
(2700,2800] * Web	-.178	.032	-5.477	< .001	-.241	-.114
(2800,2900] * Web	-.195	.032	-6.011	< .001	-.259	-.131
(2900,3000] * Web	-.180	.032	-5.547	< .001	-.243	-.116
(3000,3100] * Web	-.205	.032	-6.307	< .001	-.268	-.141
(3100,3200] * Web	-.223	.032	-6.877	< .001	-.287	-.159
(3200,3300] * Web	-.252	.032	-7.764	< .001	-.315	-.188
(3300,3400] * Web	-.268	.032	-8.258	< .001	-.331	-.204
(3400,3500] * Web	-.299	.032	-9.232	< .001	-.363	-.236
(3500,3600] * Web	-.322	.032	-9.928	< .001	-.386	-.258
(3600,3700] * Web	-.279	.032	-8.601	< .001	-.343	-.215
(3700,3800] * Web	-.248	.032	-7.661	< .001	-.312	-.185
(3800,3900] * Web	-.261	.032	-8.047	< .001	-.325	-.197
(3900,4000] * Web	-.215	.032	-6.616	< .001	-.278	-.151
(4000,4100] * Web	-.202	.032	-6.237	< .001	-.266	-.139
(4100,4200] * Web	-.178	.032	-5.496	< .001	-.242	-.115
(4200,4300] * Web	-.150	.032	-4.617	< .001	-.213	-.086
(4300,4400] * Web	-.070	.032	-2.171	.030	-.134	-.007
(4400,4500] * Web	-.052	.032	-1.602	.109	-.116	.012
(4500,4600] * Web	.005	.032	.164	.870	-.058	.069
(4600,4700] * Web	.002	.032	.053	.958	-.062	.065
(4700,4800] * Web	.006	.032	.174	.862	-.058	.069
(4800,4900] * Web	-.008	.032	-.248	.804	-.072	.056
(4900,5000] * Web	-.010	.032	-.297	.766	-.073	.054
(5000,5100] * Web	-.022	.032	-.693	.488	-.086	.041
(5100,5200] * Web	-.026	.032	-.798	.425	-.089	.038
(5200,5300] * Web	.025	.032	.756	.450	-.039	.088
(5300,5400] * Web	.000	.032	.009	.993	-.063	.064
(5400,5500] * Web	-.007	.032	-.223	.824	-.071	.056
(5500,5600] * Web	.000	.032	-.005	.996	-.064	.063
(5600,5700] * Web	.020	.032	.621	.535	-.043	.084
(5700,5800] * Web	.019	.032	.587	.557	-.045	.083
(5800,5900] * Web	.043	.032	1.321	.186	-.021	.106
(5900,6000] * Web	.042	.032	1.286	.199	-.022	.105
(6000,6100] * Web	.020	.032	.607	.544	-.044	.083
(6100,6200] * Web	.020	.032	.631	.528	-.043	.084
(6200,6300] * Web	.034	.032	1.058	.290	-.029	.098
(6300,6400] * Web	.044	.032	1.358	.175	-.020	.108
(6400,6500] * Web	.037	.032	1.128	.259	-.027	.100
(6500,6600] * Web	.023	.032	.695	.487	-.041	.086
(6600,6700] * Web	.030	.032	.917	.359	-.034	.093
(6700,6800] * Web	.019	.032	.577	.564	-.045	.082
(6800,6900] * Web	.016	.032	.490	.624	-.048	.079
(6900,7000] * Web	.009	.032	.274	.784	-.055	.072
(7000,7100] * Web	.004	.032	.129	.897	-.059	.068
(7100,7200] * Web	.000	.032	-.015	.988	-.064	.063
(7200,7300] * Web	.002	.032	.057	.955	-.062	.065
(7300,7400] * Web	.001	.032	.042	.967	-.062	.065
(7400,7500] * Web	-.006	.032	-.175	.861	-.069	.058

(7500,7600] * Web	-.008	.032	-.247	.805	-.072	.056
(7600,7700] * Web	-.006	.032	-.175	.861	-.069	.058
(7700,7800] * Web	-.006	.032	-.175	.861	-.069	.058
(7800,7900] * Web	-.008	.032	-.247	.805	-.072	.056
(7900,8000] * Web	-.008	.032	-.247	.805	-.072	.056
(8000,8100] * Web	-.006	.032	-.175	.861	-.069	.058
(8100,8200] * Web	-.003	.032	-.102	.918	-.067	.060
(8200,8300] * Web	-.003	.032	-.102	.918	-.067	.060
(8300,8400] * Web	-.003	.032	-.102	.918	-.067	.060
(8400,8500] * Web	-.008	.032	-.247	.805	-.072	.056
(8500,8600] * Web	-.010	.032	-.319	.750	-.074	.053
(8600,8700] * Web	-.013	.032	-.391	.696	-.076	.051
(8700,8800] * Web	-.010	.032	-.319	.750	-.074	.053
(8800,8900] * Web	-.010	.032	-.319	.750	-.074	.053
(8900,9000] * Web	-.008	.032	-.247	.805	-.072	.056
(9000,9100] * Web	-.010	.032	-.319	.750	-.074	.053
(9100,9200] * Web	-.008	.032	-.247	.805	-.072	.056
(9200,9300] * Web	-.010	.032	-.319	.750	-.074	.053
(9300,9400] * Web	-.010	.032	-.319	.750	-.074	.053
(9400,9500] * Web	-.010	.032	-.319	.750	-.074	.053
(9500,9600] * Web	-.013	.032	-.391	.696	-.076	.051
(9600,9700] * Web	-.013	.032	-.391	.696	-.076	.051
(9700,9800] * Web	-.013	.032	-.391	.696	-.076	.051
(9800,9900] * Web	-.013	.032	-.391	.696	-.076	.051
(9900,10000] * Web	-.013	.032	-.391	.696	-.076	.051
(10000,10100] * Web	-.015	.032	-.463	.643	-.079	.049
(10100,10200] * Web	-.015	.032	-.463	.643	-.079	.049
(10200,10300] * Web	-.015	.032	-.463	.643	-.079	.049
(10300,10400] * Web	-.015	.032	-.463	.643	-.079	.049
(10400,10500] * Web	-.015	.032	-.463	.643	-.079	.049
(10500,10600] * Web	-.015	.032	-.463	.643	-.079	.049
(10600,10700] * Web	-.015	.032	-.463	.643	-.079	.049
(10700,10800] * Web	-.015	.032	-.463	.643	-.079	.049
(10800,10900] * Web	-.015	.032	-.463	.643	-.079	.049
(10900,11000] * Web	-.015	.032	-.463	.643	-.079	.049
(11000,11100] * Web	-.015	.032	-.463	.643	-.079	.049
(11100,11200] * Web	-.015	.032	-.463	.643	-.079	.049
(11200,11300] * Web	-.015	.032	-.463	.643	-.079	.049
(11300,11400] * Web	-.015	.032	-.463	.643	-.079	.049
(11400,11500] * Web	-.015	.032	-.463	.643	-.079	.049
(11500,11600] * Web	-.015	.032	-.463	.643	-.079	.049
(11600,11700] * Web	-.015	.032	-.463	.643	-.079	.049
(11700,11800] * Web	-.015	.032	-.463	.643	-.079	.049
(11800,11900] * Web	-.015	.032	-.463	.643	-.079	.049
(11900,12000] * Web	-.015	.032	-.463	.643	-.079	.049
(12000,30000] * Web	-.015	.032	-.463	.643	-.079	.049
(100,200] * PWA	.023	.032	.730	.465	-.040	.086
(200,300] * PWA	.038	.032	1.168	.243	-.025	.101
(300,400] * PWA	.050	.032	1.540	.124	-.013	.113
(400,500] * PWA	.044	.032	1.362	.173	-.019	.107
(500,600] * PWA	.016	.032	.499	.617	-.047	.079
(600,700] * PWA	-.003	.032	-.107	.915	-.066	.060
(700,800] * PWA	.030	.032	.937	.349	-.033	.093

(800,900] * PWA	-.001	.032	-.039	.969	-.064	.062
(900,1000] * PWA	.013	.032	.396	.692	-.050	.076
(1000,1100] * PWA	.023	.032	.714	.475	-.040	.086
(1100,1200] * PWA	.030	.032	.934	.350	-.033	.093
(1200,1300] * PWA	.048	.032	1.493	.135	-.015	.111
(1300,1400] * PWA	.069	.032	2.139	.032	.006	.132
(1400,1500] * PWA	.063	.032	1.953	.051	.000	.126
(1500,1600] * PWA	.102	.032	3.185	.001	.039	.165
(1600,1700] * PWA	.109	.032	3.391	.001	.046	.172
(1700,1800] * PWA	.129	.032	3.999	< .001	.066	.192
(1800,1900] * PWA	.114	.032	3.550	< .001	.051	.177
(1900,2000] * PWA	.125	.032	3.896	< .001	.062	.188
(2000,2100] * PWA	.099	.032	3.080	.002	.036	.162
(2100,2200] * PWA	.073	.032	2.275	.023	.010	.136
(2200,2300] * PWA	.068	.032	2.105	.035	.005	.131
(2300,2400] * PWA	.079	.032	2.447	.014	.016	.142
(2400,2500] * PWA	.070	.032	2.173	.030	.007	.133
(2500,2600] * PWA	.081	.032	2.515	.012	.018	.144
(2600,2700] * PWA	.056	.032	1.733	.083	-.007	.119
(2700,2800] * PWA	.030	.032	.948	.343	-.033	.093
(2800,2900] * PWA	.004	.032	.121	.903	-.059	.067
(2900,3000] * PWA	.004	.032	.116	.908	-.059	.067
(3000,3100] * PWA	-.018	.032	-.558	.577	-.081	.045
(3100,3200] * PWA	-.004	.032	-.130	.897	-.067	.059
(3200,3300] * PWA	-.021	.032	-.665	.506	-.084	.042
(3300,3400] * PWA	-.046	.032	-1.422	.155	-.109	.017
(3400,3500] * PWA	-.054	.032	-1.680	.093	-.117	.009
(3500,3600] * PWA	-.092	.032	-2.847	.004	-.155	-.029
(3600,3700] * PWA	-.067	.032	-2.086	.037	-.130	-.004
(3700,3800] * PWA	-.069	.032	-2.156	.031	-.132	-.006
(3800,3900] * PWA	-.068	.032	-2.121	.034	-.131	-.005
(3900,4000] * PWA	-.042	.032	-1.293	.196	-.105	.021
(4000,4100] * PWA	-.040	.032	-1.242	.214	-.103	.023
(4100,4200] * PWA	-.036	.032	-1.122	.262	-.099	.027
(4200,4300] * PWA	-.023	.032	-.711	.477	-.086	.040
(4300,4400] * PWA	.025	.032	.784	.433	-.038	.088
(4400,4500] * PWA	.048	.032	1.497	.135	-.015	.111
(4500,4600] * PWA	.109	.032	3.391	.001	.046	.172
(4600,4700] * PWA	.125	.032	3.883	< .001	.062	.188
(4700,4800] * PWA	.171	.032	5.305	< .001	.108	.234
(4800,4900] * PWA	.200	.032	6.208	< .001	.137	.263
(4900,5000] * PWA	.216	.032	6.713	< .001	.153	.279
(5000,5100] * PWA	.210	.032	6.543	< .001	.147	.273
(5100,5200] * PWA	.222	.032	6.904	< .001	.159	.285
(5200,5300] * PWA	.251	.032	7.815	< .001	.188	.314
(5300,5400] * PWA	.265	.032	8.231	< .001	.202	.328
(5400,5500] * PWA	.302	.032	9.410	< .001	.239	.365
(5500,5600] * PWA	.332	.032	10.323	< .001	.269	.395
(5600,5700] * PWA	.354	.032	11.024	< .001	.291	.417
(5700,5800] * PWA	.368	.032	11.460	< .001	.305	.431
(5800,5900] * PWA	.361	.032	11.217	< .001	.298	.424
(5900,6000] * PWA	.349	.032	10.845	< .001	.286	.412
(6000,6100] * PWA	.330	.032	10.274	< .001	.267	.393

(6100,6200] * PWA	.322	.032	10.006	< .001	.259	.385
(6200,6300] * PWA	.333	.032	10.365	< .001	.270	.396
(6300,6400] * PWA	.316	.032	9.841	< .001	.253	.379
(6400,6500] * PWA	.313	.032	9.741	< .001	.250	.376
(6500,6600] * PWA	.306	.032	9.517	< .001	.243	.369
(6600,6700] * PWA	.305	.032	9.500	< .001	.242	.368
(6700,6800] * PWA	.287	.032	8.943	< .001	.224	.350
(6800,6900] * PWA	.279	.032	8.669	< .001	.216	.342
(6900,7000] * PWA	.255	.032	7.917	< .001	.192	.318
(7000,7100] * PWA	.224	.032	6.955	< .001	.161	.287
(7100,7200] * PWA	.218	.032	6.767	< .001	.155	.281
(7200,7300] * PWA	.200	.032	6.222	< .001	.137	.263
(7300,7400] * PWA	.192	.032	5.988	< .001	.129	.255
(7400,7500] * PWA	.198	.032	6.161	< .001	.135	.261
(7500,7600] * PWA	.195	.032	6.056	< .001	.132	.258
(7600,7700] * PWA	.193	.032	6.012	< .001	.130	.256
(7700,7800] * PWA	.184	.032	5.733	< .001	.121	.247
(7800,7900] * PWA	.178	.032	5.538	< .001	.115	.241
(7900,8000] * PWA	.180	.032	5.609	< .001	.117	.243
(8000,8100] * PWA	.169	.032	5.269	< .001	.106	.232
(8100,8200] * PWA	.172	.032	5.350	< .001	.109	.235
(8200,8300] * PWA	.173	.032	5.387	< .001	.110	.236
(8300,8400] * PWA	.168	.032	5.239	< .001	.105	.231
(8400,8500] * PWA	.159	.032	4.955	< .001	.096	.222
(8500,8600] * PWA	.153	.032	4.745	< .001	.090	.216
(8600,8700] * PWA	.148	.032	4.615	< .001	.085	.211
(8700,8800] * PWA	.134	.032	4.163	< .001	.071	.197
(8800,8900] * PWA	.132	.032	4.097	< .001	.069	.195
(8900,9000] * PWA	.123	.032	3.812	< .001	.060	.186
(9000,9100] * PWA	.121	.032	3.776	< .001	.058	.184
(9100,9200] * PWA	.120	.032	3.740	< .001	.057	.183
(9200,9300] * PWA	.118	.032	3.663	< .001	.055	.181
(9300,9400] * PWA	.124	.032	3.853	< .001	.061	.187
(9400,9500] * PWA	.126	.032	3.917	< .001	.063	.189
(9500,9600] * PWA	.123	.032	3.815	< .001	.060	.186
(9600,9700] * PWA	.117	.032	3.642	< .001	.054	.180
(9700,9800] * PWA	.118	.032	3.670	< .001	.055	.181
(9800,9900] * PWA	.115	.032	3.564	< .001	.052	.178
(9900,10000] * PWA	.115	.032	3.564	< .001	.052	.178
(10000,10100] * PWA	.111	.032	3.439	.001	.048	.174
(10100,10200] * PWA	.111	.032	3.448	.001	.048	.174
(10200,10300] * PWA	.111	.032	3.467	.001	.048	.174
(10300,10400] * PWA	.108	.032	3.360	.001	.045	.171
(10400,10500] * PWA	.117	.032	3.635	< .001	.054	.180
(10500,10600] * PWA	.117	.032	3.635	< .001	.054	.180
(10600,10700] * PWA	.111	.032	3.447	.001	.048	.174
(10700,10800] * PWA	.115	.032	3.564	< .001	.052	.178
(10800,10900] * PWA	.117	.032	3.635	< .001	.054	.180
(10900,11000] * PWA	.117	.032	3.635	< .001	.054	.180
(11000,11100] * PWA	.121	.032	3.764	< .001	.058	.184
(11100,11200] * PWA	.115	.032	3.564	< .001	.052	.178
(11200,11300] * PWA	.111	.032	3.465	.001	.048	.174
(11300,11400] * PWA	.107	.032	3.328	.001	.044	.170

(11400,11500] * PWA	.106	.032	3.282	.001	.042	.169
(11500,11600] * PWA	.103	.032	3.192	.001	.040	.166
(11600,11700] * PWA	.096	.032	3.000	.003	.033	.159
(11700,11800] * PWA	.100	.032	3.118	.002	.037	.163
(11800,11900] * PWA	.099	.032	3.070	.002	.036	.162
(11900,12000] * PWA	.101	.032	3.141	.002	.038	.164
(12000,30000] * PWA	.108	.032	3.359	.001	.045	.171
Web * PWA	.062	.034	1.844	.065	-.004	.129
(100,200] * Web * PWA	.050	.048	1.044	.297	-.044	.144
(200,300] * Web * PWA	-.019	.048	-.389	.697	-.112	.075
(300,400] * Web * PWA	-.057	.048	-1.203	.229	-.151	.036
(400,500] * Web * PWA	-.080	.048	-1.666	.096	-.173	.014
(500,600] * Web * PWA	-.088	.048	-1.848	.065	-.182	.005
(600,700] * Web * PWA	-.076	.048	-1.589	.112	-.170	.018
(700,800] * Web * PWA	-.127	.048	-2.660	.008	-.221	-.033
(800,900] * Web * PWA	-.073	.048	-1.518	.129	-.166	.021
(900,1000] * Web * PWA	-.112	.048	-2.344	.019	-.206	-.018
(1000,1100] * Web * PWA	-.075	.048	-1.563	.118	-.168	.019
(1100,1200] * Web * PWA	-.071	.048	-1.484	.138	-.165	.023
(1200,1300] * Web * PWA	-.120	.048	-2.509	.012	-.214	-.026
(1300,1400] * Web * PWA	-.108	.048	-2.256	.024	-.201	-.014
(1400,1500] * Web * PWA	-.131	.048	-2.738	.006	-.225	-.037
(1500,1600] * Web * PWA	-.160	.048	-3.348	.001	-.254	-.066
(1600,1700] * Web * PWA	-.145	.048	-3.032	.002	-.239	-.051
(1700,1800] * Web * PWA	-.156	.048	-3.261	.001	-.249	-.062
(1800,1900] * Web * PWA	-.119	.048	-2.500	.012	-.213	-.026
(1900,2000] * Web * PWA	-.124	.048	-2.593	.010	-.218	-.030
(2000,2100] * Web * PWA	-.144	.048	-3.023	.002	-.238	-.051
(2100,2200] * Web * PWA	-.151	.048	-3.169	.002	-.245	-.058
(2200,2300] * Web * PWA	-.138	.048	-2.885	.004	-.232	-.044
(2300,2400] * Web * PWA	-.129	.048	-2.695	.007	-.222	-.035
(2400,2500] * Web * PWA	-.071	.048	-1.491	.136	-.165	.022
(2500,2600] * Web * PWA	-.091	.048	-1.895	.058	-.184	.003
(2600,2700] * Web * PWA	-.091	.048	-1.906	.057	-.185	.003
(2700,2800] * Web * PWA	-.071	.048	-1.486	.137	-.165	.023
(2800,2900] * Web * PWA	-.051	.048	-1.071	.284	-.145	.043
(2900,3000] * Web * PWA	-.079	.048	-1.662	.096	-.173	.014
(3000,3100] * Web * PWA	-.069	.048	-1.447	.148	-.163	.025
(3100,3200] * Web * PWA	-.110	.048	-2.294	.022	-.203	-.016
(3200,3300] * Web * PWA	-.059	.048	-1.236	.217	-.153	.035
(3300,3400] * Web * PWA	-.055	.048	-1.161	.246	-.149	.038
(3400,3500] * Web * PWA	-.016	.048	-.336	.737	-.110	.078
(3500,3600] * Web * PWA	.008	.048	.158	.874	-.086	.101
(3600,3700] * Web * PWA	-.037	.048	-.771	.440	-.131	.057
(3700,3800] * Web * PWA	-.076	.048	-1.589	.112	-.170	.018
(3800,3900] * Web * PWA	-.076	.048	-1.597	.110	-.170	.017
(3900,4000] * Web * PWA	-.113	.048	-2.367	.018	-.207	-.019
(4000,4100] * Web * PWA	-.090	.048	-1.875	.061	-.183	.004
(4100,4200] * Web * PWA	-.154	.048	-3.213	.001	-.247	-.060
(4200,4300] * Web * PWA	-.173	.048	-3.618	< .001	-.267	-.079
(4300,4400] * Web * PWA	-.262	.048	-5.492	< .001	-.356	-.169
(4400,4500] * Web * PWA	-.250	.048	-5.234	< .001	-.344	-.156
(4500,4600] * Web * PWA	-.313	.048	-6.547	< .001	-.406	-.219

(4600,4700] * Web * PWA	-.288	.048	-6.031	< .001	-.382	-.195
(4700,4800] * Web * PWA	-.292	.048	-6.100	< .001	-.385	-.198
(4800,4900] * Web * PWA	-.264	.048	-5.528	< .001	-.358	-.171
(4900,5000] * Web * PWA	-.255	.048	-5.341	< .001	-.349	-.162
(5000,5100] * Web * PWA	-.227	.048	-4.750	< .001	-.321	-.133
(5100,5200] * Web * PWA	-.228	.048	-4.774	< .001	-.322	-.134
(5200,5300] * Web * PWA	-.267	.048	-5.577	< .001	-.360	-.173
(5300,5400] * Web * PWA	-.260	.048	-5.437	< .001	-.353	-.166
(5400,5500] * Web * PWA	-.244	.048	-5.115	< .001	-.338	-.151
(5500,5600] * Web * PWA	-.249	.048	-5.212	< .001	-.343	-.155
(5600,5700] * Web * PWA	-.262	.048	-5.488	< .001	-.356	-.169
(5700,5800] * Web * PWA	-.230	.048	-4.821	< .001	-.324	-.137
(5800,5900] * Web * PWA	-.245	.048	-5.132	< .001	-.339	-.152
(5900,6000] * Web * PWA	-.222	.048	-4.643	< .001	-.316	-.128
(6000,6100] * Web * PWA	-.166	.048	-3.468	.001	-.259	-.072
(6100,6200] * Web * PWA	-.162	.048	-3.396	.001	-.256	-.069
(6200,6300] * Web * PWA	-.174	.048	-3.650	< .001	-.268	-.081
(6300,6400] * Web * PWA	-.172	.048	-3.596	< .001	-.266	-.078
(6400,6500] * Web * PWA	-.176	.048	-3.693	< .001	-.270	-.083
(6500,6600] * Web * PWA	-.167	.048	-3.497	< .001	-.261	-.073
(6600,6700] * Web * PWA	-.171	.048	-3.587	< .001	-.265	-.078
(6700,6800] * Web * PWA	-.150	.048	-3.136	.002	-.244	-.056
(6800,6900] * Web * PWA	-.147	.048	-3.076	.002	-.241	-.053
(6900,7000] * Web * PWA	-.114	.048	-2.395	.017	-.208	-.021
(7000,7100] * Web * PWA	-.080	.048	-1.677	.093	-.174	.014
(7100,7200] * Web * PWA	-.064	.048	-1.342	.180	-.158	.030
(7200,7300] * Web * PWA	-.071	.048	-1.495	.135	-.165	.022
(7300,7400] * Web * PWA	-.055	.048	-1.150	.250	-.149	.039
(7400,7500] * Web * PWA	-.056	.048	-1.175	.240	-.150	.038
(7500,7600] * Web * PWA	-.069	.048	-1.443	.149	-.163	.025
(7600,7700] * Web * PWA	-.102	.048	-2.127	.033	-.195	-.008
(7700,7800] * Web * PWA	-.094	.048	-1.967	.049	-.188	.000
(7800,7900] * Web * PWA	-.087	.048	-1.814	.070	-.180	.007
(7900,8000] * Web * PWA	-.092	.048	-1.917	.055	-.185	.002
(8000,8100] * Web * PWA	-.099	.048	-2.070	.038	-.193	-.005
(8100,8200] * Web * PWA	-.099	.048	-2.063	.039	-.192	-.005
(8200,8300] * Web * PWA	-.105	.048	-2.198	.028	-.199	-.011
(8300,8400] * Web * PWA	-.098	.048	-2.043	.041	-.191	-.004
(8400,8500] * Web * PWA	-.089	.048	-1.865	.062	-.183	.005
(8500,8600] * Web * PWA	-.083	.048	-1.730	.084	-.176	.011
(8600,8700] * Web * PWA	-.084	.048	-1.759	.079	-.178	.010
(8700,8800] * Web * PWA	-.068	.048	-1.421	.155	-.162	.026
(8800,8900] * Web * PWA	-.060	.048	-1.266	.205	-.154	.033
(8900,9000] * Web * PWA	-.060	.048	-1.262	.207	-.154	.033
(9000,9100] * Web * PWA	-.065	.048	-1.355	.176	-.158	.029
(9100,9200] * Web * PWA	-.071	.048	-1.491	.136	-.165	.022
(9200,9300] * Web * PWA	-.066	.048	-1.389	.165	-.160	.027
(9300,9400] * Web * PWA	-.078	.048	-1.628	.104	-.171	.016
(9400,9500] * Web * PWA	-.085	.048	-1.782	.075	-.179	.009
(9500,9600] * Web * PWA	-.066	.048	-1.388	.165	-.160	.027
(9600,9700] * Web * PWA	-.061	.048	-1.271	.204	-.154	.033
(9700,9800] * Web * PWA	-.062	.048	-1.290	.197	-.155	.032
(9800,9900] * Web * PWA	-.053	.048	-1.108	.268	-.147	.041

(9900,10000] * Web * PWA	-.062	.048	-1.302	.193	-.156	.031
(10000,10100] * Web * PWA	-.070	.048	-1.473	.141	-.164	.023
(10100,10200] * Web * PWA	-.073	.048	-1.535	.125	-.167	.020
(10200,10300] * Web * PWA	-.062	.048	-1.298	.194	-.156	.032
(10300,10400] * Web * PWA	-.049	.048	-1.032	.302	-.143	.044
(10400,10500] * Web * PWA	-.061	.048	-1.273	.203	-.154	.033
(10500,10600] * Web * PWA	-.061	.048	-1.273	.203	-.154	.033
(10600,10700] * Web * PWA	-.052	.048	-1.091	.275	-.146	.042
(10700,10800] * Web * PWA	-.051	.048	-1.059	.290	-.144	.043
(10800,10900] * Web * PWA	-.048	.048	-.996	.319	-.141	.046
(10900,11000] * Web * PWA	-.048	.048	-.996	.319	-.141	.046
(11000,11100] * Web * PWA	-.049	.048	-1.027	.304	-.143	.045
(11100,11200] * Web * PWA	-.048	.048	-1.004	.316	-.142	.046
(11200,11300] * Web * PWA	-.045	.048	-.937	.349	-.138	.049
(11300,11400] * Web * PWA	-.038	.048	-.789	.430	-.131	.056
(11400,11500] * Web * PWA	-.042	.048	-.869	.385	-.135	.052
(11500,11600] * Web * PWA	-.041	.048	-.864	.388	-.135	.052
(11600,11700] * Web * PWA	-.038	.048	-.790	.429	-.131	.056
(11700,11800] * Web * PWA	-.046	.048	-.953	.341	-.139	.048
(11800,11900] * Web * PWA	-.040	.048	-.838	.402	-.134	.054
(11900,12000] * Web * PWA	-.040	.048	-.830	.407	-.133	.054
(12000,30000] * Web * PWA	-.041	.048	-.860	.390	-.135	.053

Model C

*Target Proportions ~ Bin * Type * Mode* (quasiomial family). Model $R^2 = .215$. Reference level for Bin was (0,100], reference level for Mode was Lab, reference level for Type was Active.

	Est	SE	t	p	LowerCI	UpperCI
Intercept	-1.033	.124			-1.276	-.791
(100,200]	.471	.168	2.813	.005	.143	.800
(200,300]	.878	.165	5.327	< .001	.555	1.201
(300,400]	.980	.165	5.952	< .001	.657	1.303
(400,500]	.905	.165	5.488	< .001	.582	1.228
(500,600]	.921	.165	5.592	< .001	.598	1.244
(600,700]	.926	.165	5.620	< .001	.603	1.249
(700,800]	.879	.165	5.333	< .001	.556	1.202
(800,900]	1.026	.165	6.233	< .001	.704	1.349
(900,1000]	.985	.165	5.982	< .001	.662	1.308
(1000,1100]	.897	.165	5.444	< .001	.574	1.220
(1100,1200]	.949	.165	5.759	< .001	.626	1.271
(1200,1300]	.944	.165	5.731	< .001	.621	1.267
(1300,1400]	1.047	.165	6.356	< .001	.724	1.369
(1400,1500]	1.043	.165	6.334	< .001	.720	1.366
(1500,1600]	.934	.165	5.668	< .001	.611	1.257
(1600,1700]	.971	.165	5.899	< .001	.649	1.294
(1700,1800]	.930	.165	5.646	< .001	.607	1.253
(1800,1900]	.952	.165	5.781	< .001	.629	1.275
(1900,2000]	.903	.165	5.479	< .001	.580	1.226
(2000,2100]	.901	.165	5.469	< .001	.578	1.224
(2100,2200]	.843	.165	5.110	< .001	.520	1.166
(2200,2300]	.860	.165	5.212	< .001	.536	1.183
(2300,2400]	.893	.165	5.419	< .001	.570	1.216
(2400,2500]	.932	.165	5.659	< .001	.609	1.255

(2500,2600]	.922	.165	5.597	< .001	.599	1.245
(2600,2700]	1.068	.165	6.484	< .001	.745	1.390
(2700,2800]	1.198	.165	7.267	< .001	.875	1.522
(2800,2900]	1.163	.165	7.056	< .001	.840	1.486
(2900,3000]	1.238	.165	7.499	< .001	.914	1.561
(3000,3100]	1.418	.166	8.540	< .001	1.092	1.743
(3100,3200]	1.514	.167	9.082	< .001	1.188	1.841
(3200,3300]	1.571	.167	9.391	< .001	1.243	1.899
(3300,3400]	1.510	.167	9.059	< .001	1.184	1.837
(3400,3500]	1.557	.167	9.317	< .001	1.230	1.885
(3500,3600]	1.677	.168	9.954	< .001	1.347	2.007
(3600,3700]	1.701	.169	10.079	< .001	1.370	2.032
(3700,3800]	1.780	.170	10.484	< .001	1.448	2.113
(3800,3900]	1.752	.169	10.341	< .001	1.420	2.084
(3900,4000]	1.553	.167	9.292	< .001	1.225	1.880
(4000,4100]	1.560	.167	9.330	< .001	1.232	1.887
(4100,4200]	1.527	.167	9.149	< .001	1.200	1.854
(4200,4300]	1.498	.167	8.990	< .001	1.171	1.824
(4300,4400]	1.392	.166	8.393	< .001	1.067	1.717
(4400,4500]	1.263	.165	7.650	< .001	.940	1.587
(4500,4600]	.921	.165	5.590	< .001	.598	1.244
(4600,4700]	.783	.165	4.737	< .001	.459	1.106
(4700,4800]	.639	.166	3.847	< .001	.313	.964
(4800,4900]	.481	.167	2.872	.004	.153	.809
(4900,5000]	.411	.168	2.442	.015	.081	.741
(5000,5100]	.241	.170	1.411	.158	-.094	.575
(5100,5200]	.177	.172	1.034	.301	-.159	.514
(5200,5300]	.012	.175	.071	.943	-.330	.355
(5300,5400]	-.128	.178	-.718	.473	-.476	.221
(5400,5500]	-.216	.180	-1.203	.229	-.569	.136
(5500,5600]	-.413	.186	-2.224	.026	-.777	-.049
(5600,5700]	-.511	.189	-2.705	.007	-.881	-.141
(5700,5800]	-.632	.193	-3.266	.001	-1.011	-.253
(5800,5900]	-.816	.201	-4.056	< .001	-1.210	-.422
(5900,6000]	-.849	.203	-4.189	< .001	-1.246	-.452
(6000,6100]	-1.118	.217	-5.159	< .001	-1.543	-.693
(6100,6200]	-1.070	.214	-5.001	< .001	-1.490	-.651
(6200,6300]	-1.145	.218	-5.245	< .001	-1.573	-.717
(6300,6400]	-1.237	.224	-5.525	< .001	-1.676	-.798
(6400,6500]	-1.139	.218	-5.225	< .001	-1.566	-.712
(6500,6600]	-1.171	.220	-5.325	< .001	-1.601	-.740
(6600,6700]	-1.200	.222	-5.416	< .001	-1.635	-.766
(6700,6800]	-1.362	.232	-5.865	< .001	-1.817	-.907
(6800,6900]	-1.391	.234	-5.938	< .001	-1.850	-.932
(6900,7000]	-1.510	.243	-6.213	< .001	-1.986	-1.033
(7000,7100]	-1.667	.256	-6.517	< .001	-2.168	-1.165
(7100,7200]	-1.497	.242	-6.185	< .001	-1.971	-1.023
(7200,7300]	-1.548	.246	-6.293	< .001	-2.030	-1.066
(7300,7400]	-1.606	.251	-6.408	< .001	-2.097	-1.115
(7400,7500]	-1.612	.251	-6.419	< .001	-2.104	-1.120
(7500,7600]	-1.681	.257	-6.541	< .001	-2.184	-1.177
(7600,7700]	-1.832	.271	-6.767	< .001	-2.362	-1.301
(7700,7800]	-1.954	.283	-6.909	< .001	-2.508	-1.400

(7800,7900]	-2.040	.292	-6.988	< .001	-2.613	-1.468
Locative	-.185	.180	-1.028	.304	-.539	.168
Passive	.079	.174	.455	.649	-.262	.421
Web	.241	.170	1.416	.157	-.093	.576
(100,200] * Locative	-.098	.244	-.401	.689	-.577	.381
(200,300] * Locative	-.066	.239	-.278	.781	-.534	.402
(300,400] * Locative	-.132	.238	-.555	.579	-.600	.335
(400,500] * Locative	.005	.238	.023	.982	-.462	.473
(500,600] * Locative	.099	.238	.417	.677	-.367	.565
(600,700] * Locative	-.028	.238	-.116	.908	-.495	.439
(700,800] * Locative	.076	.238	.320	.749	-.391	.543
(800,900] * Locative	-.009	.238	-.037	.971	-.475	.457
(900,1000] * Locative	.190	.238	.800	.423	-.276	.656
(1000,1100] * Locative	.320	.238	1.346	.178	-.146	.786
(1100,1200] * Locative	.306	.238	1.287	.198	-.160	.771
(1200,1300] * Locative	.290	.238	1.220	.223	-.176	.756
(1300,1400] * Locative	.157	.238	.660	.509	-.309	.622
(1400,1500] * Locative	.132	.238	.557	.578	-.333	.598
(1500,1600] * Locative	.175	.238	.736	.462	-.291	.641
(1600,1700] * Locative	.253	.238	1.065	.287	-.213	.719
(1700,1800] * Locative	.281	.238	1.182	.237	-.185	.747
(1800,1900] * Locative	.261	.238	1.100	.271	-.204	.727
(1900,2000] * Locative	.297	.238	1.251	.211	-.168	.763
(2000,2100] * Locative	.215	.238	.905	.365	-.251	.681
(2100,2200] * Locative	.455	.238	1.915	.056	-.011	.922
(2200,2300] * Locative	.495	.238	2.083	.037	.029	.962
(2300,2400] * Locative	.398	.238	1.673	.094	-.068	.864
(2400,2500] * Locative	.300	.238	1.262	.207	-.166	.766
(2500,2600] * Locative	.337	.238	1.420	.156	-.128	.803
(2600,2700] * Locative	.212	.238	.894	.372	-.253	.678
(2700,2800] * Locative	.016	.238	.069	.945	-.450	.482
(2800,2900] * Locative	.158	.238	.665	.506	-.308	.624
(2900,3000] * Locative	.067	.238	.280	.779	-.400	.533
(3000,3100] * Locative	.048	.239	.201	.841	-.420	.516
(3100,3200] * Locative	.165	.240	.688	.491	-.306	.637
(3200,3300] * Locative	.339	.243	1.396	.163	-.137	.814
(3300,3400] * Locative	.432	.242	1.782	.075	-.043	.907
(3400,3500] * Locative	.433	.243	1.779	.075	-.044	.910
(3500,3600] * Locative	.378	.245	1.543	.123	-.102	.858
(3600,3700] * Locative	.303	.244	1.241	.215	-.176	.783
(3700,3800] * Locative	.150	.244	.614	.539	-.329	.629
(3800,3900] * Locative	.203	.244	.829	.407	-.276	.682
(3900,4000] * Locative	.373	.243	1.540	.124	-.102	.849
(4000,4100] * Locative	.416	.243	1.711	.087	-.060	.892
(4100,4200] * Locative	.334	.242	1.382	.167	-.140	.808
(4200,4300] * Locative	.432	.242	1.782	.075	-.043	.907
(4300,4400] * Locative	.595	.242	2.456	.014	.120	1.070
(4400,4500] * Locative	.718	.242	2.972	.003	.245	1.192
(4500,4600] * Locative	1.125	.242	4.643	< .001	.650	1.599
(4600,4700] * Locative	1.254	.242	5.174	< .001	.779	1.730
(4700,4800] * Locative	1.269	.242	5.253	< .001	.796	1.743
(4800,4900] * Locative	1.394	.242	5.752	< .001	.919	1.869
(4900,5000] * Locative	1.499	.243	6.164	< .001	1.022	1.976

(5000,5100] * Locative	1.618	.244	6.623	< .001	1.139	2.097
(5100,5200] * Locative	1.539	.244	6.311	< .001	1.061	2.018
(5200,5300] * Locative	1.464	.245	5.977	< .001	.984	1.944
(5300,5400] * Locative	1.447	.247	5.861	< .001	.963	1.931
(5400,5500] * Locative	1.303	.248	5.245	< .001	.816	1.791
(5500,5600] * Locative	1.346	.253	5.317	< .001	.850	1.842
(5600,5700] * Locative	1.290	.256	5.036	< .001	.788	1.792
(5700,5800] * Locative	1.312	.260	5.046	< .001	.803	1.822
(5800,5900] * Locative	1.157	.269	4.304	< .001	.630	1.684
(5900,6000] * Locative	1.020	.272	3.749	< .001	.487	1.554
(6000,6100] * Locative	1.160	.285	4.077	< .001	.603	1.718
(6100,6200] * Locative	.910	.286	3.180	.001	.349	1.471
(6200,6300] * Locative	.829	.293	2.833	.005	.255	1.403
(6300,6400] * Locative	.757	.301	2.517	.012	.168	1.347
(6400,6500] * Locative	.574	.299	1.921	.055	-.012	1.160
(6500,6600] * Locative	.612	.300	2.041	.041	.024	1.200
(6600,6700] * Locative	.528	.305	1.731	.083	-.070	1.125
(6700,6800] * Locative	.613	.315	1.945	.052	-.005	1.230
(6800,6900] * Locative	.659	.316	2.085	.037	.040	1.278
(6900,7000] * Locative	.585	.329	1.776	.076	-.061	1.231
(7000,7100] * Locative	.342	.357	.957	.338	-.358	1.042
(7100,7200] * Locative	-.020	.359	-.055	.956	-.723	.683
(7200,7300] * Locative	-.249	.382	-.653	.514	-.997	.499
(7300,7400] * Locative	-.495	.412	-1.201	.230	-1.303	.313
(7400,7500] * Locative	-.480	.412	-1.166	.244	-1.287	.327
(7500,7600] * Locative	-.604	.436	-1.387	.165	-1.458	.250
(7600,7700] * Locative	-.198	.418	-.474	.636	-1.017	.621
(7700,7800] * Locative	-.089	.427	-.209	.835	-.926	.748
(7800,7900] * Locative	-.048	.437	-.111	.912	-.906	.809
(100,200] * Passive	-.118	.238	-.497	.619	-.584	.348
(200,300] * Passive	-.055	.233	-.236	.814	-.512	.402
(300,400] * Passive	-.167	.233	-.717	.473	-.624	.290
(400,500] * Passive	-.111	.233	-.476	.634	-.569	.346
(500,600] * Passive	-.132	.233	-.565	.572	-.589	.326
(600,700] * Passive	-.035	.233	-.151	.880	-.492	.422
(700,800] * Passive	-.021	.233	-.090	.929	-.478	.436
(800,900] * Passive	-.178	.233	-.764	.445	-.635	.279
(900,1000] * Passive	-.239	.233	-1.023	.306	-.696	.219
(1000,1100] * Passive	-.093	.233	-.398	.690	-.550	.364
(1100,1200] * Passive	-.100	.233	-.429	.668	-.557	.357
(1200,1300] * Passive	-.045	.233	-.193	.847	-.502	.412
(1300,1400] * Passive	-.144	.233	-.618	.537	-.601	.313
(1400,1500] * Passive	-.197	.233	-.847	.397	-.654	.260
(1500,1600] * Passive	-.038	.233	-.163	.871	-.495	.419
(1600,1700] * Passive	.010	.233	.042	.967	-.447	.467
(1700,1800] * Passive	.117	.233	.501	.617	-.340	.574
(1800,1900] * Passive	.133	.233	.570	.569	-.324	.590
(1900,2000] * Passive	.125	.233	.538	.591	-.332	.583
(2000,2100] * Passive	.102	.233	.435	.663	-.356	.559
(2100,2200] * Passive	.174	.233	.746	.456	-.283	.631
(2200,2300] * Passive	.238	.233	1.022	.307	-.219	.696
(2300,2400] * Passive	.203	.233	.868	.385	-.255	.660
(2400,2500] * Passive	.158	.233	.679	.497	-.299	.616

(2500,2600] * Passive	.118	.233	.508	.612	-.339	.576
(2600,2700] * Passive	-.081	.233	-.346	.730	-.537	.376
(2700,2800] * Passive	-.136	.233	-.582	.560	-.593	.321
(2800,2900] * Passive	-.072	.233	-.309	.757	-.529	.385
(2900,3000] * Passive	-.125	.234	-.533	.594	-.582	.333
(3000,3100] * Passive	-.166	.235	-.707	.480	-.626	.294
(3100,3200] * Passive	-.164	.236	-.697	.486	-.626	.298
(3200,3300] * Passive	-.203	.236	-.861	.389	-.666	.259
(3300,3400] * Passive	-.052	.236	-.218	.827	-.515	.411
(3400,3500] * Passive	.024	.237	.100	.921	-.442	.489
(3500,3600] * Passive	.000	.239	-.001	.999	-.469	.469
(3600,3700] * Passive	.110	.241	.455	.649	-.363	.582
(3700,3800] * Passive	-.028	.241	-.116	.908	-.500	.445
(3800,3900] * Passive	-.153	.239	-.640	.522	-.622	.316
(3900,4000] * Passive	-.010	.237	-.040	.968	-.474	.455
(4000,4100] * Passive	-.084	.237	-.354	.724	-.548	.380
(4100,4200] * Passive	-.120	.236	-.509	.611	-.583	.342
(4200,4300] * Passive	-.233	.235	-.989	.322	-.693	.228
(4300,4400] * Passive	-.366	.234	-1.566	.117	-.825	.092
(4400,4500] * Passive	-.454	.234	-1.945	.052	-.912	.004
(4500,4600] * Passive	-.353	.234	-1.507	.132	-.812	.106
(4600,4700] * Passive	-.370	.235	-1.574	.116	-.832	.091
(4700,4800] * Passive	-.394	.237	-1.661	.097	-.860	.071
(4800,4900] * Passive	-.423	.241	-1.758	.079	-.894	.049
(4900,5000] * Passive	-.476	.243	-1.959	.050	-.951	.000
(5000,5100] * Passive	-.496	.248	-2.002	.045	-.981	-.010
(5100,5200] * Passive	-.568	.251	-2.264	.024	-1.060	-.076
(5200,5300] * Passive	-.510	.256	-1.996	.046	-1.011	-.009
(5300,5400] * Passive	-.315	.256	-1.227	.220	-.817	.188
(5400,5500] * Passive	-.205	.257	-.797	.425	-.710	.299
(5500,5600] * Passive	-.092	.263	-.349	.727	-.608	.424
(5600,5700] * Passive	-.112	.269	-.417	.677	-.638	.414
(5700,5800] * Passive	.026	.271	.097	.923	-.506	.558
(5800,5900] * Passive	.069	.281	.245	.806	-.481	.619
(5900,6000] * Passive	-.038	.286	-.134	.894	-.599	.522
(6000,6100] * Passive	.126	.300	.420	.675	-.462	.713
(6100,6200] * Passive	-.043	.302	-.142	.887	-.635	.549
(6200,6300] * Passive	-.065	.309	-.211	.833	-.671	.541
(6300,6400] * Passive	-.136	.320	-.426	.670	-.765	.492
(6400,6500] * Passive	-.343	.322	-1.065	.287	-.973	.288
(6500,6600] * Passive	-.419	.329	-1.273	.203	-1.064	.226
(6600,6700] * Passive	-.327	.327	-1.001	.317	-.968	.313
(6700,6800] * Passive	-.283	.341	-.832	.406	-.951	.384
(6800,6900] * Passive	-.558	.363	-1.539	.124	-1.268	.153
(6900,7000] * Passive	-.538	.376	-1.432	.152	-1.275	.198
(7000,7100] * Passive	-.378	.384	-.985	.325	-1.131	.375
(7100,7200] * Passive	-.724	.390	-1.855	.064	-1.489	.041
(7200,7300] * Passive	-.846	.410	-2.066	.039	-1.649	-.043
(7300,7400] * Passive	-.794	.413	-1.922	.055	-1.604	.016
(7400,7500] * Passive	-.576	.393	-1.466	.143	-1.347	.194
(7500,7600] * Passive	-.375	.386	-.972	.331	-1.131	.381
(7600,7700] * Passive	-.261	.398	-.657	.511	-1.041	.519
(7700,7800] * Passive	-.278	.418	-.666	.506	-1.097	.541

(7800,7900] * Passive	-.274	.431	-.636	.525	-1.120	.571
(100,200] * Web	-.328	.234	-1.397	.162	-.787	.132
(200,300] * Web	-.629	.232	-2.716	.007	-1.083	-.175
(300,400] * Web	-.738	.232	-3.185	.001	-1.192	-.284
(400,500] * Web	-.591	.231	-2.558	.011	-1.045	-.138
(500,600] * Web	-.553	.231	-2.396	.017	-1.006	-.101
(600,700] * Web	-.664	.232	-2.868	.004	-1.118	-.210
(700,800] * Web	-.572	.231	-2.475	.013	-1.026	-.119
(800,900] * Web	-.752	.231	-3.248	.001	-1.205	-.298
(900,1000] * Web	-.743	.232	-3.207	.001	-1.197	-.289
(1000,1100] * Web	-.714	.232	-3.074	.002	-1.169	-.259
(1100,1200] * Web	-.648	.231	-2.803	.005	-1.102	-.195
(1200,1300] * Web	-.618	.231	-2.675	.007	-1.071	-.165
(1300,1400] * Web	-.639	.231	-2.770	.006	-1.091	-.187
(1400,1500] * Web	-.794	.232	-3.429	.001	-1.248	-.340
(1500,1600] * Web	-.633	.231	-2.739	.006	-1.087	-.180
(1600,1700] * Web	-.795	.232	-3.423	.001	-1.250	-.340
(1700,1800] * Web	-.636	.231	-2.750	.006	-1.090	-.183
(1800,1900] * Web	-.665	.231	-2.873	.004	-1.118	-.211
(1900,2000] * Web	-.533	.231	-2.307	.021	-.985	-.080
(2000,2100] * Web	-.419	.230	-1.819	.069	-.871	.032
(2100,2200] * Web	-.492	.231	-2.128	.033	-.945	-.039
(2200,2300] * Web	-.553	.231	-2.390	.017	-1.006	-.099
(2300,2400] * Web	-.567	.231	-2.454	.014	-1.020	-.114
(2400,2500] * Web	-.626	.231	-2.706	.007	-1.079	-.172
(2500,2600] * Web	-.603	.231	-2.607	.009	-1.056	-.150
(2600,2700] * Web	-.748	.231	-3.237	.001	-1.201	-.295
(2700,2800] * Web	-1.028	.232	-4.425	< .001	-1.484	-.573
(2800,2900] * Web	-.960	.232	-4.136	< .001	-1.414	-.505
(2900,3000] * Web	-.995	.232	-4.292	< .001	-1.450	-.541
(3000,3100] * Web	-1.111	.232	-4.786	< .001	-1.566	-.656
(3100,3200] * Web	-1.240	.233	-5.324	< .001	-1.696	-.783
(3200,3300] * Web	-1.233	.233	-5.293	< .001	-1.689	-.776
(3300,3400] * Web	-1.105	.232	-4.760	< .001	-1.559	-.650
(3400,3500] * Web	-1.100	.232	-4.738	< .001	-1.555	-.645
(3500,3600] * Web	-1.250	.233	-5.361	< .001	-1.708	-.793
(3600,3700] * Web	-1.108	.233	-4.760	< .001	-1.565	-.652
(3700,3800] * Web	-1.145	.234	-4.905	< .001	-1.603	-.688
(3800,3900] * Web	-1.251	.234	-5.356	< .001	-1.709	-.793
(3900,4000] * Web	-1.040	.232	-4.484	< .001	-1.494	-.585
(4000,4100] * Web	-1.084	.232	-4.669	< .001	-1.539	-.629
(4100,4200] * Web	-.953	.232	-4.114	< .001	-1.406	-.499
(4200,4300] * Web	-.985	.232	-4.253	< .001	-1.439	-.531
(4300,4400] * Web	-.905	.231	-3.918	< .001	-1.358	-.452
(4400,4500] * Web	-.732	.230	-3.176	.001	-1.183	-.280
(4500,4600] * Web	-.519	.231	-2.252	.024	-.972	-.067
(4600,4700] * Web	-.356	.231	-1.542	.123	-.809	.096
(4700,4800] * Web	-.345	.232	-1.485	.138	-.800	.110
(4800,4900] * Web	-.297	.234	-1.271	.204	-.756	.161
(4900,5000] * Web	-.287	.235	-1.222	.222	-.748	.174
(5000,5100] * Web	-.192	.237	-.808	.419	-.657	.274
(5100,5200] * Web	-.344	.241	-1.430	.153	-.816	.128
(5200,5300] * Web	-.210	.243	-.861	.389	-.687	.267

(5300,5400] * Web	-.253	.248	-1.017	.309	-.740	.234
(5400,5500] * Web	-.348	.254	-1.373	.170	-.845	.149
(5500,5600] * Web	-.307	.261	-1.174	.240	-.819	.205
(5600,5700] * Web	-.209	.264	-.791	.429	-.725	.308
(5700,5800] * Web	-.129	.268	-.482	.630	-.654	.396
(5800,5900] * Web	-.076	.277	-.274	.784	-.618	.467
(5900,6000] * Web	-.186	.282	-.661	.509	-.740	.367
(6000,6100] * Web	.083	.292	.283	.777	-.491	.656
(6100,6200] * Web	.035	.290	.120	.905	-.535	.604
(6200,6300] * Web	-.065	.299	-.218	.827	-.652	.522
(6300,6400] * Web	.041	.303	.137	.891	-.553	.635
(6400,6500] * Web	-.208	.304	-.682	.495	-.804	.389
(6500,6600] * Web	-.347	.313	-1.107	.268	-.961	.267
(6600,6700] * Web	-.412	.319	-1.292	.196	-1.038	.213
(6700,6800] * Web	-.312	.330	-.946	.344	-.958	.334
(6800,6900] * Web	-.415	.338	-1.226	.220	-1.078	.248
(6900,7000] * Web	-.446	.354	-1.260	.208	-1.139	.248
(7000,7100] * Web	-.400	.370	-1.082	.279	-1.125	.325
(7100,7200] * Web	-.911	.388	-2.346	.019	-1.673	-.150
(7200,7300] * Web	-.944	.399	-2.366	.018	-1.725	-.162
(7300,7400] * Web	-.585	.376	-1.557	.119	-1.321	.151
(7400,7500] * Web	-.455	.367	-1.240	.215	-1.174	.264
(7500,7600] * Web	-.650	.391	-1.663	.096	-1.417	.116
(7600,7700] * Web	-.750	.423	-1.771	.076	-1.580	.080
(7700,7800] * Web	-.726	.441	-1.645	.100	-1.591	.139
(7800,7900] * Web	-.640	.447	-1.430	.153	-1.516	.237
Locative * Web	.118	.256	.461	.645	-.384	.621
Passive * Web	-.225	.255	-.881	.378	-.725	.276
(100,200] * Web * Locative	.129	.352	.367	.713	-.561	.820
(200,300] * Web * Locative	-.001	.348	-.003	.997	-.683	.681
(300,400] * Web * Locative	.330	.346	.953	.341	-.349	1.008
(400,500] * Web * Locative	.139	.346	.401	.689	-.539	.816
(500,600] * Web * Locative	-.123	.346	-.357	.721	-.801	.554
(600,700] * Web * Locative	-.081	.348	-.232	.817	-.762	.601
(700,800] * Web * Locative	-.258	.348	-.740	.459	-.939	.424
(800,900] * Web * Locative	-.150	.348	-.431	.666	-.832	.532
(900,1000] * Web * Locative	-.423	.349	-1.211	.226	-1.107	.261
(1000,1100] * Web * Locative	-.313	.348	-.900	.368	-.994	.368
(1100,1200] * Web * Locative	-.509	.348	-1.464	.143	-1.191	.172
(1200,1300] * Web * Locative	-.472	.347	-1.358	.174	-1.152	.209
(1300,1400] * Web * Locative	-.496	.348	-1.428	.153	-1.178	.185
(1400,1500] * Web * Locative	-.421	.349	-1.205	.228	-1.106	.264
(1500,1600] * Web * Locative	-.515	.349	-1.475	.140	-1.200	.169
(1600,1700] * Web * Locative	-.333	.348	-.956	.339	-1.016	.350
(1700,1800] * Web * Locative	-.357	.347	-1.029	.304	-1.036	.323
(1800,1900] * Web * Locative	-.442	.348	-1.273	.203	-1.124	.239
(1900,2000] * Web * Locative	-.552	.347	-1.588	.112	-1.232	.129
(2000,2100] * Web * Locative	-.639	.348	-1.838	.066	-1.320	.042
(2100,2200] * Web * Locative	-.579	.347	-1.672	.095	-1.259	.100
(2200,2300] * Web * Locative	-.581	.347	-1.675	.094	-1.261	.099
(2300,2400] * Web * Locative	-.665	.348	-1.910	.056	-1.347	.017
(2400,2500] * Web * Locative	-.462	.347	-1.332	.183	-1.143	.218
(2500,2600] * Web * Locative	-.522	.347	-1.503	.133	-1.203	.159

(2600,2700] * Web * Locative	-.304	.347	-.879	.380	-.984	.375
(2700,2800] * Web * Locative	.122	.347	.351	.725	-.558	.801
(2800,2900] * Web * Locative	-.041	.346	-.118	.906	-.720	.638
(2900,3000] * Web * Locative	.026	.346	.075	.940	-.653	.705
(3000,3100] * Web * Locative	.042	.346	.120	.904	-.637	.720
(3100,3200] * Web * Locative	-.018	.347	-.051	.960	-.698	.663
(3200,3300] * Web * Locative	-.092	.348	-.266	.790	-.774	.589
(3300,3400] * Web * Locative	-.346	.348	-.995	.320	-1.028	.336
(3400,3500] * Web * Locative	-.416	.348	-1.194	.232	-1.099	.267
(3500,3600] * Web * Locative	-.408	.350	-1.165	.244	-1.094	.278
(3600,3700] * Web * Locative	-.508	.349	-1.454	.146	-1.193	.177
(3700,3800] * Web * Locative	-.415	.349	-1.187	.235	-1.100	.270
(3800,3900] * Web * Locative	-.386	.350	-1.103	.270	-1.072	.300
(3900,4000] * Web * Locative	-.499	.348	-1.432	.152	-1.181	.184
(4000,4100] * Web * Locative	-.392	.348	-1.127	.260	-1.074	.290
(4100,4200] * Web * Locative	-.681	.349	-1.953	.051	-1.364	.002
(4200,4300] * Web * Locative	-.583	.348	-1.675	.094	-1.266	.099
(4300,4400] * Web * Locative	-.522	.347	-1.503	.133	-1.203	.159
(4400,4500] * Web * Locative	-.623	.347	-1.798	.072	-1.303	.056
(4500,4600] * Web * Locative	-.858	.347	-2.470	.014	-1.538	-.177
(4600,4700] * Web * Locative	-1.096	.347	-3.155	.002	-1.777	-.415
(4700,4800] * Web * Locative	-1.115	.348	-3.204	.001	-1.797	-.433
(4800,4900] * Web * Locative	-1.155	.349	-3.307	.001	-1.839	-.470
(4900,5000] * Web * Locative	-1.217	.350	-3.477	.001	-1.904	-.531
(5000,5100] * Web * Locative	-1.367	.352	-3.883	< .001	-2.057	-.677
(5100,5200] * Web * Locative	-1.073	.354	-3.035	.002	-1.766	-.380
(5200,5300] * Web * Locative	-1.095	.356	-3.079	.002	-1.791	-.398
(5300,5400] * Web * Locative	-1.116	.361	-3.092	.002	-1.824	-.409
(5400,5500] * Web * Locative	-.882	.366	-2.411	.016	-1.598	-.165
(5500,5600] * Web * Locative	-.800	.372	-2.153	.031	-1.529	-.072
(5600,5700] * Web * Locative	-.908	.376	-2.413	.016	-1.645	-.170
(5700,5800] * Web * Locative	-.893	.380	-2.352	.019	-1.637	-.149
(5800,5900] * Web * Locative	-.803	.392	-2.050	.040	-1.571	-.035
(5900,6000] * Web * Locative	-.522	.397	-1.316	.188	-1.300	.256
(6000,6100] * Web * Locative	-.885	.410	-2.157	.031	-1.690	-.081
(6100,6200] * Web * Locative	-.844	.417	-2.024	.043	-1.661	-.027
(6200,6300] * Web * Locative	-.472	.422	-1.117	.264	-1.300	.356
(6300,6400] * Web * Locative	-.548	.431	-1.271	.204	-1.394	.297
(6400,6500] * Web * Locative	-.305	.437	-.699	.485	-1.161	.551
(6500,6600] * Web * Locative	-.311	.447	-.695	.487	-1.188	.566
(6600,6700] * Web * Locative	-.070	.452	-.155	.877	-.955	.816
(6700,6800] * Web * Locative	-.287	.468	-.613	.540	-1.204	.630
(6800,6900] * Web * Locative	-.200	.474	-.423	.672	-1.129	.728
(6900,7000] * Web * Locative	-.292	.503	-.580	.562	-1.277	.694
(7000,7100] * Web * Locative	.122	.524	.232	.816	-.905	1.148
(7100,7200] * Web * Locative	.825	.545	1.514	.130	-.243	1.892
(7200,7300] * Web * Locative	.847	.579	1.461	.144	-.289	1.983
(7300,7400] * Web * Locative	.828	.581	1.426	.154	-.310	1.967
(7400,7500] * Web * Locative	.537	.583	.921	.357	-.606	1.680
(7500,7600] * Web * Locative	1.004	.609	1.649	.099	-.189	2.197
(7600,7700] * Web * Locative	.495	.634	.780	.436	-.749	1.738
(7700,7800] * Web * Locative	.252	.666	.379	.705	-1.053	1.557
(7800,7900] * Web * Locative	.074	.685	.108	.914	-1.269	1.417

(100,200] * Web * Passive	.160	.352	.454	.650	-.530	.850
(200,300] * Web * Passive	-.059	.349	-.168	.867	-.743	.626
(300,400] * Web * Passive	.009	.350	.026	.980	-.676	.694
(400,500] * Web * Passive	-.139	.350	-.397	.692	-.824	.547
(500,600] * Web * Passive	-.280	.351	-.797	.426	-.967	.408
(600,700] * Web * Passive	-.227	.351	-.646	.518	-.914	.460
(700,800] * Web * Passive	-.351	.351	-.999	.318	-1.040	.338
(800,900] * Web * Passive	.008	.349	.023	.981	-.676	.693
(900,1000] * Web * Passive	.182	.349	.522	.602	-.501	.865
(1000,1100] * Web * Passive	.183	.348	.526	.599	-.499	.865
(1100,1200] * Web * Passive	-.055	.349	-.158	.875	-.738	.628
(1200,1300] * Web * Passive	.116	.346	.334	.738	-.563	.794
(1300,1400] * Web * Passive	.142	.346	.411	.681	-.536	.820
(1400,1500] * Web * Passive	.183	.348	.527	.598	-.499	.865
(1500,1600] * Web * Passive	.116	.347	.333	.739	-.564	.795
(1600,1700] * Web * Passive	.302	.346	.872	.383	-.377	.981
(1700,1800] * Web * Passive	.078	.346	.225	.822	-.600	.756
(1800,1900] * Web * Passive	-.024	.347	-.069	.945	-.703	.655
(1900,2000] * Web * Passive	-.025	.346	-.073	.942	-.703	.652
(2000,2100] * Web * Passive	-.150	.346	-.434	.664	-.827	.527
(2100,2200] * Web * Passive	-.067	.346	-.194	.846	-.745	.611
(2200,2300] * Web * Passive	-.093	.346	-.269	.788	-.772	.585
(2300,2400] * Web * Passive	-.284	.348	-.817	.414	-.965	.397
(2400,2500] * Web * Passive	-.106	.347	-.306	.760	-.786	.574
(2500,2600] * Web * Passive	-.041	.346	-.119	.905	-.720	.638
(2600,2700] * Web * Passive	.130	.347	.374	.708	-.549	.809
(2700,2800] * Web * Passive	.287	.348	.825	.409	-.395	.969
(2800,2900] * Web * Passive	.123	.348	.354	.724	-.559	.805
(2900,3000] * Web * Passive	.194	.348	.558	.577	-.487	.875
(3000,3100] * Web * Passive	.302	.347	.870	.384	-.378	.982
(3100,3200] * Web * Passive	.286	.348	.822	.411	-.396	.969
(3200,3300] * Web * Passive	.261	.348	.751	.453	-.421	.944
(3300,3400] * Web * Passive	-.110	.349	-.314	.754	-.794	.575
(3400,3500] * Web * Passive	-.247	.350	-.704	.481	-.933	.440
(3500,3600] * Web * Passive	.071	.349	.204	.838	-.614	.756
(3600,3700] * Web * Passive	.001	.349	.002	.998	-.684	.685
(3700,3800] * Web * Passive	.174	.349	.499	.618	-.510	.859
(3800,3900] * Web * Passive	.338	.348	.969	.333	-.345	1.020
(3900,4000] * Web * Passive	.208	.347	.600	.549	-.472	.888
(4000,4100] * Web * Passive	.571	.346	1.649	.099	-.108	1.250
(4100,4200] * Web * Passive	.327	.346	.946	.344	-.351	1.005
(4200,4300] * Web * Passive	.614	.345	1.778	.075	-.063	1.290
(4300,4400] * Web * Passive	.809	.344	2.348	.019	.134	1.484
(4400,4500] * Web * Passive	.661	.344	1.918	.055	-.014	1.335
(4500,4600] * Web * Passive	.619	.345	1.792	.073	-.058	1.296
(4600,4700] * Web * Passive	.433	.347	1.247	.212	-.247	1.113
(4700,4800] * Web * Passive	.478	.349	1.369	.171	-.207	1.163
(4800,4900] * Web * Passive	.364	.354	1.028	.304	-.330	1.059
(4900,5000] * Web * Passive	.457	.356	1.281	.200	-.242	1.155
(5000,5100] * Web * Passive	.315	.363	.866	.387	-.398	1.027
(5100,5200] * Web * Passive	.449	.370	1.214	.225	-.276	1.174
(5200,5300] * Web * Passive	.575	.371	1.551	.121	-.152	1.301
(5300,5400] * Web * Passive	.346	.377	.918	.358	-.392	1.085

(5400,5500] * Web * Passive	.329	.382	.862	.388	-.419	1.077
(5500,5600] * Web * Passive	.124	.394	.315	.753	-.648	.896
(5600,5700] * Web * Passive	.160	.397	.402	.688	-.618	.938
(5700,5800] * Web * Passive	.168	.397	.423	.672	-.610	.946
(5800,5900] * Web * Passive	.135	.409	.331	.741	-.665	.936
(5900,6000] * Web * Passive	.354	.416	.852	.394	-.461	1.170
(6000,6100] * Web * Passive	-.017	.431	-.040	.968	-.862	.828
(6100,6200] * Web * Passive	.035	.437	.080	.937	-.821	.891
(6200,6300] * Web * Passive	.191	.447	.427	.670	-.685	1.067
(6300,6400] * Web * Passive	.248	.455	.545	.586	-.643	1.139
(6400,6500] * Web * Passive	.472	.464	1.017	.309	-.438	1.382
(6500,6600] * Web * Passive	.623	.478	1.303	.193	-.314	1.559
(6600,6700] * Web * Passive	.723	.476	1.519	.129	-.210	1.655
(6700,6800] * Web * Passive	.592	.493	1.200	.230	-.375	1.559
(6800,6900] * Web * Passive	.830	.521	1.594	.111	-.191	1.851
(6900,7000] * Web * Passive	1.300	.523	2.486	.013	.275	2.324
(7000,7100] * Web * Passive	1.181	.536	2.203	.028	.130	2.232
(7100,7200] * Web * Passive	2.089	.553	3.778	< .001	1.005	3.172
(7200,7300] * Web * Passive	2.210	.574	3.848	< .001	1.084	3.336
(7300,7400] * Web * Passive	1.721	.563	3.054	.002	.617	2.825
(7400,7500] * Web * Passive	1.169	.551	2.122	.034	.089	2.248
(7500,7600] * Web * Passive	.903	.576	1.568	.117	-.226	2.031
(7600,7700] * Web * Passive	.685	.623	1.100	.271	-.536	1.906
(7700,7800] * Web * Passive	1.035	.627	1.650	.099	-.195	2.264
(7800,7900] * Web * Passive	.988	.639	1.546	.122	-.264	2.240

Model D

*Target Proportions ~ Bin * Type * Mode* (PWA data only; quasiomial family). Model R^2 = .215. Reference level for Bin was (0,100], reference level for Mode was Lab, reference level for Type was Active.

	Est	SE	*t*	*p*	Lower CI	Upper CI
(Intercept)	-1.412	.203			-1.809	-1.014
(100,200]	.652	.266	2.448	.014	.130	1.174
(200,300]	.952	.262	3.640	< .001	.440	1.465
(300,400]	1.095	.260	4.209	< .001	.585	1.605
(400,500]	1.000	.261	3.830	< .001	.488	1.512
(500,600]	1.003	.261	3.840	< .001	.491	1.514
(600,700]	1.095	.260	4.206	.000	.585	1.605
(700,800]	1.137	.260	4.375	.000	.628	1.647
(800,900]	1.262	.259	4.868	< .001	.754	1.770
(900,1000]	1.327	.259	5.124	< .001	.820	1.835
(1000,1100]	1.133	.260	4.359	< .001	.624	1.643
(1100,1200]	1.155	.260	4.446	< .001	.646	1.664
(1200,1300]	1.184	.260	4.558	< .001	.675	1.692
(1300,1400]	1.283	.259	4.951	< .001	.775	1.791
(1400,1500]	1.233	.259	4.753	< .001	.724	1.741
(1500,1600]	1.176	.260	4.530	< .001	.667	1.685
(1600,1700]	1.201	.260	4.628	< .001	.693	1.710
(1700,1800]	1.205	.260	4.642	< .001	.696	1.713
(1800,1900]	1.246	.259	4.805	< .001	.738	1.754
(1900,2000]	1.238	.259	4.772	< .001	.729	1.746

(2000,2100]	1.216	.259	4.685	< .001	.707	1.724
(2100,2200]	1.151	.260	4.431	< .001	.642	1.661
(2200,2300]	1.134	.260	4.364	< .001	.625	1.644
(2300,2400]	1.216	.259	4.688	< .001	.708	1.725
(2400,2500]	1.258	.259	4.850	< .001	.749	1.766
(2500,2600]	1.179	.260	4.542	< .001	.670	1.688
(2600,2700]	1.229	.259	4.738	< .001	.721	1.737
(2700,2800]	1.350	.259	5.210	< .001	.842	1.857
(2800,2900]	1.314	.259	5.069	< .001	.806	1.821
(2900,3000]	1.351	.259	5.216	< .001	.843	1.859
(3000,3100]	1.467	.259	5.663	< .001	.959	1.975
(3100,3200]	1.552	.259	5.987	< .001	1.044	2.060
(3200,3300]	1.516	.259	5.850	< .001	1.008	2.024
(3300,3400]	1.493	.259	5.762	< .001	.985	2.000
(3400,3500]	1.542	.259	5.948	< .001	1.034	2.050
(3500,3600]	1.621	.260	6.246	< .001	1.113	2.130
(3600,3700]	1.703	.260	6.549	< .001	1.193	2.213
(3700,3800]	1.720	.260	6.611	< .001	1.210	2.230
(3800,3900]	1.830	.261	7.007	< .001	1.318	2.342
(3900,4000]	1.803	.261	6.910	< .001	1.292	2.314
(4000,4100]	1.765	.261	6.775	< .001	1.255	2.276
(4100,4200]	1.752	.260	6.729	< .001	1.242	2.263
(4200,4300]	1.772	.261	6.801	< .001	1.262	2.283
(4300,4400]	1.779	.261	6.826	< .001	1.268	2.290
(4400,4500]	1.785	.261	6.847	< .001	1.274	2.296
(4500,4600]	1.574	.259	6.070	< .001	1.066	2.082
(4600,4700]	1.547	.259	5.969	< .001	1.039	2.055
(4700,4800]	1.532	.259	5.911	< .001	1.024	2.040
(4800,4900]	1.551	.259	5.982	< .001	1.043	2.059
(4900,5000]	1.464	.259	5.653	< .001	.957	1.972
(5000,5100]	1.306	.259	5.038	< .001	.798	1.813
(5100,5200]	1.227	.259	4.729	< .001	.718	1.735
(5200,5300]	1.087	.260	4.176	< .001	.577	1.597
(5300,5400]	.934	.262	3.566	< .001	.421	1.447
(5400,5500]	.871	.263	3.316	.001	.356	1.386
(5500,5600]	.736	.265	2.781	.005	.217	1.256
(5600,5700]	.681	.266	2.560	.010	.160	1.202
(5700,5800]	.633	.267	2.372	.018	.110	1.156
(5800,5900]	.420	.272	1.546	.122	-.113	.954
(5900,6000]	.368	.273	1.347	.178	-.168	.904
(6000,6100]	.113	.282	.401	.688	-.440	.666
(6100,6200]	.171	.280	.612	.540	-.377	.720
(6200,6300]	.081	.283	.285	.776	-.475	.636
(6300,6400]	.012	.286	.041	.967	-.550	.573
(6400,6500]	.128	.282	.453	.651	-.424	.680
(6500,6600]	.090	.283	.318	.751	-.465	.645
(6600,6700]	.055	.285	.194	.847	-.503	.613
(6700,6800]	-.133	.293	-.453	.651	-.707	.442
(6800,6900]	-.166	.295	-.564	.573	-.744	.411
(6900,7000]	-.302	.302	-1.001	.317	-.894	.290
(7000,7100]	-.481	.313	-1.535	.125	-1.095	.133
(7100,7200]	-.288	.301	-.955	.340	-.878	.303
(7200,7300]	-.346	.305	-1.135	.256	-.943	.251

(7300,7400]	-.358	.305	-1.173	.241	-.957	.240
(7400,7500]	-.365	.306	-1.193	.233	-.964	.235
(7500,7600]	-.439	.310	-1.415	.157	-1.048	.169
(7600,7700]	-.602	.322	-1.871	.061	-1.233	.029
(7700,7800]	-.733	.332	-2.208	.027	-1.384	-.082
(7800,7900]	-.825	.340	-2.426	.015	-1.491	-.159
Locative	-.070	.288	-.244	.807	-.634	.494
Passive	.267	.286	.933	.351	-.294	.828
Web	.447	.274	1.629	.103	-.091	.984
(100,200] * Locative	-.079	.379	-.210	.834	-.821	.663
(200,300] * Locative	.160	.369	.434	.665	-.563	.883
(300,400] * Locative	.083	.367	.226	.821	-.637	.803
(400,500] * Locative	.183	.368	.498	.618	-.538	.905
(500,600] * Locative	.247	.368	.671	.502	-.474	.967
(600,700] * Locative	-.141	.369	-.383	.702	-.865	.582
(700,800] * Locative	.001	.367	.003	.998	-.719	.721
(800,900] * Locative	-.104	.367	-.285	.776	-.824	.615
(900,1000] * Locative	-.136	.367	-.371	.711	-.854	.582
(1000,1100] * Locative	.174	.367	.475	.635	-.544	.893
(1100,1200] * Locative	.247	.366	.674	.500	-.471	.965
(1200,1300] * Locative	.179	.366	.489	.625	-.539	.897
(1300,1400] * Locative	.111	.366	.303	.762	-.607	.828
(1400,1500] * Locative	.107	.366	.293	.770	-.610	.825
(1500,1600] * Locative	.183	.366	.500	.617	-.535	.901
(1600,1700] * Locative	.249	.366	.679	.497	-.469	.966
(1700,1800] * Locative	.291	.366	.796	.426	-.426	1.009
(1800,1900] * Locative	.203	.366	.555	.579	-.514	.921
(1900,2000] * Locative	.269	.366	.736	.462	-.448	.987
(2000,2100] * Locative	.165	.366	.449	.653	-.553	.882
(2100,2200] * Locative	.250	.366	.681	.496	-.469	.968
(2200,2300] * Locative	.293	.366	.798	.425	-.426	1.011
(2300,2400] * Locative	.256	.366	.700	.484	-.461	.974
(2400,2500] * Locative	.215	.366	.588	.557	-.502	.933
(2500,2600] * Locative	.413	.366	1.126	.260	-.306	1.131
(2600,2700] * Locative	.392	.366	1.070	.285	-.326	1.110
(2700,2800] * Locative	.017	.366	.047	.963	-.700	.734
(2800,2900] * Locative	.048	.366	.133	.895	-.669	.766
(2900,3000] * Locative	.029	.366	.078	.937	-.688	.746
(3000,3100] * Locative	.076	.366	.208	.835	-.641	.793
(3100,3200] * Locative	.221	.367	.603	.546	-.498	.940
(3200,3300] * Locative	.527	.369	1.429	.153	-.196	1.249
(3300,3400] * Locative	.592	.369	1.606	.108	-.131	1.316
(3400,3500] * Locative	.636	.370	1.719	.086	-.089	1.362
(3500,3600] * Locative	.457	.369	1.237	.216	-.267	1.180
(3600,3700] * Locative	.426	.370	1.150	.250	-.300	1.151
(3700,3800] * Locative	.405	.370	1.095	.274	-.320	1.131
(3800,3900] * Locative	.172	.370	.466	.641	-.552	.897
(3900,4000] * Locative	.078	.369	.210	.833	-.645	.800
(4000,4100] * Locative	.104	.368	.283	.778	-.618	.826
(4100,4200] * Locative	.009	.367	.024	.981	-.711	.729
(4200,4300] * Locative	-.019	.368	-.053	.958	-.740	.701
(4300,4400] * Locative	-.011	.368	-.029	.977	-.731	.710
(4400,4500] * Locative	-.116	.367	-.316	.752	-.836	.604

(4500,4600] * Locative	.172	.367	.469	.639	-.547	.890
(4600,4700] * Locative	.141	.366	.385	.700	-.577	.859
(4700,4800] * Locative	.148	.366	.404	.686	-.570	.866
(4800,4900] * Locative	.090	.366	.246	.805	-.627	.808
(4900,5000] * Locative	.272	.366	.742	.458	-.446	.990
(5000,5100] * Locative	.393	.366	1.073	.283	-.325	1.111
(5100,5200] * Locative	.406	.366	1.108	.268	-.312	1.124
(5200,5300] * Locative	.436	.367	1.188	.235	-.283	1.155
(5300,5400] * Locative	.523	.368	1.423	.155	-.197	1.244
(5400,5500] * Locative	.572	.368	1.554	.120	-.150	1.294
(5500,5600] * Locative	.665	.370	1.797	.072	-.060	1.390
(5600,5700] * Locative	.716	.371	1.931	.053	-.011	1.443
(5700,5800] * Locative	.729	.371	1.963	.050	.001	1.457
(5800,5900] * Locative	.705	.376	1.875	.061	-.032	1.442
(5900,6000] * Locative	.657	.378	1.738	.082	-.084	1.398
(6000,6100] * Locative	.776	.386	2.013	.044	.020	1.532
(6100,6200] * Locative	.584	.385	1.515	.130	-.171	1.340
(6200,6300] * Locative	.653	.388	1.683	.092	-.107	1.414
(6300,6400] * Locative	.603	.392	1.539	.124	-.165	1.372
(6400,6500] * Locative	.431	.390	1.106	.269	-.333	1.194
(6500,6600] * Locative	.476	.391	1.220	.222	-.289	1.242
(6600,6700] * Locative	.403	.394	1.023	.306	-.369	1.174
(6700,6800] * Locative	.532	.401	1.328	.184	-.253	1.318
(6800,6900] * Locative	.586	.402	1.459	.145	-.201	1.373
(6900,7000] * Locative	.498	.412	1.210	.226	-.309	1.306
(7000,7100] * Locative	.231	.435	.531	.595	-.621	1.083
(7100,7200] * Locative	-.170	.435	-.390	.697	-1.023	.683
(7200,7300] * Locative	-.410	.454	-.902	.367	-1.301	.481
(7300,7400] * Locative	-.716	.478	-1.497	.134	-1.653	.222
(7400,7500] * Locative	-.700	.478	-1.465	.143	-1.636	.236
(7500,7600] * Locative	-.825	.499	-1.655	.098	-1.803	.152
(7600,7700] * Locative	-.397	.483	-.823	.411	-1.344	.549
(7700,7800] * Locative	-.281	.491	-.572	.567	-1.243	.681
(7800,7900] * Locative	-.237	.500	-.474	.636	-1.217	.743
(100,200] * Passive	-.357	.384	-.929	.353	-1.109	.396
(200,300] * Passive	-.016	.373	-.044	.965	-.748	.715
(300,400] * Passive	-.164	.372	-.441	.659	-.894	.566
(400,500] * Passive	-.051	.373	-.137	.891	-.782	.680
(500,600] * Passive	-.205	.374	-.549	.583	-.938	.527
(600,700] * Passive	-.218	.373	-.585	.559	-.948	.512
(700,800] * Passive	-.299	.373	-.802	.423	-1.029	.432
(800,900] * Passive	-.603	.374	-1.615	.106	-1.336	.129
(900,1000] * Passive	-.665	.374	-1.780	.075	-1.397	.067
(1000,1100] * Passive	-.293	.373	-.785	.432	-1.023	.438
(1100,1200] * Passive	-.250	.372	-.672	.502	-.980	.479
(1200,1300] * Passive	-.104	.372	-.279	.781	-.832	.625
(1300,1400] * Passive	-.115	.371	-.310	.757	-.843	.613
(1400,1500] * Passive	-.086	.371	-.231	.817	-.814	.642
(1500,1600] * Passive	.163	.372	.438	.661	-.566	.892
(1600,1700] * Passive	.320	.373	.857	.391	-.411	1.050
(1700,1800] * Passive	.416	.374	1.114	.265	-.316	1.149
(1800,1900] * Passive	.345	.373	.925	.355	-.387	1.077
(1900,2000] * Passive	.239	.372	.643	.520	-.491	.969

(2000,2100] * Passive	.120	.372	.323	.746	-.608	.849
(2100,2200] * Passive	.194	.372	.520	.603	-.536	.923
(2200,2300] * Passive	.331	.373	.887	.375	-.400	1.061
(2300,2400] * Passive	.145	.372	.389	.697	-.584	.874
(2400,2500] * Passive	-.035	.371	-.095	.924	-.763	.693
(2500,2600] * Passive	.035	.372	.094	.925	-.694	.763
(2600,2700] * Passive	-.163	.371	-.438	.661	-.891	.565
(2700,2800] * Passive	-.147	.371	-.395	.693	-.874	.581
(2800,2900] * Passive	-.150	.371	-.405	.685	-.878	.577
(2900,3000] * Passive	-.164	.371	-.442	.659	-.891	.563
(3000,3100] * Passive	-.238	.371	-.642	.521	-.966	.489
(3100,3200] * Passive	-.151	.372	-.406	.685	-.880	.578
(3200,3300] * Passive	-.194	.371	-.521	.602	-.922	.534
(3300,3400] * Passive	-.307	.371	-.828	.407	-1.035	.420
(3400,3500] * Passive	-.352	.371	-.948	.343	-1.079	.376
(3500,3600] * Passive	-.411	.372	-1.106	.269	-1.139	.317
(3600,3700] * Passive	-.357	.372	-.959	.338	-1.087	.373
(3700,3800] * Passive	-.452	.372	-1.215	.224	-1.181	.277
(3800,3900] * Passive	-.696	.373	-1.869	.062	-1.427	.034
(3900,4000] * Passive	-.594	.372	-1.595	.111	-1.324	.136
(4000,4100] * Passive	-.503	.372	-1.351	.177	-1.233	.227
(4100,4200] * Passive	-.542	.372	-1.457	.145	-1.272	.187
(4200,4300] * Passive	-.591	.372	-1.588	.112	-1.321	.138
(4300,4400] * Passive	-.594	.372	-1.595	.111	-1.323	.136
(4400,4500] * Passive	-.707	.372	-1.898	.058	-1.437	.023
(4500,4600] * Passive	-.486	.371	-1.309	.191	-1.214	.242
(4600,4700] * Passive	-.572	.372	-1.539	.124	-1.300	.156
(4700,4800] * Passive	-.644	.372	-1.732	.083	-1.373	.085
(4800,4900] * Passive	-.807	.373	-2.165	.030	-1.538	-.076
(4900,5000] * Passive	-.763	.373	-2.044	.041	-1.494	-.031
(5000,5100] * Passive	-.810	.376	-2.156	.031	-1.546	-.074
(5100,5200] * Passive	-.792	.377	-2.102	.036	-1.530	-.054
(5200,5300] * Passive	-.627	.377	-1.663	.096	-1.365	.112
(5300,5400] * Passive	-.372	.377	-.989	.323	-1.110	.366
(5400,5500] * Passive	-.256	.377	-.680	.497	-.994	.482
(5500,5600] * Passive	-.083	.378	-.219	.826	-.823	.657
(5600,5700] * Passive	-.113	.379	-.298	.766	-.856	.630
(5700,5800] * Passive	-.039	.380	-.102	.919	-.783	.706
(5800,5900] * Passive	.065	.385	.169	.866	-.689	.819
(5900,6000] * Passive	-.080	.389	-.205	.837	-.842	.682
(6000,6100] * Passive	.106	.396	.269	.788	-.670	.883
(6100,6200] * Passive	-.150	.399	-.375	.708	-.932	.633
(6200,6300] * Passive	-.060	.402	-.150	.881	-.848	.727
(6300,6400] * Passive	-.206	.410	-.503	.615	-1.010	.597
(6400,6500] * Passive	-.483	.412	-1.170	.242	-1.291	.326
(6500,6600] * Passive	-.576	.419	-1.376	.169	-1.397	.245
(6600,6700] * Passive	-.414	.415	-.999	.318	-1.227	.398
(6700,6800] * Passive	-.364	.426	-.855	.393	-1.199	.471
(6800,6900] * Passive	-.611	.441	-1.384	.166	-1.475	.254
(6900,7000] * Passive	-.582	.452	-1.287	.198	-1.469	.305
(7000,7100] * Passive	-.400	.460	-.871	.384	-1.301	.501
(7100,7200] * Passive	-.782	.464	-1.684	.092	-1.692	.128
(7200,7300] * Passive	-.907	.481	-1.886	.059	-1.850	.035

(7300,7400] * Passive	-.901	.482	-1.870	.061	-1.846	.043
(7400,7500] * Passive	-.670	.465	-1.442	.149	-1.581	.241
(7500,7600] * Passive	-.453	.459	-.988	.323	-1.352	.446
(7600,7700] * Passive	-.331	.469	-.706	.480	-1.250	.588
(7700,7800] * Passive	-.349	.486	-.718	.473	-1.301	.603
(7800,7900] * Passive	-.345	.497	-.693	.488	-1.320	.630
(100,200] * Web	-.150	.366	-.411	.681	-.867	.566
(200,300] * Web	-.509	.363	-1.404	.160	-1.220	.202
(300,400] * Web	-.742	.363	-2.045	.041	-1.453	-.031
(400,500] * Web	-.677	.364	-1.861	.063	-1.390	.036
(500,600] * Web	-.731	.364	-2.006	.045	-1.445	-.017
(600,700] * Web	-.961	.366	-2.625	.009	-1.678	-.243
(700,800] * Web	-.939	.365	-2.574	.010	-1.653	-.224
(800,900] * Web	-.970	.363	-2.673	.008	-1.681	-.259
(900,1000] * Web	-1.276	.367	-3.481	.000	-1.995	-.558
(1000,1100] * Web	-1.032	.366	-2.817	.005	-1.750	-.314
(1100,1200] * Web	-.832	.363	-2.293	.022	-1.543	-.121
(1200,1300] * Web	-.891	.363	-2.454	.014	-1.603	-.179
(1300,1400] * Web	-.870	.361	-2.407	.016	-1.578	-.162
(1400,1500] * Web	-1.132	.366	-3.092	.002	-1.849	-.414
(1500,1600] * Web	-.884	.363	-2.434	.015	-1.595	-.172
(1600,1700] * Web	-.955	.364	-2.627	.009	-1.668	-.243
(1700,1800] * Web	-.897	.363	-2.472	.013	-1.608	-.186
(1800,1900] * Web	-.923	.362	-2.547	.011	-1.633	-.213
(1900,2000] * Web	-.839	.362	-2.321	.020	-1.548	-.130
(2000,2100] * Web	-.743	.361	-2.059	.040	-1.450	-.036
(2100,2200] * Web	-1.083	.367	-2.953	.003	-1.802	-.364
(2200,2300] * Web	-1.050	.367	-2.863	.004	-1.768	-.331
(2300,2400] * Web	-1.115	.366	-3.047	.002	-1.832	-.398
(2400,2500] * Web	-1.043	.364	-2.866	.004	-1.756	-.330
(2500,2600] * Web	-.949	.364	-2.608	.009	-1.662	-.236
(2600,2700] * Web	-.999	.364	-2.746	.006	-1.712	-.286
(2700,2800] * Web	-1.135	.364	-3.121	.002	-1.848	-.422
(2800,2900] * Web	-1.052	.363	-2.897	.004	-1.764	-.340
(2900,3000] * Web	-.998	.362	-2.757	.006	-1.707	-.288
(3000,3100] * Web	-1.038	.361	-2.877	.004	-1.746	-.331
(3100,3200] * Web	-1.418	.365	-3.883	< .001	-2.134	-.702
(3200,3300] * Web	-1.239	.363	-3.413	.001	-1.950	-.527
(3300,3400] * Web	-1.200	.363	-3.309	.001	-1.911	-.489
(3400,3500] * Web	-1.158	.362	-3.202	.001	-1.867	-.449
(3500,3600] * Web	-1.178	.361	-3.261	.001	-1.886	-.470
(3600,3700] * Web	-1.290	.362	-3.564	< .001	-1.999	-.580
(3700,3800] * Web	-1.336	.362	-3.689	< .001	-2.047	-.626
(3800,3900] * Web	-1.538	.364	-4.223	< .001	-2.252	-.824
(3900,4000] * Web	-1.480	.364	-4.070	< .001	-2.192	-.767
(4000,4100] * Web	-1.458	.364	-4.010	< .001	-2.170	-.745
(4100,4200] * Web	-1.554	.365	-4.257	< .001	-2.269	-.838
(4200,4300] * Web	-1.671	.367	-4.556	< .001	-2.390	-.952
(4300,4400] * Web	-1.779	.369	-4.825	< .001	-2.502	-1.057
(4400,4500] * Web	-1.734	.368	-4.715	< .001	-2.455	-1.013
(4500,4600] * Web	-1.591	.368	-4.323	< .001	-2.313	-.870
(4600,4700] * Web	-1.513	.367	-4.122	< .001	-2.233	-.794
(4700,4800] * Web	-1.382	.365	-3.786	< .001	-2.097	-.666

(4800,4900] * Web	-1.289	.363	-3.549	< .001	-2.001	-.577
(4900,5000] * Web	-1.172	.363	-3.231	.001	-1.882	-.461
(5000,5100] * Web	-1.044	.363	-2.875	.004	-1.756	-.332
(5100,5200] * Web	-1.193	.367	-3.248	.001	-1.912	-.473
(5200,5300] * Web	-1.087	.369	-2.950	.003	-1.809	-.365
(5300,5400] * Web	-.951	.370	-2.571	.010	-1.676	-.226
(5400,5500] * Web	-.924	.371	-2.487	.013	-1.651	-.196
(5500,5600] * Web	-.843	.374	-2.253	.024	-1.576	-.109
(5600,5700] * Web	-.716	.373	-1.917	.055	-1.447	.016
(5700,5800] * Web	-.703	.375	-1.877	.061	-1.438	.031
(5800,5900] * Web	-.563	.380	-1.482	.138	-1.308	.182
(5900,6000] * Web	-.625	.384	-1.628	.103	-1.378	.127
(6000,6100] * Web	-.331	.389	-.851	.395	-1.094	.432
(6100,6200] * Web	-.428	.389	-1.102	.270	-1.190	.333
(6200,6300] * Web	-.524	.397	-1.320	.187	-1.301	.254
(6300,6400] * Web	-.477	.400	-1.193	.233	-1.260	.307
(6400,6500] * Web	-.780	.403	-1.935	.053	-1.570	.010
(6500,6600] * Web	-.846	.409	-2.071	.038	-1.647	-.045
(6600,6700] * Web	-.894	.413	-2.162	.031	-1.704	-.083
(6700,6800] * Web	-.735	.421	-1.746	.081	-1.559	.090
(6800,6900] * Web	-.823	.428	-1.921	.055	-1.662	.017
(6900,7000] * Web	-.820	.441	-1.859	.063	-1.684	.045
(7000,7100] * Web	-.714	.453	-1.574	.115	-1.602	.175
(7100,7200] * Web	-1.153	.463	-2.490	.013	-2.060	-.245
(7200,7300] * Web	-1.189	.473	-2.513	.012	-2.116	-.262
(7300,7400] * Web	-.836	.448	-1.867	.062	-1.714	.042
(7400,7500] * Web	-.689	.440	-1.568	.117	-1.550	.173
(7500,7600] * Web	-.913	.462	-1.976	.048	-1.819	-.007
(7600,7700] * Web	-1.035	.493	-2.099	.036	-2.002	-.068
(7700,7800] * Web	-.905	.500	-1.809	.070	-1.885	.076
(7800,7900] * Web	-.813	.505	-1.609	.108	-1.803	.177
Locative * Web	.119	.397	.300	.764	-.658	.896
Passive * Web	-.200	.397	-.503	.615	-.979	.579
(100,200] * Locative * Web	-.162	.532	-.304	.761	-1.205	.882
(200,300] * Locative * Web	-.424	.527	-.806	.420	-1.457	.608
(300,400] * Locative * Web	.053	.524	.101	.919	-.973	1.079
(400,500] * Locative * Web	.018	.524	.035	.972	-1.009	1.045
(500,600] * Locative * Web	-.136	.525	-.258	.796	-1.165	.894
(600,700] * Locative * Web	.048	.532	.090	.928	-.994	1.090
(700,800] * Locative * Web	-.416	.535	-.778	.437	-1.464	.632
(800,900] * Locative * Web	-.404	.533	-.758	.448	-1.450	.641
(900,1000] * Locative * Web	-.109	.535	-.203	.839	-1.158	.941
(1000,1100] * Locative * Web	-.235	.531	-.442	.658	-1.275	.805
(1100,1200] * Locative * Web	-.654	.530	-1.234	.217	-1.693	.385
(1200,1300] * Locative * Web	-.643	.532	-1.208	.227	-1.686	.400
(1300,1400] * Locative * Web	-.630	.530	-1.189	.234	-1.668	.408
(1400,1500] * Locative * Web	-.668	.541	-1.235	.217	-1.729	.392
(1500,1600] * Locative * Web	-.884	.538	-1.644	.100	-1.938	.170
(1600,1700] * Locative * Web	-.688	.533	-1.292	.196	-1.733	.356
(1700,1800] * Locative * Web	-.499	.527	-.946	.344	-1.532	.535
(1800,1900] * Locative * Web	-.568	.529	-1.074	.283	-1.605	.469
(1900,2000] * Locative * Web	-.668	.528	-1.266	.206	-1.702	.367
(2000,2100] * Locative * Web	-.764	.530	-1.443	.149	-1.802	.274

(2100,2200] * Locative * Web	-.197	.530	-.372	.710	-1.235	.841
(2200,2300] * Locative * Web	-.257	.529	-.485	.628	-1.295	.781
(2300,2400] * Locative * Web	-.160	.528	-.303	.762	-1.195	.875
(2400,2500] * Locative * Web	-.083	.525	-.158	.875	-1.112	.946
(2500,2600] * Locative * Web	-.333	.525	-.633	.527	-1.363	.697
(2600,2700] * Locative * Web	-.463	.527	-.878	.380	-1.496	.570
(2700,2800] * Locative * Web	-.092	.527	-.174	.862	-1.125	.942
(2800,2900] * Locative * Web	-.037	.525	-.070	.944	-1.066	.993
(2900,3000] * Locative * Web	-.301	.527	-.572	.567	-1.334	.731
(3000,3100] * Locative * Web	-.424	.526	-.805	.421	-1.455	.608
(3100,3200] * Locative * Web	-.274	.530	-.518	.604	-1.312	.764
(3200,3300] * Locative * Web	-.421	.526	-.800	.424	-1.451	.610
(3300,3400] * Locative * Web	-.612	.527	-1.161	.246	-1.645	.421
(3400,3500] * Locative * Web	-.637	.526	-1.211	.226	-1.668	.394
(3500,3600] * Locative * Web	-.645	.526	-1.226	.220	-1.677	.386
(3600,3700] * Locative * Web	-.566	.527	-1.075	.283	-1.599	.466
(3700,3800] * Locative * Web	-.708	.530	-1.337	.181	-1.746	.330
(3800,3900] * Locative * Web	-.344	.529	-.650	.516	-1.382	.693
(3900,4000] * Locative * Web	-.128	.526	-.242	.808	-1.159	.904
(4000,4100] * Locative * Web	-.102	.526	-.194	.847	-1.133	.929
(4100,4200] * Locative * Web	-.291	.532	-.548	.584	-1.334	.751
(4200,4300] * Locative * Web	-.187	.534	-.351	.726	-1.233	.859
(4300,4400] * Locative * Web	.011	.533	.020	.984	-1.035	1.056
(4400,4500] * Locative * Web	.185	.530	.350	.727	-.854	1.225
(4500,4600] * Locative * Web	-.074	.532	-.138	.890	-1.115	.968
(4600,4700] * Locative * Web	-.035	.530	-.066	.947	-1.073	1.003
(4700,4800] * Locative * Web	-.298	.530	-.563	.574	-1.338	.741
(4800,4900] * Locative * Web	-.311	.528	-.588	.556	-1.347	.725
(4900,5000] * Locative * Web	-.524	.528	-.991	.322	-1.559	.512
(5000,5100] * Locative * Web	-.574	.528	-1.087	.277	-1.608	.461
(5100,5200] * Locative * Web	-.320	.530	-.603	.546	-1.358	.719
(5200,5300] * Locative * Web	-.520	.534	-.973	.331	-1.566	.527
(5300,5400] * Locative * Web	-.889	.542	-1.642	.101	-1.951	.172
(5400,5500] * Locative * Web	-.736	.539	-1.367	.172	-1.792	.319
(5500,5600] * Locative * Web	-.752	.540	-1.393	.164	-1.811	.306
(5600,5700] * Locative * Web	-.983	.542	-1.814	.070	-2.045	.079
(5700,5800] * Locative * Web	-.875	.541	-1.618	.106	-1.936	.185
(5800,5900] * Locative * Web	-1.307	.561	-2.331	.020	-2.406	-.208
(5900,6000] * Locative * Web	-.967	.558	-1.735	.083	-2.061	.126
(6000,6100] * Locative * Web	-1.070	.561	-1.910	.056	-2.169	.028
(6100,6200] * Locative * Web	-1.103	.570	-1.936	.053	-2.220	.014
(6200,6300] * Locative * Web	-.723	.567	-1.275	.202	-1.834	.388
(6300,6400] * Locative * Web	-.821	.575	-1.427	.153	-1.949	.306
(6400,6500] * Locative * Web	-.492	.580	-.848	.396	-1.628	.645
(6500,6600] * Locative * Web	-.529	.587	-.901	.368	-1.679	.621
(6600,6700] * Locative * Web	-.308	.589	-.523	.601	-1.463	.847
(6700,6800] * Locative * Web	-.473	.597	-.792	.428	-1.644	.698
(6800,6900] * Locative * Web	-.406	.602	-.674	.501	-1.587	.775
(6900,7000] * Locative * Web	-.322	.620	-.519	.603	-1.538	.894
(7000,7100] * Locative * Web	.088	.636	.138	.890	-1.158	1.334
(7100,7200] * Locative * Web	.735	.649	1.132	.258	-.537	2.007
(7200,7300] * Locative * Web	.719	.683	1.053	.293	-.619	2.057
(7300,7400] * Locative * Web	.728	.680	1.072	.284	-.604	2.060

(7400,7500] * Locative * Web	.482	.678	.711	.477	-.847	1.811
(7500,7600] * Locative * Web	.996	.701	1.420	.156	-.379	2.370
(7600,7700] * Locative * Web	.321	.739	.435	.664	-1.127	1.769
(7700,7800] * Locative * Web	-.292	.792	-.369	.712	-1.844	1.260
(7800,7900] * Locative * Web	-.336	.798	-.421	.674	-1.899	1.227
(100,200] * Passive * Web	.079	.539	.147	.883	-.978	1.136
(200,300] * Passive * Web	-.364	.534	-.681	.496	-1.411	.684
(300,400] * Passive * Web	-.367	.539	-.681	.496	-1.422	.689
(400,500] * Passive * Web	-.593	.543	-1.094	.274	-1.657	.470
(500,600] * Passive * Web	-.657	.551	-1.191	.233	-1.737	.424
(600,700] * Passive * Web	-.288	.546	-.528	.598	-1.357	.781
(700,800] * Passive * Web	-.272	.545	-.499	.618	-1.339	.796
(800,900] * Passive * Web	.133	.540	.247	.805	-.925	1.192
(900,1000] * Passive * Web	.436	.543	.804	.421	-.627	1.500
(1000,1100] * Passive * Web	.316	.536	.589	.556	-.735	1.367
(1100,1200] * Passive * Web	-.073	.535	-.137	.891	-1.123	.976
(1200,1300] * Passive * Web	.016	.532	.030	.976	-1.028	1.059
(1300,1400] * Passive * Web	.003	.530	.006	.995	-1.035	1.042
(1400,1500] * Passive * Web	.248	.534	.465	.642	-.798	1.294
(1500,1600] * Passive * Web	-.173	.532	-.325	.745	-1.215	.869
(1600,1700] * Passive * Web	-.169	.532	-.318	.751	-1.211	.873
(1700,1800] * Passive * Web	-.328	.532	-.617	.538	-1.370	.714
(1800,1900] * Passive * Web	-.198	.531	-.373	.709	-1.238	.842
(1900,2000] * Passive * Web	-.059	.529	-.111	.912	-1.095	.978
(2000,2100] * Passive * Web	-.272	.530	-.513	.608	-1.310	.767
(2100,2200] * Passive * Web	.072	.534	.135	.892	-.974	1.118
(2200,2300] * Passive * Web	-.113	.534	-.212	.832	-1.161	.934
(2300,2400] * Passive * Web	-.225	.537	-.418	.676	-1.278	.828
(2400,2500] * Passive * Web	.104	.532	.195	.846	-.939	1.146
(2500,2600] * Passive * Web	.018	.532	.033	.973	-1.025	1.061
(2600,2700] * Passive * Web	.234	.532	.441	.659	-.808	1.277
(2700,2800] * Passive * Web	.057	.534	.107	.915	-.989	1.103
(2800,2900] * Passive * Web	-.266	.538	-.494	.622	-1.320	.789
(2900,3000] * Passive * Web	-.367	.538	-.683	.495	-1.421	.687
(3000,3100] * Passive * Web	-.255	.535	-.477	.634	-1.304	.794
(3100,3200] * Passive * Web	.059	.536	.110	.913	-.993	1.110
(3200,3300] * Passive * Web	.000	.534	.001	.999	-1.046	1.047
(3300,3400] * Passive * Web	-.140	.538	-.259	.795	-1.194	.915
(3400,3500] * Passive * Web	-.119	.536	-.222	.824	-1.169	.931
(3500,3600] * Passive * Web	-.032	.534	-.060	.952	-1.079	1.014
(3600,3700] * Passive * Web	.264	.531	.498	.618	-.775	1.304
(3700,3800] * Passive * Web	.427	.530	.806	.420	-.612	1.466
(3800,3900] * Passive * Web	.488	.535	.912	.362	-.560	1.536
(3900,4000] * Passive * Web	.184	.537	.343	.732	-.869	1.237
(4000,4100] * Passive * Web	.629	.530	1.186	.236	-.411	1.668
(4100,4200] * Passive * Web	.509	.534	.952	.341	-.538	1.556
(4200,4300] * Passive * Web	.905	.533	1.699	.089	-.139	1.949
(4300,4400] * Passive * Web	.915	.535	1.710	.087	-.134	1.963
(4400,4500] * Passive * Web	1.015	.534	1.900	.057	-.032	2.061
(4500,4600] * Passive * Web	.747	.535	1.396	.163	-.302	1.797
(4600,4700] * Passive * Web	.742	.535	1.387	.166	-.307	1.791
(4700,4800] * Passive * Web	.578	.536	1.078	.281	-.472	1.627
(4800,4900] * Passive * Web	.459	.538	.853	.394	-.596	1.513

(4900,5000] * Passive * Web	.427	.537	.795	.427	-.626	1.480
(5000,5100] * Passive * Web	.227	.545	.416	.677	-.841	1.295
(5100,5200] * Passive * Web	.334	.551	.606	.545	-.746	1.413
(5200,5300] * Passive * Web	.355	.548	.648	.517	-.719	1.428
(5300,5400] * Passive * Web	-.088	.553	-.159	.874	-1.172	.996
(5400,5500] * Passive * Web	-.197	.554	-.355	.723	-1.283	.890
(5500,5600] * Passive * Web	-.461	.560	-.822	.411	-1.559	.638
(5600,5700] * Passive * Web	-.564	.563	-1.002	.317	-1.667	.539
(5700,5800] * Passive * Web	-.115	.550	-.210	.834	-1.192	.962
(5800,5900] * Passive * Web	-.194	.555	-.350	.726	-1.283	.894
(5900,6000] * Passive * Web	.015	.561	.027	.978	-1.085	1.116
(6000,6100] * Passive * Web	-.260	.567	-.459	.646	-1.372	.851
(6100,6200] * Passive * Web	.035	.570	.061	.951	-1.082	1.152
(6200,6300] * Passive * Web	.026	.578	.044	.965	-1.107	1.159
(6300,6400] * Passive * Web	.139	.586	.236	.813	-1.010	1.287
(6400,6500] * Passive * Web	.486	.596	.815	.415	-.682	1.654
(6500,6600] * Passive * Web	.621	.605	1.026	.305	-.566	1.808
(6600,6700] * Passive * Web	.542	.605	.895	.371	-.644	1.728
(6700,6800] * Passive * Web	.388	.619	.627	.531	-.825	1.601
(6800,6900] * Passive * Web	.610	.639	.954	.340	-.643	1.863
(6900,7000] * Passive * Web	1.024	.641	1.597	.110	-.232	2.281
(7000,7100] * Passive * Web	.883	.650	1.358	.174	-.391	2.158
(7100,7200] * Passive * Web	1.798	.658	2.732	.006	.508	3.088
(7200,7300] * Passive * Web	1.852	.680	2.725	.006	.520	3.184
(7300,7400] * Passive * Web	1.384	.666	2.078	.038	.078	2.690
(7400,7500] * Passive * Web	.880	.653	1.349	.177	-.399	2.160
(7500,7600] * Passive * Web	.654	.675	.969	.333	-.669	1.976
(7600,7700] * Passive * Web	.410	.721	.568	.570	-1.005	1.824
(7700,7800] * Passive * Web	.697	.716	.973	.331	-.707	2.101
(7800,7900] * Passive * Web	.644	.727	.885	.376	-.781	2.068
